# Supplementary material for: Lively Minds: improving health and development through play–a randomised controlled trial evaluation of a comprehensive ECCE programme at scale in Ghana
Source: BMJ Open. 2022 Oct 13;12(10):e061571. doi: 10.1136/bmjopen-2022-061571 (PMC9562311; doi:10.1136/bmjopen-2022-061571)
Supplement: Supplementary data [file bmjopen-2022-061571supp001.pdf]

Supplementary materials

This appendix has been provided by the authors to give readers additional information about the study.

Supplement to: "*Lively Minds: Improving health and development through play – a randomised controlled trial evaluation of a comprehensive ECCE programme at scale in Ghana*"

Augsburg B, *et al. BMJ Open* 2022; 12:e061571. doi: 10.1136/bmjopen-2022-061571

S1. GES Lively Minds implementation model:

This supplementary material provides additional information to the intervention description.

1. Regional GES team, with support from Lively Minds NGO, conducts sensitisation of District level GES officials, introducing the program to them, explaining its importance and securing their commitment to implementing it. The District GES appoints a "Play Scheme" (PS) team consisting of District GES staff tasked with leading implementation of the program in all public KGs in the district.
2. Regional GES team conduct 2-day workshop with the District level GES PS team on how to run and sustain the programme.
3. The District GES PS team conducts sensitisation with schools in the district, which identify KG teachers that meet participation criteria to receive training of trainers[i]. These KG teachers along with the Head Teachers from the schools in which they teach attend a 5.5-day Training of Trainers workshop in which they are introduced to the GES Lively Minds programme, how to mobilise mothers to participate in the program, how to train the mothers and how to run the program in their KGs.
4. KG teachers conduct 2 community meetings to introduce the programme and ask for women in the community to volunteer to implement the PS at the KG during school time[ii]. The women who express interest are trained by the KG teachers over the course of 8 sessions under the supervision of the District PS team and Lively Minds NGO representative.
5. Play Schemes begin in KGs – the trained mothers run the Play Schemes in the KGs at least 4 times per week in 2-hour sessions, supervised by the KG teacher. These mothers also attend a monthly parenting workshop led by the KG teacher.

There is ongoing supervision of the KG teachers and Play Scheme implementation within KGs as well as of the parenting courses by the District PS team. This is done through regular unannounced visits by District GES PS team members who complete monitoring forms to record their findings during the visit. District GES PS Team delivers top-up training for the KG teachers and Head Teachers of participating schools twice per term.

[i] The school must have 2 KG teachers in place who are on permanent contracts with GES and speak the local language fluently.
[ii] There is no requirement that the participants are mothers, though most of those who sign up have young children.

Augsburg B, *et al. BMJ Open* 2022; 12:e061571. doi: 10.1136/bmjopen-2022-061571

S2. Model consent form – Primary Caregiver:

Consent Form for Primary Caregivers <u>(Primary Caregiver Survey)</u>
INNOVATIONS FOR POVERTY ACTION
Lively Minds Evaluation Extension

<u>Consent Form for Primary Caregivers (Primary Caregiver Survey)</u>

Study Title: Evaluation of a Pre-school Program Implemented at Scale in Ghana (Protocol ID NO: GHC-ERC Number: 028/09/21)

Good day, my name is ___________________________I work for Innovations for Poverty Action (IPA), a research and policy non-profit organization that discovers and promotes effective solutions to global poverty problems.

Purpose of the study

IPA is working with researchers at the Institute for Fiscal Studies (UK), Yale University (USA), University of Pennsylvania (USA) and the University of Ghana to learn more about young children and about how we can improve children's learning outcomes. This study is paid for the by United States Agency for International Development, and the Medical Research Council of the United Kingdom.

We have received official permission from the Ghana Education Service to conduct this survey. We are inviting you and your children to participate in this study.

Procedures

If you choose to participate, you will be asked to complete a survey about your background and household information, caregiver's school engagement activities, household assets, household economic activities, agricultural activities and food insecurity, household savings and credit, household economic shocks and risks, caregiver and children time use, caregiver social capital, school choice and beliefs about quality education, water, sanitation, and hygiene practices, parent-child relationship, caregiver cognitive skills, mental health and wellbeing, caregiver empowerment, children behaviours, and caregiver volunteering activities. This survey will take about 1 hour and 30 minutes of your time.

If you agree that your children participate in this research, they will be asked to participate in an assessment on numeracy, literacy, and social-emotional skills at their school. If you agree that your children participate in this research, they will be asked to participate in an assessment on numeracy, literacy, and social-emotional skills at their school through play-based tasks and take basic anthropometric measurements (i.e. height, weight, arm circumference) - always in the presence of the child's teacher or adult in a similar role. Enumerators will also observe teaching and take inventory of classroom practices. As part of this, we will record a short video of a class while it is in session.

You and your child may be contacted to participate in a follow-up survey or another study at a future date, but you are free to decline participation in the follow-up survey or another study if you wish.

We hope to record a portion of this interview for quality control purposes. This is voluntary, and you are free to decline if you do not wish to be recorded. All video and audio recordings will be destroyed after five years.

We wish to record the GPS coordinates of this interview for tracking purposes. This does not pose any additional risk to you and is voluntary, so, you are free to decline if you feel uncomfortable.

Confidentiality

The responses you and your children provide will be kept confidential to the extent possible and will only be accessible to the research team and individuals from IPA who oversees the research. Only data or information that does not identify your teachers and pupils in any way may be (a) used for research and academic publications or (b) shared with other people or organizations. It will not be possible to know that a given answer has been provided by your or your children.

While we will take all precautions to prevent this, in the event that there is an outside breach of confidentiality of the data, you will be contacted and informed of the event and the steps being taken to recover your information.

The legal basis for collecting and processing this information in the United Kingdom is “legitimate interests” (Article 6(1)(f)) of the General Data Protection Regulation 2018 and in Ghana is Data Protection Act, 2012 (ACT 843)

Voluntary Participation

Participation is completely voluntary. You and your children are free to (a) decline to participate; (b) withdraw from the study at any time, and (c) skip any question they feel uncomfortable answering without penalty or loss of any existing benefits for doing so.

Risks and Benefits

There are no anticipated risks from taking part in this survey. However, there may be inconvenience and loss of time in participating in this study. All data collection activities will be conducted in strict adherence to the covid-19 protocols including wearing nose masks to reduce the likelihood of either party contracting the virus. There are no direct benefits to you and your children from participating in the survey. However, the information you would provide will help us design an effective education policy in Ghana.

There are no direct benefits to you from participating in the survey. However, the information you would provide will help us design an effective education policy in Ghana.

Contact Information

If you have questions about the research study, you can contact IPA at +233 050 251 4712 and ask for Murphy Edro, the Research Associate. You can also contact the lead researchers directly by emailing them at ghanasurvey@ifs.org.uk.If you have any questions about your rights as a research participant, or complaints, contact Ghana Health Service Ethics Committee at 0503539896 (ask for Nana Abena Apatu) or by emailing them at ethics.research@ghsmail.org. You can also contact UCL Ethics by emailing ethics@ucl.ac.uk.

Do you have any questions that I can answer?

PARTICIPANTS’ STATEMENT

I acknowledge that I have read or have had the purpose and contents of the Participant’s Consent and Information Sheet read and all questions satisfactorily explained to me in a language I understand (____). I fully understand the contents and any potential implications as well as my right to change my mind (i.e., withdraw from the research) at any point. I understand that if I withdraw before January 2025, I can ask for my data not to be used.

For participation in this research:

1. |___| I voluntarily agree to be part of this research.
2. |___| I voluntarily agree for my children only to be part of this research
3. |___| I voluntarily agree for me and my children to be part of this research
4. |___| I don’t agree with any of us being part of this research.

Augsburg B, *et al. BMJ Open* 2022; 12:e061571. doi: 10.1136/bmjopen-2022-061571

For audio recording:

1. |__| I voluntarily agree to the audio recording for me only
2. |___| I voluntarily agree to the audio recording for my children only
3. |___| I voluntarily agree to the audio recording for me and my children
4. |___| I don't agree with the audio recording for any of us

For GPS records, I voluntarily agree to the taking of GPS records for my dwelling. |__| Yes |__|No

Name of Participant____

Participant's Signature______________________________________ OR Thumb Print_________________________________

Date____________________________________

INTERPRETERS' STATEMENT

I interpreted the purpose and contents of the Participant's Consent and Information Sheet to the afore named participant to the best of my ability in the (_________________________________) language to his proper understanding. All questions, appropriate clarifications sort by the participant and answers were also duly interpreted to his/her satisfaction.

Name of Interpreter____

Interpreter's Signature______________________________________ OR Thumb Print___________________

Date____________________________________

STATEMENT OF WITNESS

I was present when the purpose and contents of the Participant Consent and Information Sheet was read and explained satisfactorily to the participant in the (__________________________________) language, which s/he understood. I confirm that s/he was given the opportunity to ask questions/seek clarifications and same were duly answered to his/her satisfaction before voluntarily agreeing to be part of the research.

Name of Witness____

Witness' Signature_______________________________________ OR Thumb

Print___________________________

Date____________________________________

INTERVIEWER STATEMENT AND SIGNATURE

I certify that the participant has been given ample time to read and learn about the study. All questions and clarifications raised by the participant have been addressed.

Interviewer's name____

Signature____

Date____________________________________

Augsburg B, *et al. BMJ Open* 2022; 12:e061571. doi: 10.1136/bmjopen-2022-061571

S3. Model information sheet – Primary Caregiver:

Evaluation of a pre-school program implemented at scale in Ghana

This study has been approved by the UCL Research Ethics Committee (Project ID Number): 21361/001

PARTICIPANT INFORMATION SHEET

Introduction

This document contains information to help you decide whether you would like to complete the survey. Please take your time to think about the points raised before making your decision about whether or not to participate. If there is anything that is not clear, you can ask me (the surveyor) or my supervisor. Or you can contact the researchers directly by emailing at ghanasurvey@ifs.org.uk. If you wish to raise a complaint, please see contact details below.

What is this research project for?

We are conducting this research to better understand the effectiveness of a preschool program in aiding child development, with researchers at the Institute for Fiscal Studies (UK), Yale (USA), University of Pennylvania (USA) and University of Ghana. Your participation is important to us! By participating in this study, you can help us learn more about how pre-school programs can improve child development, and make children readier to start school.

Why have I been chosen?

Your household has been chosen for this interview as you have a child attending the nearby kindergarten.

What will I be asked to do and what information will we collect?

In this survey we are collecting information on your household, as well as information on your child who has recently been enrolled in pre-school and you as the primary care giver.

The household component of the survey gathers information on demographics, your educational history as well as that of other household members, dwelling, land, livestock, assets, engagement in economic activities.

Afterward a caregiver of a selected child in this household will be interviewed separately for some information about the caregivers' background, child care practice and beliefs, social networks and physical and mental well-being, as well as some information about the development of the children in this community. I will also spend some time with one child from this household who is currently enrolled in kindergarten, to take him/her through some games, as well as measuring their height and weight. I would like to invite you to participate in this survey. Both interviews plus the games with the child combined will last about 1 hour.

Right to Refuse or Withdraw

Participation in this survey is voluntary and you can choose not to participate. If you decide not to participate or withdraw at any point, nothing will happen to you or your family.

You can withdraw your consent from the study at any time, either during this survey or after. No data will be submitted without your explicit consent, which is gathered at the beginning and end of the survey. If you wish in future to withdraw your information from the survey, or to take away your consent, you can do so at any time by contacting us at ghanasurvey@ifs.org.uk. This address will be monitored until the end of 2024.

You have the right to ask questions and have those questions answered. If you have any question about the study please ask me now. You can also call [NAME AND CONTACT NUMBER OF FIELD COORDINATOR] if you have any question later.

What are the possible benefits of taking part in this research?

There are no direct benefits to you from participating in the survey. However, the information you would provide will help us design effective programs to pre-school education in Ghana.

What are the disadvantages and/or risks of taking part in the study?

There are additional risks presented by the ongoing COVID-19 pandemic. Taking part in the survey will entail interacting with an interviewer which could increase the likelihood of you contracting the virus. We will take several measures to minimize this risk as much as possible. First, the interviewer will remain at least 2 meters away from you throughout the interview, to facilitate social distancing. Interviews will be conducted outside, if possible. You will be offered disposable masks and gloves before the interview, should you wish to wear them. The tablets we use to collect your responses will be sanitized both before and after each interview. All enumerators will have their temperature checked each day and asked to report on any symptoms they are experiencing. If enumerators feel ill, we will not send them to collect any interviews. Finally, our research team will be monitoring the local COVID-19 situation every day, and will not send enumerators to conduct any interviews if the risks of transmission are deemed unacceptably high. If you do feel ill after completing the survey, you should contact [insert up-to-date information about who to contact in local health system] . If you feel unwell, you should also contact our survey team on [NAME AND CONTACT NUMBER OF FIELD COORDINATOR] so we can take additional precautions to ensure the safety of the interviewing teams and other participants.

Privacy Notice

What is the legal basis for collecting this data?

The legal basis for collecting and processing this information in the United Kingdom is “legitimate interests” (Article 6(1)(f)) of the General Data Protection Regulation 2018. As a research organisation, we have an interest in collecting and processing your data in order to understand how pre-school programs improve child development, and the effectiveness of preschool programs when they are implemented at a large scale

How will we keep your information safe? Who will have access to it?

All information we collect is strictly confidential and will only ever be used for academic research purposes. We will not share information with anyone outside the project team, not even with your parents, friends, or any other person in the village. Even when we prepare a report about the program, we will not mention the name of any person or village that participated.

We will exert our upmost effort to ensure that the data you give us remains safe and secure and that only those on the project team will ever have access to it. While we are in Ghana collecting data, your data will be accessed by me, while I enter it into the tablet during our interview, and my supervisor and the field manager, whose role it is to check the quality of the data. While we are collecting data, your data will be stored on the secure servers of the SurveyCTO. Once data has been collected from all participants, we will delete the data from the SurveyCTO server and no-one from the data collection team will retain any access to your data.

At that point, your survey responses will be transferred outside Ghana to the United Kingdom, where it will be stored on secure servers at the Institute for Fiscal Studies. At a later date it may be transferred to and stored securely at the University of Pennsylvania and Yale. Only researchers working on this project will have access to identifiable data. Only de-identified data is used for analysis. The IFS has policies and controls in place that are

aligned with ISO27001 (an international standard in information management) to ensure that your data is not lost, accidentally destroyed, misused or disclosed, and is not accessed except by members of the research team in the proper performance of their duties. This means that all information is handled according to its classification (for example personal data, publicly available data) and that access to the data is restricted accordingly, both through electronic and physical means. Staff are trained to ensure awareness of the importance of data security and practical knowledge of how to keep data safe. In addition, back-ups of our data are stored offsite in a secure UK location, which is ISO27001 compliant. We will store your contact information separately from any other information we hold on you. We will securely store your contact information in order for us to contact you again in the future for further surveys. At the point where there is no chance of running a further survey, we will delete all identifiable data.

How long will we keep your data?

As we would like to look at long term trends and we are holding your data for research purposes, we have not set a limit on how long we would like to keep the information you give. However, at the point where there is no chance of running a further survey, we will delete all identifiable data.

As according to Economic and Social Research Council guidelines, once the study has concluded, the data (without personal information) will be archived for other researchers to access.

What will happen with the results of this research?

The information that we gather will be analysed by the research team. It will contribute to research reports and academic papers on children's development and pre-school program effectiveness in Ghana. The reports will be available to download for free from the Institute for Fiscal Studies' website (www.ifs.org.uk). The reports will never include any information from which it would be possible to identify you.

Your rights

The UK Data Protection Act (2018) provides the following rights for individuals:

- The right to be informed
- The right of access
- The right of rectification
- The right of erasure
- The right to restrict processing
- The right to data portability
- The right to object
- Rights in relation to automated decision making and profiling

As your data will be processed for scientific research purposes as detailed above, various exceptions from these rights apply. In particular, the right to access, the right to rectification, the right to restrict processing, and the right to object do not apply, provided the appropriate safeguards are in place. In addition, the right to erasure is not absolute, and does not apply if it would seriously impair processing necessary for scientific research.

However, as explained above, if you wish to submit a request for your data to be deleted, please contact us at ghanasurvey@ifs.org.uk and your data will not be used in the study.

Contact us - Who is conducting and funding the study?

The research is conducted by researchers at the Institute for Fiscal Studies, University College London, Yale University, University of Pennsylvania and University of Ghana. We have a lot of experience conducting research on early childhood development.

If you have any questions about the research or wish to raise a complaint, you can contact the research team whose contact information is below. If you wish to raise a complaint please contact the Principal Investigator of

the research project, Sonya Krutikova who is based at the Institute for Fiscal Studies in London UK, at ghanasurvey@ifs.org.uk. If you feel that your complaint has not been handled to your satisfaction please contact the Chair of the University College London Research Ethics Committee, at ethics@ucl.ac.uk. If you are concerned about how your personal information is being processed, please contact IFS Data Protection Officer in the first instance (DataProtectionOfficer@ifs.org.uk). If you remain unsatisfied, you may wish to contact the UK Information Commissioner's Office (https://ico.org.uk/).

Who to contact in the event of a complaint?

If you have a complaint about any aspect of this study, please contact IPA or principle investigators of the study using the contact details above, or by emailing ghanasurvey@ifs.org.uk.

The current research team and the organisations they currently work for includes the researchers named below. If the research team members change as a result of staffing changes, the team will only ever include bona fide researchers of similar experience level. If the researchers working on the project move to a different organisation where the work on this project can be continued, your data will be shared with that organisation. An up-to-date list of researchers and their organisations can be found here [url for project site once ready] or can be obtained by emailing ghanasurvey@ifs.org.uk.

**Principal researcher:**	**Other researchers:**
Sonya Krutikova (IFS, sonya_k@ifs.org.uk)<br>Orazio Attanasio (Yale, orazio.attanasio@yale.edu)	Britta Augsburg (IFS, britta_a@ifs.org.uk)<br>Angus Phimister (IFS, angus_p@ifs.org.uk)<br>E.Nketiah-Amponsah (University of Ghana, enamponsah@ug.edu.gh)<br>Sharon Wolf (University of Pennsylvania, wolfs@upenn.edu)

The research project is funded the USAID. The project has been approved by UCL Research Ethics Committee (ID number **21361/001**) [ENTER DETAILS OF OTHER APPROVED IRBs HERE].

IFS Data Protection Officer

Zoe Oldfield (email: zoe_o@ifs.org.uk)

S4. Information on study primary and secondary outcomes

Figure S4.1 provides additional details on primary and secondary outcomes of the study.

Figure S4.1. Study primary and secondary outcome information

Augsburg B, *et al. BMJ Open* 2022; 12:e061571. doi: 10.1136/bmjopen-2022-061571

Domain	Instruments	Scoring and other information
**Primary outcomes**		
**Child cognitive development**	International Development and Early Learning Assessment (IDELA) tool	• Score following official guidelines provided by IDELA<br>• Score using Item Response Theory (IRT) for each of the developmental domains measured by IDELA<br>• Construct one overall score using IRT, pooling all IDELA items
	Tasks from the Harvard Laboratory for Development Studies	• Score using IRT for each of the domains measured<br>• Pool all items and score using IRT<br>• Pool with IDELA items and create domain specific and pooled scores using IRT
**Child socio-emotional development**	Strengths and Difficulties Questionnaire (SDQ) administered to the primary care giver.	• Score following official guidelines provided by the tool developers<br>• Create sub-domain scores using IRT<br>• Create overall score using IRT
**Child health**	Height<br>Weight	• Height for age and Weight for age<br>• Height for weight z-score
**Secondary Outcomes**		
**Maternal mental health and knowledge of ECCE**		
Caregiver mental health	Kessler-10	• Construct a total raw score following tool developer's guidance<br>• Construct a score using IRT
	Warwick-Edinburgh Mental Wellbeing Scale (WEMWBS)	• Construct a total raw score following tool developer's guidance<br>• Construct a score using IRT
Caregiver knowledge of ECCE practices	Sub-set of items from the Knowledge of Infant Development Inventory (KIDI)	• Raw score for number correct items<br>• Factor score constructed using IRT
**Parent-child investment**		
Home Environment	Family Care Indicators Questionnaire (FCI) and the Home Observation for Measurement of the Environment (HOME)	• Construct a total raw score following tool developer's guidance<br>• Construct a score using IRT
Caregiver-child relationship	Child Parent Relationship Scale (CPRS)	• Construct a total raw score following tool developer's guidance<br>• Construct a score using IRT
Caregiver instructional practices	Observation checklist; caregiver report	• Factor scores constructed using IRT: separate factor scores for observed and caregiver report items and then overall factor combining observed and caregiver report items.
**Teacher knowledge, motivation and teaching quality**		
Classroom quality	Classroom observation checklist	• Raw score of nr of conditions fulfilled out of a list of 11 (e.g. there are books in the classroom, adequate lighting, backboard etc.)<br>• Factor score constructed using IRT
Teacher practices	Classroom observation checklist	• Separate raw scores and a combined raw score for number of practices observed out of a list of 15 relating to behavior management and instructional practice.<br>• Factor scores for each of the above constructed using IRT
Teacher burn-out	Maslach burn-out battery	• Construct a total raw score following tool developer's guidance<br>• Construct a score using IRT
Teacher mental health	Kessler-10	• Construct a total raw score following tool developer's guidance<br>• Construct a score using IRT
Teacher well-being	Warwick-Edinburgh Mental Wellbeing Scale (WEMWBS)	• Construct a total raw score following tool developer's guidance<br>• Construct a score using IRT
**WASH practices in the home**	Caregiver report (including list randomization for sensitive behaviour) and interviewer observation	• Availability of WASH infrastructure: toilet, handwashing facility (with soap)<br>• WASH practices:<br>o Handwashing of the respondents and their family members, particularly the target child.<br>o Sanitation behavior (such as open defecation) of family members
**Child health**	Mid-Upper Arm Circumferences (MUAC)	• Acute Malnutrition measured by Mid-Upper Arm Circumferences (MUAC), using cut-offs as recommended by WHO.

S5. Data Statement

WHO Data set

Primary Registry and Trial Identifying Number	ISRCTN15360698
Date of Registration in Primary Registry	17/12/2021
Secondary Identifying Numbers	AEARCTR-0008500
Source(s) of Monetary or Material Support	USAID and MRC
Primary Sponsor	The Institute for Fiscal Studies
Secondary Sponsor	N/A
Contact for Public Queries	Britta Augsbury: britta_a@ifs.org.uk<br>The Institute for Fiscal Studies<br>Tel: 020 7291 4800<br>7 Ridgmount Street<br>London<br>WC1E 7AE
Contact for Scientific Queries	Dr Sonya Krutikova (sonya_k@ifs.org.uk)<br>The Institute for Fiscal Studies<br>Tel: 020 7291 4800<br>7 Ridgmount Street<br>London<br>WC1E 7AE
Public Title	Evaluating an early childhood care and education program brought to scale in Ghana
Scientific Title	Lively Minds: Improving health and development through play - Evaluation of a comprehensive ECCE programme at scale in Ghana
Countries of Recruitment	Ghana
Health Condition(s) or Problem(s) Studied	Child development
Interventions	Play schemes for pre-school children.
Key Inclusion and Exclusion Criteria	1. Be aged between 3 and 5 years<br>2. Be enrolled or planning to enroll in the sample preschool in the coming term
Study Type	Interventional, open label cluster randomized control trial.

Date of First Enrolment	7/02/2022
Sample Size	3240 children
Recruitment Status	Incomplete
Primary Outcome	Measured 2 to 3 academic terms (6 - 9 months approximately) after treatment commences:<br>1. Child cognition as measured using the emergent numeracy, emergent literacy and executive functioning tasks in the IDELA as well as items relating to the same domains from the Harvard Laboratory for Development Studies<br>2. Child health, as measured using height/weight for age and height for weight Z score<br>3. Child socio-emotional development, as measured by the externalizing, internalizing and pro-social domains of the Strengths and Difficulty questionnaire and the socio-emotional items in the IDELA
Key Secondary Outcomes	Measured 2 to 3 academic terms (6 - 9 months approximately) after treatment commences:<br>1. Maternal mental health (Kessler-10 and WEMWBS) and knowledge of ECCE<br>2. Teacher knowledge, motivation and teaching quality (measured through classroom observations)<br>3. Parent-child investment, as measured by quality of the home environment (FCI and HOME) and the parent-child relationship (CPRS)<br>4. WASH practices in the home<br>5. Acute Malnutrition measured by MUAC
Ethics Review	Study protocols have been approved by ethics boards at University College London (IRB 21361/001), Yale University (IRB 2000031549) and Ghana Health Service Ethical Review Committee (IRB 028/09/21).
Completion date	September 2025
Summary Results	n/a as trial incomplete
IPD sharing statement	The data collected in the study will be publicly distributed along with critical documents (ie, protocols and questionnaires) following the publication of the primary results from the trials, which is expected to be within 24 months of the final data collection date.
