# Supplementary material for: Lively Minds: improving health and development through play–a randomised controlled trial evaluation of a comprehensive ECCE programme at scale in Ghana
Source: BMJ Open. 2022 Oct 13;12(10):e061571. doi: 10.1136/bmjopen-2022-061571 (PMC9562311; doi:10.1136/bmjopen-2022-061571)
Supplement: Author's manuscript [file bmjopen-2022-061571.draft_revisions.pdf]

BMJ Open is committed to open peer review. As part of this commitment we make the peer review history of every article we publish publicly available.

When an article is published we post the peer reviewers' comments and the authors' responses online. We also post the versions of the paper that were used during peer review. These are the versions that the peer review comments apply to.

The versions of the paper that follow are the versions that were submitted during the peer review process. They are not the versions of record or the final published versions. They should not be cited or distributed as the published version of this manuscript.

BMJ Open is an open access journal and the full, final, typeset and author-corrected version of record of the manuscript is available on our site with no access controls, subscription charges or pay-per-view fees (http://bmjopen.bmj.com).

If you have any questions on BMJ Open's open peer review process please email info.bmjopen@bmj.com

BMJ Open

Lively Minds: Improving health and development through play – a randomised controlled trial evaluation of a comprehensive ECCE programme at scale in Ghana

Journal:	*BMJ Open*
Manuscript ID	bmjopen-2022-061571
Article Type:	Protocol
Date Submitted by the Author:	03-Feb-2022

Complete List of Authors:	Krutikova, Sonya; Institute for Fiscal Studies<br>Augsburg, Britta; Institute for Fiscal Studies,<br>Dreibelbis , Robert; London School of Hygiene and Tropical Medicine, Disease Control Department; London School of Hygiene and T<br>Nketiah-Amponsah, Edward; University of Ghana,<br>Phimister, Angus; Institute for Fiscal Studies,<br>Wolf, Sharon; University of Pennsylvania, Graduate School of Education<br>Attanasio, Orazio; Yale University

Keywords:	EDUCATION & TRAINING (see Medical Education & Training), Health policy < HEALTH SERVICES ADMINISTRATION & MANAGEMENT, PRIMARY CARE, PUBLIC HEALTH

SCHOLARONE™
Manuscripts

BMJ Open Protocol

Lively Minds: Improving health and development through play – a randomised controlled trial evaluation of a comprehensive ECCE programme at scale in Ghana

Authors[a]

For peer review only

1- Institute for Fiscal Studies
2- London School of Hygiene and Tropical Medicine
3- University of Ghana
4- University of Pennsylvania
5- Yale University

Contact Details:
Sonya Krutikova: sonya_k@ifs.org.uk
Britta Augsburg (corresponding author): britta_a@ifs.org.uk
Robert Dreibelelbis: Robert.Dreibelbis@lshtm.ac.uk
Edward Nketiah-Amponsah: enamponsah@ug.edu.gh
Angus Phimister: angus_p@ifs.org.uk
Sharon Wolf: wolfs@upenn.edu
Orazio Attanasio: orazio.attansio@yale.edu

For peer review only - http://bmjopen.bmj.com/site/about/guidelines.xhtml

Sonya Krutikova[1], Britta Augsburg[1], Robert Dreibelelbis[2], Edward Nketiah-Amponsah[3], Angus Phimister[1], Sharon Wolf[4], Orazio Attanasio[1,5].

ABSTRACT

Introduction

Many children in developing countries grow up in environments that lack stimulation, leading to deficiencies in early years' development in some domains, particularly cognition and language. Various early childhood care and education programs (ECCE) have demonstrated a clear impact on these outcomes in small efficacy trials, but the sustainability of effects is mixed for interventions scaled by government as part of public policy. This study evaluates an ECCE programme found to be effective in an efficacy-trial when being taken to scale across Northern Ghana.

Methods and Analysis

This study is a cluster randomized control trial (CRCT), in which the order that districts receive the programme is randomized. A minimum sample of 3240 children and 360 schools will be recruited across 72 district-school cohort pairs. The primary outcomes are (i) multiple dimensions of child cognitive and socio-emotional development measured using the International Development and Early Learning Assessment (IDELA) tool, the Strengths and Difficulties Questionnaire, and tasks from the Harvard Laboratory for Development Studies and (ii) child health measured using height/weight for age and height for weight Z scores. Secondary outcomes include (i) maternal mental health, measured using the Kessler-10 and Warwick Edinburgh Mental Wellbeing Scale (WEMWBS) and knowledge of ECCE practices (ii) teacher knowledge, motivation, teaching quality (measured with classroom observation), (iii) parental investment measured through quality in the home environment (Family Care Index and Home Observation Measurement of the Environment) and the quality of the parent-child relationship (Child Parent Relationship Scale) (iv) WASH practices (v) acute malnutrition measured using MUAC. We will estimate unadjusted and adjusted intent-to-treat effects. We will also consider differences by district-level governance and programme readiness.

Ethics approval

Study protocols have been approved by ethics boards at University College London (IRB 21361/001), Yale University (IRB 2000031549) and Ghanaian Health Service Ethics Review Committee (IRB 028/09/21).

Trial registration number

AEARCTR-0008500, ISRCTN15360698

Keywords

Child health, child development

ARTICLE SUMMARY

Strengths and Limitations of this Study

For peer review only - http://bmjopen.bmj.com/site/about/guidelines.xhtml

- This trial assesses the effect of a programme previously demonstrated to be effective in a small efficacy trial now adopted by government and being brought to scale
- Scaling up effective interventions has proven challenging in the past; this study will provide much needed evidence on whether an intervention designed to be scalable can be effective
- A challenge in this study is measuring and adjusting for differences in programme delivery quality across different districts

INTRODUCTION

Over 250 million children worldwide under the age of 5 years are at risk of not achieving their developmental potential due to poverty, poor health and nutrition, and deficient care and stimulation[1].The majority of these children live in low- and middle-income countries (LMICs), 1.6 million in Ghana[2]. In Northern Ghana, the setting of this study, and where most families live on less than US$2 per day, 20% of under 5s are stunted and 39% of 3-4 years olds are off-track cognitively[3].

School enrolment alone does not offset these disadvantages: in spite of great global progress in primary school enrolment, around 125 million children lack age-appropriate skills[4]. Years of exposure to poor environments have adverse long-run effects on children's cognitive and socio-emotional development, health and psychological well-being, and earning potential, increasing the risk of poverty, early marriage and parenthood[5]. A recent Lancet review highlights that 'children at elevated risk for compromised development due to stunting and poverty are likely to forgo about a quarter of average adult income per year' [6].

As shown in a substantial body of evidence and increasingly recognized by policymakers, Early Childhood Care and Education (ECCE) interventions offer an opportunity to ameliorate these detrimental effects at a time of heightened developmental sensitivity and malleability[7]. The Government of Ghana (GoG) has implemented some of the most advanced ECCE policies in the region (National Early Childhood Care and Development Policy in 2004 and 2 years of public pre-primary education in 2007), resulting in universal net enrollment[8]. The GoG, however, recognizes that significant challenges remain: A third of Ghanaian pre-school (KG) children do not meet school readiness indicators[9] and deficits persist and grow through primary school, where a fourth of pupils do not meet all proficiency cut-offs[10].

In 2017/18, OA and SK led an efficacy trial of *Lively Minds* - a holistic ECCE programme which engages parents and pre-schools to achieve healthy child development of 3-5 years olds in rural Ghana and promotes inclusion of children with disabilities. The trial revealed a positive impact on child health, as well as cognitive and socio-emotional development, partly mediated by improvements in parenting practices[11].

As a result of this evidence, over the next four years the *Lively Minds* programme will be scaled by the Ghana Education Service (GES) across Northern Ghana to over 4,000 pre-schools, reaching 1.3 million children, reducing costs to $7 per child per year (less than a third of the original cost, and significantly below typical group parenting programme costs in LMICs, estimated to be US$30–35 per child[12]).

This study is a CRCT evaluating this scale-up, with randomization achieved through randomization of the order in which districts begin to receive the program. We will assess whether the impacts found at efficacy trial stage are sustained and investigate key mechanisms

For peer review only - http://bmjopen.bmj.com/site/about/guidelines.xhtml

underlying these in order to determine how implementation and cost-effectiveness could be further improved.

Objectives

The project's key research questions are as follows:

1. What is the impact of the *Lively Minds* programme at scale (the GES Lively Minds programme) on child development?
2. How does the impact of the intervention vary by child, teacher, classroom/school and district characteristics?
3. What role do intervention fidelity and compliance play (intensity of training, dosage, hygiene promotion, quality of play sessions, supervision by teachers and district education offices) and how can the design be adapted to maximise cost-effectiveness?
4. Are impacts achieved through changes within the classroom/school and/or the children's home environments, including teacher and parent behaviour?

METHODS AND ANALYSIS

Study Setting

The programme will be rolled out at scale in Northern Ghana, where prevalence of chronic malnutrition is nearly twice as high as the national average (at 33% and 19% respectively) and 60-70% of the population were classified as poor in 2017, compared to 23% nationally[13,14]. Baseline conditions among the sample in the efficacy trial reveal that one third of children (3-5 years) had acute malnutrition and had experienced diarrhoea in the last 30 days; less than a third could identify a shape such as a triangle and even fewer were able to sort figures based on colour and shape. Around half of the mothers were at risk of depression and only 13% reported that *anyone* in the household had played with the target child in the last 3 days. The children live in households of on average ten members in which only 20% ever attended school and even fewer are literate (8%). Household sanitary infrastructure is largely lacking - nearly 80% report that they defecate openly. Access to public infrastructure is dismal: one in three communities do not have access to electricity and more than half cannot be accessed for 1-2 months of the year due to lack of paved roads[15].

Intervention Description

ECCE programs are often one-dimensional, targeting *either* parents *or* pre-schools, and/or focusing on one area of child development. Lively Minds NGO (LMNGO) developed a hybrid ECCE program, designed with scalability in mind, that *simultaneously* improves the home and pre-school environments, as well as children's health alongside cognitive and socio-emotional development. This intervention was previously evaluated in an earlier study[15].

The Lively Minds intervention is designed to combat the twin problem of low KG quality and parenting practices that do not provide adequate mental stimulation. It does so by (i) training KG teachers and caregivers, empowering them to set-up and run low-cost educational weekly Play Schemes (PS) within the KGs. These PS are designed to support child learning through play. PS are also equipped with locally available handwashing facilities (tippytaps) and PS activities are specifically designed to promote regular handwashing both at homes and in the PS; (ii) training KG teachers to run a monthly group parenting course for the caregivers who run the PS, teaching caregivers simple ways to improve home-based care (nutrition, hygiene,

For peer review only - http://bmjopen.bmj.com/site/about/guidelines.xhtml

malaria prevention, stimulation practices) using local resources. Teachers are given regular training, supervision and support through Ministry of Education's (MoE) regional and district teams. There are a number of key features that make this approach innovative, transformative, cost-effective and scalable. First, the programme empowers an under-engaged population – caregivers of small children. From being marginalised and isolated, they become part of a peer group and gain confidence and standing within the community. Second, the PS intervention targets home and school environments simultaneously. Third, Lively Minds uses local manpower and existing school infrastructure, offering a platform to reach teachers, the wider education system, and marginalised caregivers in hard-to-reach communities. The programme places specific emphasis on children with disabilities through workshops with teachers and parents on inclusive education, designed to challenge taboos around disability, support enrolment of affected children, and teach PS adaptations to accommodate different disabilities.

While these features will remain at the core of *Lively Minds*, several important changes will be made to the implementation and curriculum during the scale-up phase that we propose to evaluate. During the efficacy trial stage, LMNGO maintained the ownership of the programme, leveraging the KG infrastructure for implementation in partnership with the MoE. The scale-up phase will involve transmission of ownership of the programme from LMNGO to the MoE, critical for the long-run sustainability of the programme. In preparation LMNGO are (i) transferring control of design and implementation to an MoE Working Group headed by the Chairman of the GES; (ii) conducting district-level sensitization activities, developing simple to use tools to facilitate programme management and quality assurance and conducting regular capacity building activities. In addition, LMNGO have adapted the core play-scheme and parenting workshop curricula to address weakness identified by us through the efficacy trial. The new programme is referred to as the *GES Lively Minds* (GES-LM) programme.

Trial Design

This study is an open label cluster randomized control trial, implemented through randomization of the order in which districts start to receive the GES-LM programme. See Figure 1 for flow diagram. Randomization was conducted using R version 4.1.1 by researchers at the IFS.

Figure 1. Randomization flow Diagram

[Figure 1 here]

While the programme is scaled to all districts in Northern Ghana, this study focuses on 54 districts (grouped into 9 district groups (DG)) and two academic cohorts (C) of children, combined into 72 district-cohort pairs. District groups will be enrolled into the study from January 2021 to September 2024 in 6 evaluation cohorts (EC). In each EC, one DG is allocated at random into the treatment group and one to the control. DG6 and DG7 are used first as control and later as treatment DGs, relying on different cohorts of children (in different schools). DG10 will provide the control group for EC5 and EC6 using again different academic cohorts of children in different schools. Control DGs will begin to receive the programme 2/3 terms after being enrolled into the study, after endline data collection has been completed.

Figure 2 summarizes this design and the rollout of DGs into evaluation cohorts, as well as the timeline of planned data collections. Census data collection started before the end of 2021, and baseline data collection will start in the end of January 2022 (after participant enrolment). Data

For peer review only - http://bmjopen.bmj.com/site/about/guidelines.xhtml

collection will proceed continuously through to the end of the project in early 2025. Figure 3 shows the participant timeline.

Figure 2. Project Timeline

[Figure 2 here]

Figure 3. Participant Timeline

[Figure 3 here]

For peer review only

Sample size and Sampling

Within each district, we plan to randomly sample 5 programme -eligible preschools and a minimum of 9 children who are enrolled or about to enrol in the sampled preschool. In DG6, DG7 and DG10 we will sample children and schools twice (once for each cohort of children). When we sample for a second time, we will exclude schools previously enrolled into the study. This gives us a minimum total sample of 360 preschools and 3460 children and households.

Schools will be selected at random from a complete school list in each district provided by the GES. We will exclude private and religious schools, as well as only boys' and only girls' schools, as well as schools with less than 20 enrolled students.

Our study participants will be children, and their households, that attend, or are due to attend KG. To be eligible for the study children must fulfil both of the following criteria (the same as in the efficacy trial):

1. Be aged between 3 and 5 years
2. Be enrolled or planning to enroll in the sample preschool in the coming term

We will conduct a census around schools to identify these study participants. Where more than one child is eligible within a single household we will randomly select one child to be a part of the study. Primary care givers will be asked for their consent (and to give consent on behalf of their child) before being enrolled in the study. Model consent form and information sheet for primary caregivers are provided in Supplementary Materials S1. And S2.

Outcomes

In each sampled unit (which comprises of the catchment area of a school, which we will refer to as 'village') we intend to conduct a household census, followed by 4 separate surveys/assessments. Surveys and assessments will be implemented twice - before intervention implementation (baseline) and again after three terms (endline). Surveys/assessments include: (1) child assessment, (2) Primary caregiver (PCG) Interview, (3) Kindergarten teacher interview and classroom observation, (4) community survey. These surveys will be collected by local well-trained and experienced interviewers, following the district group timeline as shown in Figure 2.

In order to gather evidence on district-level preparedness for the programme (for later heterogeneity analyses), we additionally plan to conduct a survey of the district official in

For peer review only - http://bmjopen.bmj.com/site/about/guidelines.xhtml

charge of preschool programmes in each district once around the time of the first data collection in that district. We will also collect secondary data available on districts, such as languages spoken, or voter turnout.

We have three sets of primary outcomes

1. Child cognitive development measured using the International Development and Early Learning Assessment (IDELA) tool[15] and tasks from the Harvard Laboratory for Development Studies, all previously used in Ghana.
2. Child socio-emotional development measured using Strengths and Difficulties Questionnaire (SDQ)[16] administered to the primary care giver.
3. Child health measured using height/weight for age and height for weight Z scores, following WHO guidelines.

Our secondary outcomes are as follows:

1. Maternal mental health, measured used the Kessler-10 and WEMWBS and knowledge of ECCE practices
2. Teacher knowledge, motivation and teaching quality, measured using classroom observation
3. Parental investment measured through quality in the home environment (Family Care Index and Home Observation Measurement of the Environment) and the quality of the parent-child relationship (CPRS)
4. WASH practices in the home (e.g. hand washing practices, toilet facilities, open defecation practices).
5. Acute malnutrition measured using middle upper arm circumference. Beyond being of interest in its own right, this is the health outcome used in the efficacy trial[15], which will allow us to compare outcomes across both trials.

In selecting measures for our key outcomes, we prioritised those which have already been validated and used in the study context (for example IDELA[17, 18]) or in a context that is similar. Since very little related work has been conducted in our study context, this was not possible for all outcomes that we want to capture. For these outcomes we next identified measures which have been widely used and validated across multiple contexts, for example the Kessler-10 and FCI. We will adapt these measures to the study context following best practice[19]: tools will be reviewed for socio-cultural relevance and linguistic accuracy through consultation with local professionals and modified accordingly; tools will be translated and back-translated into the all of the main languages spoken in the study communities. Full validation of the tools before administration of the baseline is beyond the scope of this study. However, all outcome measurement tools will be piloted on small samples in the study area, feedback on performance will be collected from the surveyors and further adaptations will be made. As part of the training the survey team will be required to achieve inter-rater reliability of at least 0.8 (kappa statistic) on the child assessment and classroom observation tools. Once data are collected we will assess reliability and validity of the outcome measures by examining whether any items have a lot of missing responses, items show sufficient variability, correlations with other variables go in the expected direction, measures exhibit sufficient degree of internal consistency (Cronbach's alpha of at least 0.7). We will also assess dimensionality of the measures using exploratory and confirmatory factor analysis and functioning of individual items using Item Response Theory to generate item characteristics curves.

For peer review only - http://bmjopen.bmj.com/site/about/guidelines.xhtml

In addition to the primary and secondary outcomes, planned data collection will be used to conduct a prospective process evaluation of the GES-LM programme. Specifically, this process evaluation will be used to assess intervention fidelity and quality, completeness, exposure (for both teachers and caregivers), and inclusiveness of the intervention[20]. Process evaluation will be informed by a robust Theory of Change developed collaboratively with all project stakeholders in early 2022[21] and will align with MRC guidelines on the process evaluation of complex interventions[22].

Data management protocols and Data Statement

For details on data management please refer to the project's management registration: https://ifs.org.uk/uploads/Research%20registraion%20form.pdf. Data availability is outlined in Supplementary Material S3.

Sample Size and Power

As our design does not deviate in a substantive way from a conventional two-arm cluster randomized control trial design (CRCT), we use standard CRCT methods to assess statistical power. We leverage data available from the efficacy trial to better inform how covariates increase our power to detect effects.

We calculate minimum detectable effects (MDEs) using the following formula[19]:

$$MDE = \left(\frac{\left(t_{\frac{\alpha}{2}} + t_{\beta}\right)^2 \sigma^2 \left(m\rho\left(1 - R_c^2\right) + (1-\rho)(1 - R_p^2)\right)}{n} \right)^{\frac{1}{2}} \quad (1)$$

α denotes the significance level, β power, ρ unconditional ICC, t is are the critical values of the t-distribution, σ is the standard deviation of the outcome, n is children per arm, m is children per cluster, R_c^2 is proportion of cluster level variance component explained by covariates and R_p^2 is the individual equivalent. One can interpret the effect of R_c^2 and R_p^2 as altering the "variance inflation factor" of the power calculation – when they equal zero the formula reduces to the standard CRCT one.

We estimate the intra-cluster correlation (ICC) for each outcome data using data from the efficacy trial (ranging from 0.011 to 0.110). Note we cannot conduct the same exercise for all of out primary outcomes, as some were not included in the original trial. The minimum detectable effects for some of our primary and secondary outcomes measured at the child/household level are shown in Table 1. The power to detect effects at the school level is significantly more limited.

Table 1. Minimum Detectable Effects

Outcome	MDE
Cognition factor score	0.118
Middle Upper Arm Circumference	0.132
Knowledge Infant Development Index (KIDI)	0.125
Family Care Index (FCI)	0.227

For peer review only - http://bmjopen.bmj.com/site/about/guidelines.xhtml

Parent-child interaction (PRC)	0.132

Note: Each outcome controls for the its baseline value only.

Analysis Plan

Intention to Treat

For our primary and secondary outcomes, we will estimate an intent-to-treat effect of the program. Our design is the same as a cluster randomized control trial stratified by evaluation cohort. As such, we follow the standard estimation technique for two-way stratified cluster-randomised control trials, For the outcome variable *Y* of individual *i* in district *j* at date *t,* we estimate the following:

$$Y_{ijt} = \mu + \gamma_t + \theta\, Treatment_{ijt} + \boldsymbol{\beta}\, \boldsymbol{X_{ijt}} + \varepsilon_{ij}$$

Where γ_t is a evaluation cohort fixed effect, $Treatment_{ijt}$ is binary indicator variable equal to 1 if individual *i* is in a treatment district at date *t*, $\boldsymbol{X_{ijt}}$ is a set of controls variables. The treatment effect we identify is θ, and standard errors will be clustered at the school level.

We will compute the Romano-Wolf stepdown p-values to adjust for multiple hypothesis testing. Hypotheses will be arranged within families of outcomes - i.e. each set of research questions separately.

The set of controls $\boldsymbol{X_{ijt}}$ will be identified using a double-lasso procedure of the covariates we collect as part of our data collection. We will present estimates of the treatment effect both with and without these control variables. In addition to those controls that the double-lasso procedure generates, we will use (at a minimum) the following controls:

- Mother age (quadratically)
- Mother Education attainment
- Tester fixed effects (where relevant)
- Time between baseline and endline measurements
- Child Age
- Child Sex at birth
- Baseline value of the outcome variable of interest

Heterogeneity

We will explore heterogeneity in treatment effect in two distinct ways.

We are first interested in considering treatment heterogeneity by baseline characteristics of the child, their home environment, and features of the environment at the preschool and district-level. Given the large number of relevant dimensions, we will use recent methodological advances[24] to assess heterogeneity across all of these dimensions in a rigorous and data-driven way.

Second, we will assess how study impacts vary by implementation fidelity and compliance in different dimensions. Given intervention fidelity measures are only observed in treatment districts, we will also examine heterogeneity from the data collected as part of the district official survey, focusing on those covariates that are most predictive of high fidelity in treatment districts.

Stopping Rules

For peer review only

Whilst there is always a risk of unintended consequences in all types of trials, in this sort of intervention such a risk is minimal. However, if there is any clear evidence of harm then the study will halt under international ethical guidelines for medical research.

Additional Analysis

This is a large study with many collaborators, and the data gathered will be able to answer more scientific questions than those outlined in this protocol. The study team expects to conduct and publish such additional analyses.

Patient and Public Involvement

Patients or the public were not involved in the design of our research.

ETHICS AND DISSEMINATION

The trial is overseen by independent ethics review boards, which have reviewed the study protocols. Particular consideration will be given to potentially vulnerable people or groups, especially children, and informed consent will be acquired by IPA Staff from all parents of participating children before the commencement of any data collection, and that they can stop participating at any time without providing a reason. Model consent forms are available upon request. Any reports of abuse or dangers to children will be reported to relevant local authorities.

Important protocol modifications will be communicated to all relevant parties (e.g. investigators, REC/IRBs, trial participants, trial registries, journals).

Data will be collected, handled, transferred and secured in a manner which aligns with international best practice. The IFS information security management system is ISO27001 compliant.

We will publish findings through (a) top academic journals in economics and developmental science; (b) presentations at conferences in Ghana, US, and Europe; (c) publication of high impact policy briefs for the wider public; (d) leaflets to communicate findings to participating communities; and (e) partner's websites and social media.

Whilst there is always a risk of unintended consequences in all types of trials, in this sort of intervention such a risk is minimal. However, if there is any clear evidence of harm then the study will halt under international ethical guidelines for medical research.

Contributorship Statement

SK, BA, SW, ENA, RD and OA conceptualized the design and methods of the study and revised the manuscript and approved the final manuscript as submitted. AP drafted the initial manuscript, BA revised the manuscript and approved the final manuscript as submitted. All authors provided input and guidance on study design. All authors approved the final manuscript as submitted and agree to be accountable for all aspects of this systematic review

Competing interests

All authors have no competing interests to declare.

Funding

This study is funded by USAID (grant number 7200AA21FA00016). This funding source had no role in the design of this study and will not have any role during its execution, analyses, interpretation of the data, or decision to submit results.

For peer review only - http://bmjopen.bmj.com/site/about/guidelines.xhtml

Data sharing

Data to be generated within this study will be made available in the UK Data Archive within two years after completing the endline survey. For articles published earlier than that, data and programming files will be uploaded as supplementary information.

Word count: 3,653

For peer review only

For peer review only - http://bmjopen.bmj.com/site/about/guidelines.xhtml

REFERENCES

1 Grantham-McGregor, S, YB Cheung, S Cueto, P Glewwe, L Richter, B Strupp, *Lancet*, 2007;369:60–70.
2 Lu, C, M Black, L Richter, *The Lancet Global Health*, 2016 Dec; 4(12).
3 MICS snapshot report Ghana 2017-18.
4 UNESCO, EFA Global Monitoring Report, 2013/14)
5 Gertler, P, J Heckman, R Pinto, A Zanolini, C Vermeerch, S Walker, SM Chang, S Grantham-McGregor, *Science*. 2014 May 30; 344(6187): 998–1001.
6 Richter, LM, B Daelmans, J Lombardi, J Heymann, FL Boo, JR Behrman, C Lu, JE Lucas, R Perez-Escamilla, T Dua, ZA Bhutta, The Lancet. 2017;389(10064):103-18.
7 Shonkoff, JP, DA Phillips, National Academy Press, 2016.
8 Ministry of Education Ghana, 'Education Strategic Plan 2018-2030'.
9 McCoy,DC , ED Peet, M Ezzati, G Danaei, M Black, CR Sudfeld, W Fawzi, G Fink, *PLoS Medicine*, 2016, 13(6).
10 Ministry of Education, Ghana Education Service, 'National Education Assessment' Technical Report 2016.
11 Attanasio, O, B Caeyers, L Cardona-Sosa, S Cattan, S Krutikova, P Leighton (2020). https://www.ifs.org.uk/uploads/Impact%20report_submitted.pdf
12 Horton, S, MM Black. (2017), Chapter 24 in: Bundy DAP, ND Silva, S Horton, et al., editors. Child and Adolescent Health and Development. 3rd edition. Washington (DC).
13 USAID, 2018"Ghana: Nutrition Profile". https://www.usaid.gov/sites/default/files/documents/1864/Ghana-Nutrition-Profile-Mar2018-508.pdf.
14 Ghana Living Standard Survey, 2016/17.
15 Pisani, L, I Borisova, AJ Dowd (2018), *International Journal of Educational Research*, 91: 1–15. https://doi.org/10.1016/j.ijer.2018.06.007
16 Goodman, R., *Journal of Child Psychology and Psychiatry*, Jul 1997;38(5):581-6.
17 Amadu, S, O Attanasio, B Caeyers, S Cattan, L Cardona Sosa, S Krutikova, P Leighton, 2018, IFS Report 12996. https://www.ifs.org.uk/uploads/R144.1.pdf
18 Wolf, S, M Peele, *Journal of the British Academy*, 8(s2), 71–102
19 Fernald, LCH, E Prado, P Kariger, A Raikes. 2017. *A Toolkit for Measuring Early Childhood Development on Low-and Middle-Income Countries.* Washington DC. The World Bank.
20 Saunders RP, MH Evans, P Joshi. Developing a Process-Evaluation Plan for Assessing Health Promotion Program Implementation: A How-To Guide. Health Promotion Practice. 2005;6(2):134-147. doi:10.1177/1524839904273387
21 Breuer, E., L Lee, M De Silva et al. Using theory of change to design and evaluate public health interventions: a systematic review. *Implementation Sci* 11, 63 (2015). https://doi.org/10.1186/s13012-016-0422-6
22 Process evaluation of complex interventions, UK Medical Research Council (MRC) guidance, 2014
23 McConnell, B, M Vera-Hernandez, 2015, IFS Working Paper W15/17, Going beyond simple sample size calculations: a practitioner's guide, https://ifs.org.uk/publications/7844
24 Wager, S, S Athey, *J of the American Statistical Association*, 2018: 113:523, 1228-1242.

Date and Version Number

03/02/2022, version 1

For peer review only - http://bmjopen.bmj.com/site/about/guidelines.xhtml

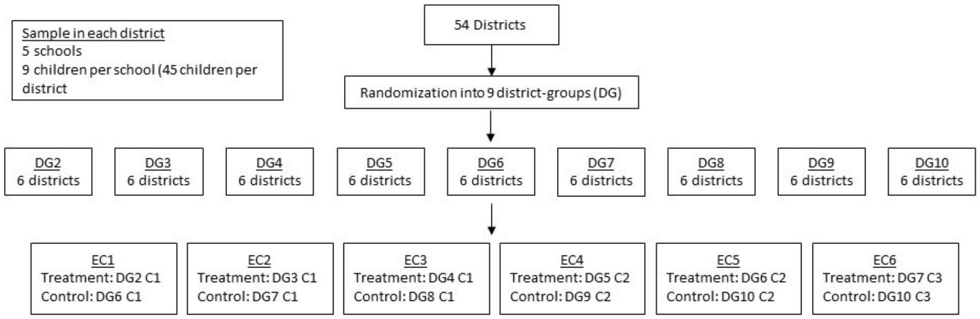

Notes: "DG" refers to district group (with each group consisting of 6 districts), "EC" refers to evaluation cohort, "C" refers to academic cohort of children

252x85mm (96 x 96 DPI)

Calendar time		Sep-21	Jan-22	May-22	Sep-22	Jan-23	May-23	Sep-23	Jan-24	May-24	Sep-24
Survey instrument prep & piloting											
DG2(Treatment)	C2	0	1	1	1	1	1	1	1	1	1
DG6 (Control)	C2	0	0	0	0	0	1	1	1	1	1
DG3(Treatment)	C2	0	0	1	1	1	1	1	1	1	1
DG7(Control)	C2	0	0	0	0	0	0	1	1	1	1
DG4 (Treatment)	C2	0	0	0	1	1	1	1	1	1	1
DG8 (Control)	C2	0	0	0	0	0	0	0	1	1	1
DG5(Treatment)	C3	0	0	0	0	1	1	1	1	1	1
DG9 (Control)	C3	0	0	0	0	0	0	0	0	1	1
DG7 (Treatment)	C3	0	0	0	0	0	0	1	1	1	1
DG10 (Control)	C3	0	0	0	0	0	0	0	0	0	1*
DG6 (Treatment)	C3	0	0	0	0	0	1	1	1	1	1
DG10 (Control)	C3	0	0	0	0	0	0	0	0	0	1*

Treatment
Control
Cohort attending KG
Data collection
0 District not exposed to intervention
1 District exposed to intervention

368x136mm (72 x 72 DPI)

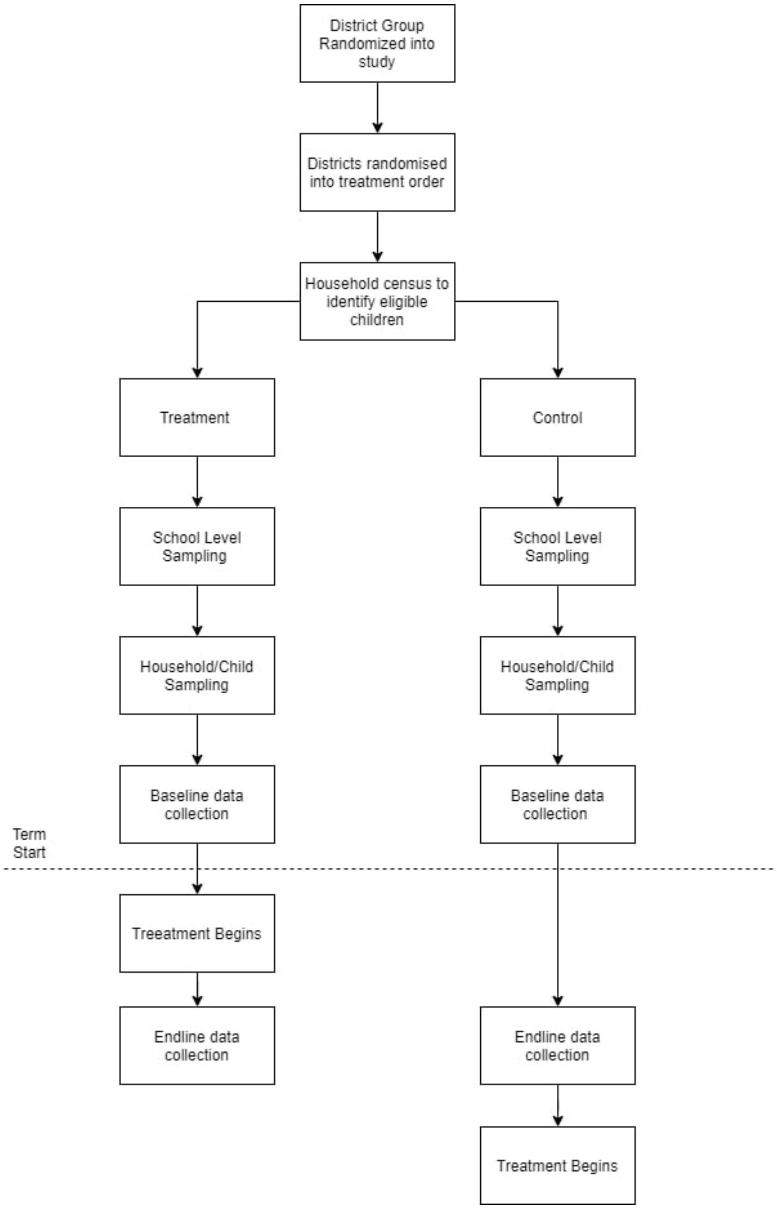

212x328mm (72 x 72 DPI)

SUPPLEMENTARY MATERIAL

S.1 Model consent form – Primary Caregiver:

Evaluation of a pre-school program implemented at scale in Ghana

This study has been approved by the UCL Research Ethics Committee (Project ID Number): 21361/001

CONSENT FOR PARENT/CAREGIVER AND CHILD

Good day, my name is [NAME OF SURVEYOR]. I work with Innovations for Poverty Action (IPA). IPA is a research organization with its head office in the US. IPA is working with researchers at the Institute for Fiscal Studies (UK), Yale (USA), University of Pennylvania (USA) and University of Ghana to learn more about young children and about how we can improve children's learning outcomes. We have received official permission from the Ghana Education Service to conduct this survey.

This form, alongside the information sheet, contains information to help you decide whether you would like to complete the survey. For more detailed information, please take your time to read, or ask me to go through with you the information sheet and privacy notice. I will leave a copy of the information sheet and privacy notice with you after the survey. Please take your time to think about the points raised before making your decision about whether or not to participate. If there is anything that is not clear, you can ask me (the surveyor) or my supervisor. Or you can contact the researchers directly by emailing at ghanasurvey@ifs.org.uk. If you wish to raise a complaint, please see contact details below.

Procedures

As part of this study, we are conducting a household survey which asks some information about your household, such as its demographics, your educational history as well as that of other household members, dwelling, land, livestock, assets, engagement in economic activities, income and consumption expenditures and prices available in your community.

Afterward we will gather information about your background, child care practice and beliefs, social networks and physical and mental well-being, as well as some information about the development of the children in this community. I will also spend some time with one child from this household who is currently enrolled in kindergarten, to take him/her through some games, as well as measuring their height and weight. I would like to invite you to participate in this survey. Both interviews plus the games with the child combined will last about 1 hour.

Confidentiality

Kindly note that all your responses and that of your child will be anonymized and will be held in confidence. All information we collect is **strictly confidential** and will only ever be used for academic research purposes. We will **never** publish any names or other information that could let people figure out who you are.

Only the researchers involved in this study and those responsible for research oversight will have access to the information you provide which identifies you or your child. You and your child's responses will be numbered and the code linking the number with your names will be in an encrypted data file.

While we are collecting data, your data will be accessed by me, while I enter it into the tablet during our interview, and my supervisor and the field manager. While we are collecting data, your data will be stored on a

For peer review only - http://bmjopen.bmj.com/site/about/guidelines.xhtml

secure server, run and managed by SurveyCTO. Once data has been collected from all participants, we will delete the data from this server and no-one from the data collection team will retain any access to your data.

After the end of data collection, your data will be securely transferred outside of Ghana to the United Kingdom, where it will be stored securely at the Institute for Fiscal Studies. At a later date, the data may be securely transferred to Yale and the University of Pennsylvania. Only members of the research team will be able to access it. In line with best practice for research, once our study is finished we will securely archive your information (without any personal information) for other researchers to use in the future.

The legal basis for collecting and processing this information in the United Kingdom is “legitimate interests” (Article 6(1)(f)) of the General Data Protection Regulation 2018.

Voluntary Participation

Your participation in this study is voluntary. If at any time, you do not want to answer a question or feel like discontinuing the survey, you are free to do so. You are free to withdraw you consent after the study by emailing us at ghanasurvey@ifs.org.uk or contacting IPA staff whose (details below). This address will be monitored until the end of 2024. Your decision will not result in any penalty. Also, refusing to participate would not affect you or your communities’ eligibility to receive any program or funds or services.

Also, even if you agree to participate in the study now, no one from IPA will be allowed to ask you or your children additional questions in the future without asking for your consent again.

Risks and Benefits

There are additional risks presented by the ongoing COVID-19 pandemic. Taking part in the survey will entail interacting with an interviewer which could, in theory, increase the likelihood of you contracting the virus. We will take several measures to minimize this risk as much as possible. First, the interviewer will remain at least 2 meters away from you throughout the interview, to facilitate social distancing. Interviews will be conducted outside, if possible. You will be offered disposable masks and gloves before the interview, should you wish to wear them. The tablets we use to collect your responses will be sanitized both before and after each interview. All enumerators will have their temperature checked each day and asked to report on any symptoms they are experiencing. If enumerators feel ill, we will not send them into the field. Finally, our research team will be monitoring the local COVID-19 situation every day, and will not send enumerators into the field if the risks of transmission are deemed unacceptably high. If you do feel ill after completing the survey, you should [insert up-to-date information about who to contact in local health system]. If you feel unwell, you should also contact our survey team on [NAME AND CONTACT NUMBER OF FIELD COORDINATOR] so we can take additional precautions to ensure the safety of the interviewing teams and other participants.

There are no direct benefits to you from participating in the survey. However, the information you would provide will help us design effective education policy in Ghana

Contact information and who to contact in the event of a complaint

You can ask any questions that you have about the study now. If you have a question later that you didn't think of now, you can contact IPA at +233 030-279-0372 and ask for Mubarik Yakubu, the Research Associate, or Salifu Amadu, the Research Manager, with any questions or concerns. You can also call +44 (0)207 291 4800, and ask for the Principal Investigators of this research: Dr. Sonya Krutikova (Depute Research Director, Institute for Fiscal Studies) or Prof. Orazio Attanasio (Professor of Economics, Yale). You may also contact the IPA Institutional Review Board via e-mail - humansubjects@poverty-action.org.

If you have a complaint about any aspect of this study, please contact IPA or principle investigators of the study using the contact details above, or by emailing ghanasurvey@ifs.org.uk.

For peer review only - http://bmjopen.bmj.com/site/about/guidelines.xhtml

You have the right to decline your participation in this survey at any time by simply not completing the survey or emailing ghanasurvey@ifs.org.uk. No data will be submitted without your explicit consent, which we will ask again at the end of the survey.

Data Archiving

At the end of this study all data will be made anonymous and archived for other researchers to use via the UK Data Service and/or the World Bank Microdata Archive.

Agreement to Participate

I certify that the survey has been explained to me, and I have had the opportunity to ask questions. All questions have been answered satisfactorily. I consent voluntarily to participate as a subject in this study and understand that I have the right to withdraw from the study at any time without in any way it affecting me in any way. I consent to the processing of my personal information for the purposes of this research study.

Signature of Investigator: ___________________________________ Date: _______________

Signature/Thumbprint of respondent: __________________________ Date: ________________

Audio consent

During this interview, you may be audio recorded. You and the enumerator will both not know when this recording may happen. The audio recording may only happen if you consent to it and you will not be recorded against your will. This recording will only be used for data quality assurance purposes only. Do you consent to a possible audio recording?

I give permission for audio recording this interview? Yes [ ] No [ ]

I give permission for audio recording the interview with the child? Yes [ ] No [ ]

For peer review only

S.2 Model information sheet – Primary Caregiver:

Evaluation of a pre-school program implemented at scale in Ghana

This study has been approved by the UCL Research Ethics Committee (Project ID Number): 21361/001

PARTICIPANT INFORMATION SHEET

Introduction

This document contains information to help you decide whether you would like to complete the survey. Please take your time to think about the points raised before making your decision about whether or not to participate. If there is anything that is not clear, you can ask me (the surveyor) or my supervisor. Or you can contact the researchers directly by emailing at ghanasurvey@ifs.org.uk. If you wish to raise a complaint, please see contact details below.

What is this research project for?

We are conducting this research to better understand the effectiveness of a preschool program in aiding child development, with researchers at the Institute for Fiscal Studies (UK), Yale (USA), University of Pennylvania (USA) and University of Ghana. Your participation is important to us! By participating in this study, you can help us learn more about how pre-school programs can improve child development, and make children readier to start school.

Why have I been chosen?

Your household has been chosen for this interview as you have a child attending the nearby kindergarten.

What will I be asked to do and what information will we collect?

In this survey we are collecting information on your household, as well as information on your child who has recently been enrolled in pre-school and you as the primary care giver.

The household component of the survey gathers information on demographics, your educational history as well as that of other household members, dwelling, land, livestock, assets, engagement in economic activities.

Afterward a caregiver of a selected child in this household will be interviewed separately for some information about the caregivers' background, child care practice and beliefs, social networks and physical and mental well-being, as well as some information about the development of the children in this community. I will also spend some time with one child from this household who is currently enrolled in kindergarten, to take him/her through some games, as well as measuring their height and weight. I would like to invite you to participate in this survey. Both interviews plus the games with the child combined will last about 1 hour.

Right to Refuse or Withdraw

Participation in this survey is voluntary and you can choose not to participate. If you decide not to participate or withdraw at any point, nothing will happen to you or your family.

You can withdraw your consent from the study at any time, either during this survey or after. No data will be submitted without your explicit consent, which is gathered at the beginning and end of the survey. If you wish in future to withdraw your information from the survey, or to take away your consent, you can do so at any time by contacting us at ghanasurvey@ifs.org.uk. This address will be monitored until the end of 2024.

For peer review only - http://bmjopen.bmj.com/site/about/guidelines.xhtml

You have the right to ask questions and have those questions answered. If you have any question about the study please ask me now. You can also call [NAME AND CONTACT NUMBER OF FIELD COORDINATOR] if you have any question later.

What are the possible benefits of taking part in this research?

There are no direct benefits to you from participating in the survey. However, the information you would provide will help us design effective programs to pre-school education in Ghana.

What are the disadvantages and/or risks of taking part in the study?

There are additional risks presented by the ongoing COVID-19 pandemic. Taking part in the survey will entail interacting with an interviewer which could increase the likelihood of you contracting the virus. We will take several measures to minimize this risk as much as possible. First, the interviewer will remain at least 2 meters away from you throughout the interview, to facilitate social distancing. Interviews will be conducted outside, if possible. You will be offered disposable masks and gloves before the interview, should you wish to wear them. The tablets we use to collect your responses will be sanitized both before and after each interview. All enumerators will have their temperature checked each day and asked to report on any symptoms they are experiencing. If enumerators feel ill, we will not send them to collect any interviews. Finally, our research team will be monitoring the local COVID-19 situation every day, and will not send enumerators to conduct any interviews if the risks of transmission are deemed unacceptably high. If you do feel ill after completing the survey, you should contact [insert up-to-date information about who to contact in local health system] . If you feel unwell, you should also contact our survey team on [NAME AND CONTACT NUMBER OF FIELD COORDINATOR] so we can take additional precautions to ensure the safety of the interviewing teams and other participants.

Privacy Notice

What is the legal basis for collecting this data?

The legal basis for collecting and processing this information in the United Kingdom is "legitimate interests" (Article 6(1)(f)) of the General Data Protection Regulation 2018. As a research organisation, we have an interest in collecting and processing your data in order to understand how pre-school programs improve child development, and the effectiveness of preschool programs when they are implemented at a large scale

How will we keep your information safe? Who will have access to it?

All information we collect is strictly confidential and will only ever be used for academic research purposes. We will not share information with anyone outside the project team, not even with your parents, friends, or any other person in the village. Even when we prepare a report about the program, we will not mention the name of any person or village that participated.

We will exert our upmost effort to ensure that the data you give us remains safe and secure and that only those on the project team will ever have access to it. While we are in Ghana collecting data, your data will be accessed by me, while I enter it into the tablet during our interview, and my supervisor and the field manager, whose role it is to check the quality of the data. While we are collecting data, your data will be stored on the secure servers of the SurveyCTO. Once data has been collected from all participants, we will delete the data from the SurveyCTO server and no-one from the data collection team will retain any access to your data.

At that point, your survey responses will be transferred outside Ghana to the United Kingdom, where it will be stored on secure servers at the Institute for Fiscal Studies. At a later date it may be transferred to and stored securely at the University of Pennsylvania and Yale. Only researchers working on this project will have access to identifiable data. Only de-identified data is used for analysis. The IFS has policies and controls in place that are

For peer review only - http://bmjopen.bmj.com/site/about/guidelines.xhtml

aligned with ISO27001 (an international standard in information management) to ensure that your data is not lost, accidentally destroyed, misused or disclosed, and is not accessed except by members of the research team in the proper performance of their duties. This means that all information is handled according to its classification (for example personal data, publicly available data) and that access to the data is restricted accordingly, both through electronic and physical means. Staff are trained to ensure awareness of the importance of data security and practical knowledge of how to keep data safe. In addition, back-ups of our data are stored offsite in a secure UK location, which is ISO27001 compliant. We will store your contact information separately from any other information we hold on you. We will securely store your contact information in order for us to contact you again in the future for further surveys. At the point where there is no chance of running a further survey, we will delete all identifiable data.

How long will we keep your data?

As we would like to look at long term trends and we are holding your data for research purposes, we have not set a limit on how long we would like to keep the information you give. However, at the point where there is no chance of running a further survey, we will delete all identifiable data.

As according to Economic and Social Research Council guidelines, once the study has concluded, the data (without personal information) will be archived for other researchers to access.

What will happen with the results of this research?

The information that we gather will be analysed by the research team. It will contribute to research reports and academic papers on children's development and pre-school program effectiveness in Ghana. The reports will be available to download for free from the Institute for Fiscal Studies' website (www.ifs.org.uk). The reports will never include any information from which it would be possible to identify you.

Your rights

The UK Data Protection Act (2018) provides the following rights for individuals:

- The right to be informed
- The right of access
- The right of rectification
- The right of erasure
- The right to restrict processing
- The right to data portability
- The right to object
- Rights in relation to automated decision making and profiling

As your data will be processed for scientific research purposes as detailed above, various exceptions from these rights apply. In particular, the right to access, the right to rectification, the right to restrict processing, and the right to object do not apply, provided the appropriate safeguards are in place. In addition, the right to erasure is not absolute, and does not apply if it would seriously impair processing necessary for scientific research.

However, as explained above, if you wish to submit a request for your data to be deleted, please contact us at ghanasurvey@ifs.org.uk and your data will not be used in the study.

Contact us - Who is conducting and funding the study?

The research is conducted by researchers at the Institute for Fiscal Studies, University College London, Yale University, University of Pennsylvania and University of Ghana. We have a lot of experience conducting research on early childhood development.

If you have any questions about the research or wish to raise a complaint, you can contact the research team whose contact information is below. If you wish to raise a complaint please contact the Principal Investigator of

For peer review only - http://bmjopen.bmj.com/site/about/guidelines.xhtml

the research project, Sonya Krutikova who is based at the Institute for Fiscal Studies in London UK, at ghanasurvey@ifs.org.uk. If you feel that your complaint has not been handled to your satisfaction please contact the Chair of the University College London Research Ethics Committee, at ethics@ucl.ac.uk. If you are concerned about how your personal information is being processed, please contact IFS Data Protection Officer in the first instance (DataProtectionOfficer@ifs.org.uk). If you remain unsatisfied, you may wish to contact the UK Information Commissioner's Office (https://ico.org.uk/).

Who to contact in the event of a complaint?

If you have a complaint about any aspect of this study, please contact IPA or principle investigators of the study using the contact details above, or by emailing ghanasurvey@ifs.org.uk.

The current research team and the organisations they currently work for includes the researchers named below. If the research team members change as a result of staffing changes, the team will only ever include bona fide researchers of similar experience level. If the researchers working on the project move to a different organisation where the work on this project can be continued, your data will be shared with that organisation. An up-to-date list of researchers and their organisations can be found here [url for project site once ready] or can be obtained by emailing ghanasurvey@ifs.org.uk.

Principal researcher:

Sonya Krutikova (IFS, sonya_k@ifs.org.uk)

Orazio Attanasio (Yale, orazio.attanasio@yale.edu)

Other researchers:

Britta Augsburg (IFS, britta_a@ifs.org.uk)
Angus Phimister (IFS, angus_p@ifs.org.uk)
E.Nketiah-Amponsah (University of Ghana, enamponsah@ug.edu.gh)
Sharon Wolf (University of Pennsylvania, wolfs@upenn.edu)

The research project is funded the USAID. The project has been approved by UCL Research Ethics Committee (ID number **21361/001**) [ENTER DETAILS OF OTHER APPROVED IRBs HERE].

IFS Data Protection Officer

Zoe Oldfield (email: zoe_o@ifs.org.uk)

For peer review only - http://bmjopen.bmj.com/site/about/guidelines.xhtml

S3. Data Statement

WHO Data set

Primary Registry and Trial Identifying Number	ISRCTN15360698
Date of Registration in Primary Registry	17/12/2021
Secondary Identifying Numbers	AEARCTR-0008500
Source(s) of Monetary or Material Support	USAID and MRC
Primary Sponsor	The Institute for Fiscal Studies
Secondary Sponsor	N/A
Contact for Public Queries	Britta Augsbury: britta_a@ifs.org.uk<br>The Institute for Fiscal Studies<br>Tel: 020 7291 4800<br>7 Ridgmount Street<br>London<br>WC1E 7AE
Contact for Scientific Queries	Dr Sonya Krutikova (sonya_k@ifs.org.uk)<br>The Institute for Fiscal Studies<br>Tel: 020 7291 4800<br>7 Ridgmount Street<br>London<br>WC1E 7AE
Public Title	Evaluating an early childhood care and education program brought to scale in Ghana
Scientific Title	Lively Minds: Improving health and development through play - Evaluation of a comprehensive ECCE programme at scale in Ghana
Countries of Recruitment	Ghana
Health Condition(s) or Problem(s) Studied	Child development
Interventions	Play schemes for pre-school children.
Key Inclusion and Exclusion Criteria	1. Be aged between 3 and 5 years<br>2. Be enrolled or planning to enroll in the sample preschool in the coming term
Study Type	Interventional, open label cluster randomized control trial.
Date of First Enrolment	7/02/2022

For peer review only - http://bmjopen.bmj.com/site/about/guidelines.xhtml

Sample Size	3240 children
Recruitment Status	Incomplete
Primary Outcome	Measured 2 to 3 academic terms (6 - 9 months approximately) after treatment commences:<br>1. Child cognition as measured using the emergent numeracy, emergent literacy and executive functioning tasks in the IDELA as well as items relating to the same domains from the Harvard Laboratory for Development Studies<br>2. Child health, as measured using height/weight for age and height for weight Z score<br>3. Child socio-emotional development, as measured by the externalizing, internalizing and pro-social domains of the Strengths and Difficulty questionnaire and the socio-emotional items in the IDELA
Key Secondary Outcomes	Measured 2 to 3 academic terms (6 - 9 months approximately) after treatment commences:<br>1. Maternal mental health (Kessler-10 and WEMWBS) and knowledge of ECCE<br>2. Teacher knowledge, motivation and teaching quality (measured through classroom observations)<br>3. Parent-child investment, as measured by quality of the home environment (FCI and HOME) and the parent-child relationship (CPRS)<br>4. WASH practices in the home<br>5. Acute Malnutrition measured by MUAC
Ethics Review	Study protocols have been approved by ethics boards at University College London (IRB 21361/001), Yale University (IRB 2000031549) and Ghana Health Service Ethical Review Committee (IRB 028/09/21).
Completion date	September 2025
Summary Results	n/a as trial incomplete
IPD sharing statement	The data collected in the study will be publicly distributed along with critical documents (ie, protocols and questionnaires) following the publication of the primary results from the trials, which is expected to be within 24 months of the final data collection date.

For peer review only - http://bmjopen.bmj.com/site/about/guidelines.xhtml

For peer review only

Reporting checklist for protocol of a clinical trial.

Based on the SPIRIT guidelines.

Instructions to authors

Complete this checklist by entering the page numbers from your manuscript where readers will find each of the items listed below.

Your article may not currently address all the items on the checklist. Please modify your text to include the missing information. If you are certain that an item does not apply, please write "n/a" and provide a short explanation.

Upload your completed checklist as an extra file when you submit to a journal.

In your methods section, say that you used the SPIRITreporting guidelines, and cite them as:

Chan A-W, Tetzlaff JM, Gøtzsche PC, Altman DG, Mann H, Berlin J, Dickersin K, Hróbjartsson A, Schulz KF, Parulekar WR, Krleža-Jerić K, Laupacis A, Moher D. SPIRIT 2013 Explanation and Elaboration: Guidance for protocols of clinical trials. BMJ. 2013;346:e7586

		Reporting Item	Page Number
**Administrative information**			
Title	#1	Descriptive title identifying the study design, population, interventions, and, if applicable, trial acronym	1

For peer review only

Trial registration	#2a	Trial identifier and registry name. If not yet registered, name of intended registry	2
Trial registration: data set	#2b	All items from the World Health Organization Trial Registration Data Set	Supplementary Materials S3.
Protocol version	#3	Date and version identifier	11
Funding	#4	Sources and types of financial, material, and other support	10
Roles and responsibilities: contributorship	#5a	Names, affiliations, and roles of protocol contributors	1, 10
Roles and responsibilities: sponsor contact information	#5b	Name and contact information for the trial sponsor	10
Roles and responsibilities: sponsor and funder	#5c	Role of study sponsor and funders, if any, in study design; collection, management, analysis, and interpretation of data; writing of the report; and the decision to submit the report for publication, including whether they will have ultimate authority over any of these activities	10
Roles and responsibilities: committees	#5d	Composition, roles, and responsibilities of the coordinating centre, steering committee, endpoint adjudication committee, data management team,	n/a, no committees relevant

For peer review only

		and other individuals or groups overseeing the trial, if applicable (see Item 21a for data monitoring committee)	
**Introduction**			
Background and rationale	#6a	Description of research question and justification for undertaking the trial, including summary of relevant studies (published and unpublished) examining benefits and harms for each intervention	2-3
Background and rationale: choice of comparators	#6b	Explanation for choice of comparators	2-3
Objectives	#7	Specific objectives or hypotheses	3
Trial design	#8	Description of trial design including type of trial (eg, parallel group, crossover, factorial, single group), allocation ratio, and framework (eg, superiority, equivalence, non-inferiority, exploratory)	4-5
**Methods: Participants, interventions, and outcomes**			

For peer review only

Study setting	#9	Description of study settings (eg, community clinic, academic hospital) and list of countries where data will be collected. Reference to where list of study sites can be obtained	3-4
Eligibility criteria	#10	Inclusion and exclusion criteria for participants. If applicable, eligibility criteria for study centres and individuals who will perform the interventions (eg, surgeons, psychotherapists)	5-6
Interventions: description	#11a	Interventions for each group with sufficient detail to allow replication, including how and when they will be administered	4
Interventions: modifications	#11b	Criteria for discontinuing or modifying allocated interventions for a given trial participant (eg, drug dose change in response to harms, participant request, or improving / worsening disease)	n/a
Interventions: adherance	#11c	Strategies to improve adherence to intervention protocols, and any procedures for monitoring adherence (eg, drug tablet return; laboratory tests)	n/a, trial is evaluating effect of policy at scale
Interventions: concomitant care	#11d	Relevant concomitant care and interventions that are permitted or prohibited during the trial	n/a
Outcomes	#12	Primary, secondary, and other outcomes, including the specific measurement variable (eg,	6-7

For peer review only

		systolic blood pressure), analysis metric (eg, change from baseline, final value, time to event), method of aggregation (eg, median, proportion), and time point for each outcome. Explanation of the clinical relevance of chosen efficacy and harm outcomes is strongly recommended	
Participant timeline	#13	Time schedule of enrolment, interventions (including any run-ins and washouts), assessments, and visits for participants. A schematic diagram is highly recommended (see Figure)	5
Sample size	#14	Estimated number of participants needed to achieve study objectives and how it was determined, including clinical and statistical assumptions supporting any sample size calculations	5
Recruitment	#15	Strategies for achieving adequate participant enrolment to reach target sample size	5-6

Methods: Assignment of interventions (for controlled trials)

For peer review only

Allocation: sequence generation	#16a	Method of generating the allocation sequence (eg, computer-generated random numbers), and list of any factors for stratification. To reduce predictability of a random sequence, details of any planned restriction (eg, blocking) should be provided in a separate document that is unavailable to those who enrol participants or assign interventions	4-5
Allocation concealment mechanism	#16b	Mechanism of implementing the allocation sequence (eg, central telephone; sequentially numbered, opaque, sealed envelopes), describing any steps to conceal the sequence until interventions are assigned	n/a, open label trial
Allocation: implementation	#16c	Who will generate the allocation sequence, who will enrol participants, and who will assign participants to interventions	5
Blinding (masking)	#17a	Who will be blinded after assignment to interventions (eg, trial participants, care providers, outcome assessors, data analysts), and how	n/a
Blinding (masking): emergency unblinding	#17b	If blinded, circumstances under which unblinding is permissible, and procedure for revealing a participant's allocated intervention during the trial	n/a

For peer review only

Methods: Data collection, management, and analysis

Data collection plan	#18a	Plans for assessment and collection of outcome, baseline, and other trial data, including any related processes to promote data quality (eg, duplicate measurements, training of assessors) and a description of study instruments (eg, questionnaires, laboratory tests) along with their reliability and validity, if known. Reference to where data collection forms can be found, if not in the protocol	7
Data collection plan: retention	#18b	Plans to promote participant retention and complete follow-up, including list of any outcome data to be collected for participants who discontinue or deviate from intervention protocols	7
Data management	#19	Plans for data entry, coding, security, and storage, including any related processes to promote data quality (eg, double data entry; range checks for data values). Reference to where details of data management procedures can be found, if not in the protocol	7

For peer review only

Statistics: outcomes	#20a	Statistical methods for analysing primary and secondary outcomes. Reference to where other details of the statistical analysis plan can be found, if not in the protocol	8-9
Statistics: additional analyses	#20b	Methods for any additional analyses (eg, subgroup and adjusted analyses)	9
Statistics: analysis population and missing data	#20c	Definition of analysis population relating to protocol non-adherence (eg, as randomised analysis), and any statistical methods to handle missing data (eg, multiple imputation)	9
**Methods: Monitoring**			
Data monitoring: formal committee	#21a	Composition of data monitoring committee (DMC); summary of its role and reporting structure; statement of whether it is independent from the sponsor and competing interests; and reference to where further details about its charter can be found, if not in the protocol. Alternatively, an explanation of why a DMC is not needed	n/a
Data monitoring: interim analysis	#21b	Description of any interim analyses and stopping guidelines, including who will have access to these interim results and make the final decision to terminate the trial	n/a

For peer review only

Harms	#22	Plans for collecting, assessing, reporting, and managing solicited and spontaneously reported adverse events and other unintended effects of trial interventions or trial conduct	n/a
Auditing	#23	Frequency and procedures for auditing trial conduct, if any, and whether the process will be independent from investigators and the sponsor	n/a
**Ethics and dissemination**			
Research ethics approval	#24	Plans for seeking research ethics committee / institutional review board (REC / IRB) approval	2
Protocol amendments	#25	Plans for communicating important protocol modifications (eg, changes to eligibility criteria, outcomes, analyses) to relevant parties (eg, investigators, REC / IRBs, trial participants, trial registries, journals, regulators)	9-10
Consent or assent	#26a	Who will obtain informed consent or assent from potential trial participants or authorised surrogates, and how (see Item 32)	6,9
Consent or assent: ancillary studies	#26b	Additional consent provisions for collection and use of participant data and biological specimens in ancillary studies, if applicable	n/a

For peer review only

Confidentiality	#27	How personal information about potential and enrolled participants will be collected, shared, and maintained in order to protect confidentiality before, during, and after the trial	7
Declaration of interests	#28	Financial and other competing interests for principal investigators for the overall trial and each study site	10
Data access	#29	Statement of who will have access to the final trial dataset, and disclosure of contractual agreements that limit such access for investigators	10
Ancillary and post trial care	#30	Provisions, if any, for ancillary and post-trial care, and for compensation to those who suffer harm from trial participation	n/a
Dissemination policy: trial results	#31a	Plans for investigators and sponsor to communicate trial results to participants, healthcare professionals, the public, and other relevant groups (eg, via publication, reporting in results databases, or other data sharing arrangements), including any publication restrictions	9
Dissemination policy: authorship	#31b	Authorship eligibility guidelines and any intended use of professional writers	10

For peer review only

Dissemination policy: reproducible research	#31c	Plans, if any, for granting public access to the full protocol, participant-level dataset, and statistical code	n/a

Appendices

Informed consent materials	#32	Model consent form and other related documentation given to participants and authorised surrogates	Supplementary Materials S1. and S2.
Biological specimens	#33	Plans for collection, laboratory evaluation, and storage of biological specimens for genetic or molecular analysis in the current trial and for future use in ancillary studies, if applicable	n/a

None The SPIRIT Explanation and Elaboration paper is distributed under the terms of the Creative Commons Attribution License CC-BY-NC. This checklist can be completed online using https://www.goodreports.org/, a tool made by the EQUATOR Network in collaboration with Penelope.ai

For peer review only

BMJ Open

Lively Minds: Improving health and development through play – a randomised controlled trial evaluation of a comprehensive ECCE programme at scale in Ghana

Journal:	*BMJ Open*
Manuscript ID	bmjopen-2022-061571.R1
Article Type:	Protocol
Date Submitted by the Author:	22-Jul-2022
Complete List of Authors:	Augsburg, Britta; Institute for Fiscal Studies,<br>Attanasio, Orazio; Yale University<br>Dreibelbis , Robert; London School of Hygiene and Tropical Medicine, Disease Control Department; London School of Hygiene and T<br>Nketiah-Amponsah, Edward; University of Ghana,<br>Phimister, Angus; Institute for Fiscal Studies,<br>Wolf, Sharon; University of Pennsylvania, Graduate School of Education<br>Krutikova, Sonya; Institute for Fiscal Studies
**Primary Subject Heading**:	Evidence based practice
Secondary Subject Heading:	Health services research, Public health, Health policy
Keywords:	PRIMARY CARE, PUBLIC HEALTH, EDUCATION & TRAINING (see Medical Education & Training), Health policy < HEALTH SERVICES ADMINISTRATION & MANAGEMENT

SCHOLARONE™
Manuscripts

BMJ Open Protocol

Lively Minds: Improving health and development through play – a randomised controlled trial evaluation of a comprehensive ECCE programme at scale in Ghana

Authors[a]

For peer review only

1- Institute for Fiscal Studies
2- Yale University
3- London School of Hygiene and Tropical Medicine
4- University of Ghana
5- University of Pennsylvania

Contact Details:
Britta Augsburg (corresponding author): britta_a@ifs.org.uk
Orazio Attanasio: orazio.attansio@yale.edu
Robert Dreibelbis: Robert.Dreibelbis@lshtm.ac.uk
Edward Nketiah-Amponsah: enamponsah@ug.edu.gh
Angus Phimister: angus_p@ifs.org.uk
Sharon Wolf: wolfs@upenn.edu
Sonya Krutikova: sonya_k@ifs.org.uk

Britta Augsburg[1], Orazio Attanasio[1,2], Robert Dreibelbis[3], Edward Nketiah-Amponsah[4], Angus Phimister[1], Sharon Wolf[5], Sonya Krutikova[1].

ABSTRACT

Introduction

Many children in developing countries grow up in environments that lack stimulation, leading to deficiencies in early years' development. Several efficacy trials of early childhood care and education programmes (ECCE) have demonstrated potential to improve child development; evidence on whether these effects can be sustained once programmes are scaled is much more mixed. This study evaluates whether an ECCE programme shown to be effective in an efficacy-trial maintains effectiveness when taken to scale by the Government of Ghana (GoG). The findings will provide critical evidence to the GoG on effectiveness of a programme it is investing in, as well as a blueprint for design and scale up of ECCE programmes in other developing countries, which are expanding their investment in ECCE programmes.

Methods and Analysis

This study is a cluster randomized control trial, in which the order that districts receive the programme is randomized. A minimum sample of 3240 children and 360 schools will be recruited across 72 district-school cohort pairs. The primary outcomes are (i) child cognitive and socio-emotional development measured using the International Development and Early Learning Assessment tool, the Strengths and Difficulties Questionnaire, and tasks from the Harvard Laboratory for Development Studies, (ii) child health (measured using height/weight for age, height-for-weight Z scores). Secondary outcomes include (i) maternal mental health, (using Kessler-10 and Warwick Edinburgh Mental Wellbeing Scale), knowledge of ECCE practices, (ii) teacher knowledge, motivation, teaching quality (measured with classroom observation), (iii) parental investment (using the Family Care Index and Home Observation Measurement of the Environment and the Child Parent Relationship Scale), (iv) WASH practices (v) acute malnutrition (using MUAC). We will estimate unadjusted and adjusted intent-to-treat effects.

Ethics approval

Study protocols have been approved by ethics boards at University College London (21361/001), Yale University (2000031549) and Ghanaian Health Service Ethics Review Committee (028/09/21).

Trial registration number

AEARCTR-0008500, ISRCTN15360698

Keywords

Child health, child development

ARTICLE SUMMARY

Strengths and Limitations of this Study

For peer review only - http://bmjopen.bmj.com/site/about/guidelines.xhtml

- This trial assesses the effect of a programme previously demonstrated to be effective in a small efficacy trial now adopted by government and being brought to scale.
- Scaling up effective interventions has proven challenging in the past; this study will provide much needed evidence on whether an intervention designed to be scalable can be effective.
- A challenge in this study is measuring and adjusting for differences in programme delivery quality across different districts.

For peer review only

For peer review only - http://bmjopen.bmj.com/site/about/guidelines.xhtml

INTRODUCTION

Over 250 million children worldwide under the age of 5 years are at risk of not achieving their developmental potential due to poverty, poor health and nutrition, and deficient care and stimulation[1].The majority of these children live in low- and middle-income countries (LMICs), 1.6 million in Ghana[2]. In Northern Ghana, the setting of this study, and where most families live on less than US$2 per day, 20% of under 5s are stunted and 39% of 3-4 years olds are off-track cognitively[3].

School enrolment alone does not offset these disadvantages: in spite of great global progress in primary school enrolment, around 125 million children lack age-appropriate skills around the world[4]. Years of exposure to poor environments have adverse long-run effects on children's cognitive and socio-emotional development, health and psychological well-being, and earning potential, increasing the risk of poverty, early marriage and parenthood[5]. A recent Lancet review highlights that 'children at elevated risk for compromised development due to stunting and poverty are likely to forgo about a quarter of average adult income per year' [6].

As shown in a substantial body of evidence and increasingly recognized by policymakers, Early Childhood Care and Education (ECCE) interventions offer an opportunity to ameliorate these detrimental effects at a time of heightened developmental sensitivity and malleability[7]. The Government of Ghana (GoG) has implemented some of the most advanced ECCE policies in the Sub-Saharan Africa (National Early Childhood Care and Development Policy in 2004 and 2 years of public pre-primary education in 2007), resulting in universal net enrollment[8]. The GoG, however, recognizes that significant challenges remain: A third of Ghanaian pre-school (KG) children lack the necessary skills to thrive in school, including early academic and behavioural skills, social-emotional development, and aspects of physical health including motor development[9] and deficits persist and grow through primary school, where a fourth of pupils do not meet all proficiency cut-offs[10].

In 2017/18, OA and SK led an efficacy trial of *Lively Minds* - a holistic ECCE programme which engages parents and pre-schools to achieve healthy child development of 3-5 years olds in rural Ghana and promotes inclusion of children with disabilities. The trial revealed a positive impact on child health, as well as cognitive and socio-emotional development, partly mediated by improvements in parenting practices[11].

As a result of this evidence, the Ghana Education Service (GES) plans to integrate the *Lively Minds* programme with core instruction across all pre-schools in Northern Ghana over the next four years aiming to cover over 4,000 pre-schools, reaching 1.3 million children, reducing costs to $7 per child per year (less than a third of the original cost, and significantly below typical group parenting programme costs in LMICs, estimated to be US$30–35 per child[12]). Ownership of the programme has been transferred from the NGO that developed it to GES. We will therefore refer to the programme as the *GES Lively Minds* (GES-LM) programme.

This study is a cluster randomized control trial (CRCT) evaluating this scale-up, with randomization achieved through randomization of the order in which districts begin to receive the program. We will assess whether the impacts found at efficacy trial stage are sustained and investigate key mechanisms underlying these in order to determine how implementation and cost-effectiveness could be further improved.

Objectives

The project's key research questions are as follows:

1. What is the impact of the GES-LM at scale on child development?

For peer review only - http://bmjopen.bmj.com/site/about/guidelines.xhtml

2. How does the impact of the intervention vary by child, teacher, classroom/school and district characteristics?
3. What role do intervention fidelity and compliance play (intensity of training, dosage, hygiene promotion, quality of play sessions, supervision by teachers and district education offices) and how can the design be adapted to maximise cost-effectiveness?
4. Are impacts achieved through changes within the classroom/school and/or the children's home environments, including teacher and parent behaviour?

METHODS AND ANALYSIS

Study Setting

The programme will be rolled out at scale in Northern Ghana, where prevalence of chronic malnutrition is nearly twice as high as the national average (at 33% and 19% respectively) and 60-70% of the population were classified as poor in 2017, compared to 23% nationally[13,14]. Baseline conditions among the sample in the efficacy trial reveal that one third of children (3-5 years) had acute malnutrition and had experienced diarrhoea in the last 30 days; less than a third could identify a shape such as a triangle and even fewer were able to sort figures based on colour and shape. Around half of the mothers were at risk of depression and only 13% reported that *anyone* in the household had played with the target child in the last 3 days. The children live in households of on average ten members in which only 20% ever attended school and even fewer are literate (8%). Household sanitary infrastructure is largely lacking - nearly 80% report that they defecate openly. Access to public infrastructure is dismal: one in three communities do not have access to electricity and more than half cannot be accessed for 1-2 months of the year due to lack of paved roads[15].

Intervention Description

The program developed by the NGO Lively Minds aims to provide pre-school age children with the foundations they need to succeed in school. As young children transition to school, they draw on multiple skills to succeed in the classroom, including early academic and behavioural skills, social-emotional development, and aspects of physical health including motor development[16,17]. Exposure to quality ECCE has the potential to improve school readiness across these various domains. Current conceptualizations of ECCE quality rest however not only on children's experiences in their classroom, but also on their home environments and point to the critical role that warm, nurturing, responsive and stimulating relationships with caregivers both at home and school play. Attachment theory focuses on the importance of consistent and sensitive interactions with teachers and parents[18]; constructivist learning theories focus on the development of cognitive skills through engaging in age-appropriate activities[19]. Further, regular communication between parents and schools allows them to work together toward children's learning and development and has been shown to improve longer-term academic outcomes for preschool and kindergarten children[20,21].

Building on this theoretical framework, the Lively Minds intervention has four core aims:

- To increase children's opportunities for scaffolded, individualized or small group learning experiences at home and at pre-school.
- To reduce harsh and corporal punishment while improving positive behaviour management ensuring that children receive responsive, warm, nurturing caregiving from their caregivers at pre-school and at home.
- To improve hygiene practices and nutritional content in order to improve children's physical health, while considering constraints parents face.

For peer review only - http://bmjopen.bmj.com/site/about/guidelines.xhtml

- To strengthen engagement of mothers with children's schools in order to facilitate a more holistic approach to children's care and learning, as well as empower mothers to have a say in the quality of education their children receive at school.

To this end the intervention consists of three key components:

(1) Play Schemes (PS) for children attending KG (age 3-5): Children learn through play rotating between five play stations set up in KG classrooms, each of which is run by a trained woman from the community (often the mother of one of the children participating). At each play station mothers teach a small group of children (no more than 5) using discovery-based teaching methods through simple games and activities such as puzzles, sorting by shape and size, recognising letters and numbers, reading books and building with blocks. Children who are not in one of the five groups play outdoor games led by one of the trained women while they wait for their turn. Children have to wash their hands with soap before participating in the PS so that they become sensitized to the importance of this practice.

(2) Parenting courses for the women who run the PS: Monthly parenting workshops which aim to increase awareness and understanding of the sensitive caregiving and stimulation that children need to thrive, the adverse effects of harsh punishment and reinforce behaviours encouraged by the program including practices that stimulate children's development and health.

(3) Training and supervision of KG teachers: KG teachers are the *trainers* within this program. They enrol mothers in the community to volunteer to implement the PS, train the mothers on how to do this, oversee implementation of the PS in their classrooms and lead the parenting workshops. In order to do this, KG teachers receive ongoing training on all aspects of the program and supervision visits from the central program team, situated within the Ghana Ministry of Education.

Figure 1 summarises the conceptual framework underpinning this program, highlighting key channels through which the program is expected to impact children's developmental outcomes and school readiness. A critical and distinguishing feature of the program is that through engaging KG teachers and mothers it has the potential to *simultaneously* improve the home and pre-school environments; typically, ECCE programs are designed to target one or the other.

Figure 1. Conceptual framework underpinning the Lively Minds intervention

Figure 1 here

The program is owned and implemented by the Ghana Government Education Service (GES). This is a key difference between the version of the program evaluated in the efficacy trial and the current version. At that time the program was owned by the Lively Minds NGO who engaged the GES in implementation but remained in the driving seat throughout, retaining control over program content and implementation strategy and leading on all aspects of program implementation and monitoring on the ground. Control and responsibility for all of these elements has now been transferred to GES. Lively Minds provides technical assistance; for example, it helps districts set up the necessary monitoring and accounting data-bases, attends key training sessions of district teams and KG teachers, responds to queries from the GES Working Group in charge of the program and accompanied the District officials to some of the monitoring visits.

Implementation is conducted at the district level i.e. once a district is enrolled in the program all of the KGs in the district are invited to participate. Before enrolment of districts begins,

For peer review only - http://bmjopen.bmj.com/site/about/guidelines.xhtml

Lively Minds NGO familiarises the regional GES office[ii] with the Lively Minds Program and trains a team tasked with mobilisation of the districts. Following this there are five main implementation stages starting from enrolment of the district into the program. These are summarised in Figure 2. Supplementary Material S1 provides a more detailed explanation of what each step entails.

Figure 2. GES Lively Minds implementation model

Figure 2 here

Trial Design

This study is an open label cluster randomized control trial, accommodating the phased and complete roll-out of the GES-LM program. The program will be scaled to all of Northern Ghana's 60 districts over 10 phases; each phase (equivalent to one school term) covering a district group (DG) of 6 districts, from September 2021 to September 2024 (Panel A, Figure 3). Researchers at the IFS randomized districts to DGs using R version 4.1.1. DGs will be enrolled into the evaluation study from January 2022 to September 2024 (hence leaving out DG1) in 6 evaluation cohorts (EC) (Panel B, Figure 3). In each EC, one DG is allocated at random into the treatment group and one to the control DG. DG6 and DG7 are used first as control and later as treatment DGs, relying on different cohorts of children (those starting school in 2022 and those in 2023) (in different schools). DG10 will provide the control group for EC5 and EC6 using different schools. Control DGs will begin to receive the programme 3 terms after being enrolled into the study, after endline data collection has been completed (with the exception of EC6, where control will receive the programme after 2 terms). Baseline data collection will start at the end of January 2022 (after participant enrolment) (Panel C, Figure 3). Data collection will proceed continuously through to the end of the project in early 2025. Figure 4 shows this design in a flow diagram, Figure 5 shows the participant timeline.

Figure 3. Project design

Figure 3 here

Figure 4. Randomization flow Diagram

Figure 4 here

Figure 5. Participant Timeline

Figure 5 here

Sample size and Sampling

Within each district, we plan to randomly sample 5 programme-eligible preschools (excluding efficacy trial schools) and a minimum of 9 children who are enrolled or about to enrol in the sampled preschool. In DG6, DG7 and DG10 we will sample children and schools twice (once for each cohort of children). When we sample for a second time, we will exclude schools

[ii] There are currently 16 regions in Ghana made up of 261 districts.

For peer review only - http://bmjopen.bmj.com/site/about/guidelines.xhtml

For peer review only

previously enrolled into the study. This gives us a minimum total sample of 360 preschools and 3460 children and households.

Schools will be selected at random from a complete school list in each district provided by the GES. We will exclude focus on government schools, and exclude atypical schools, specifically only boys' and only girls' schools (more than 99% of government schools are mixed gender), as well as schools with less than 20 enrolled students. Doing so we exclude 20 schools from the sampling frame (20 small ones, one of which has no boys enrolled in KG).

Our study participants will be children, and their households, that attend, or are due to attend KG. These will be identified from school enrolment lists, except for EC1 where due to the COVID-19 pandemic, school openings were different and lists were not readily available. We therefore sampled in this EC only from a census surrounding the school. To be eligible for the study children must fulfil both of the following criteria (the same as in the efficacy trial):

1. Be aged between 3 and 5 years
2. Be enrolled or planning to enrol in the sample preschool in the coming term

Where more than one child is eligible within a single household we will randomly select one child to be a part of the study. Primary care givers will be asked for their consent (and to give consent on behalf of their child) before being enrolled in the study. Model consent form and information sheet for primary caregivers are provided in Supplementary Materials S2. And S3.

Outcomes

In each sampled unit (school) we intend to conduct 4 separate surveys/assessments types, each to be implemented both at baseline and at endline, 2-3 school terms later. Surveys/assessments include: (1) child assessment, (2) Primary caregiver (PCG) Interview, (3) Kindergarten teacher interview and classroom observation, (4) community survey. These surveys will be collected by local well-trained and experienced interviewers, who will not be informed about treatment allocation.

In order to gather evidence on district-level preparedness for the programme (for later heterogeneity analyses), we additionally plan to conduct a survey of the district official in charge of preschool programmes in each district once. We will also collect secondary data available on districts, such as languages spoken, or voter turnout.

We have three sets of primary outcomes:

1. Child cognitive development measured using the International Development and Early Learning Assessment (IDELA) tool[22] and tasks from the Harvard Laboratory for Development Studies, all previously used in Ghana.
2. Child socio-emotional development measured using Strengths and Difficulties Questionnaire (SDQ)[23,24] administered to the primary care giver.
3. Child health measured using height/weight for age and height for weight Z scores, following WHO guidelines.

Our secondary outcomes are as follows:

1. Maternal mental health, measured used the Kessler-10 and WEMWBS and knowledge of ECCE practices;
2. Teacher knowledge, motivation and teaching quality, measured using classroom observation;
3. Parental investment measured through quality in the home environment (Family Care Index and Home Observation Measurement of the Environment) and the quality of the parent-child relationship (CPRS);

For peer review only - http://bmjopen.bmj.com/site/about/guidelines.xhtml

4. WASH practices in the home (e.g. hand washing practices, toilet facilities, open defecation practices);
5. Acute malnutrition measured using middle upper arm circumference. Beyond being of interest in its own right, this is the health outcome used in the efficacy trial[25], which will allow us to compare outcomes across both trials.

In selecting measures for our key outcomes, we prioritised those which have already been validated and used in the study context (for example IDELA[22,26]) or in a context that is similar. Since very little related work has been conducted in our study context, this was not possible for all outcomes that we want to capture. For these outcomes we next identified measures which have been widely used and validated across multiple contexts, for example the Kessler-10 and FCI. We will adapt these measures to the study context following best practice[27]: tools will be reviewed for socio-cultural relevance and linguistic accuracy through consultation with local professionals and modified accordingly; tools will be translated and back-translated into the all of the main languages spoken in the study communities. Full validation of the tools before administration of the baseline is beyond the scope of this study. However, all outcome measurement tools will be piloted on small samples in the study area, feedback on performance will be collected from the surveyors and further adaptations will be made. As part of the training the survey team will be required to achieve inter-rater reliability of at least 0.8 (kappa statistic) on the child assessment and classroom observation tools. Once data are collected we will assess reliability and validity of the outcome measures by examining whether any items have a lot of missing responses, items show sufficient variability, correlations with other variables go in the expected direction, measures exhibit sufficient degree of internal consistency (Cronbach's alpha of at least 0.7). We will also assess dimensionality of the measures using exploratory and confirmatory factor analysis and functioning of individual items using Item Response Theory to generate item characteristics curves. More detailed information on these outcomes is provided in Supplementary Material S4.

Data management protocols and Data Statement

For details on data management please refer to the project's management registration: https://ifs.org.uk/uploads/Research%20registraion%20form.pdf. Data availability is outlined in Supplementary Material S5.

Sample Size and Power

As our design does not deviate in a substantive way from a conventional two-arm cluster randomized control trial design (CRCT), we use standard CRCT methods to assess statistical power. For the power analysis, we consider the district as a cluster, providing us with 54 clusters, of size 45 each – 5 schools per cluster, 9 children per school. We leverage data available from the efficacy trial to better inform how covariates increase our power to detect effects, including expected loss to follow-up, which we set at 6%.

We calculate minimum detectable effects (MDEs) using the following formula[28]:

$$MDE = \left(\frac{\left(t_{\frac{\alpha}{2}} + t_{\beta}\right)^2 \sigma^2 \left(m\rho\left(1 - R_c^2\right) + (1-\rho)\left(1 - R_p^2\right)\right)}{n} \right)^{\frac{1}{2}} \quad (1)$$

For peer review only - http://bmjopen.bmj.com/site/about/guidelines.xhtml

α denotes the significance level, β power, ρ unconditional ICC, t is are the critical values of the t-distribution, σ is the standard deviation of the outcome, n is children per arm, m is children per cluster, R_c^2 is proportion of cluster level variance component explained by covariates and R_p^2 is the individual equivalent. One can interpret the effect of R_c^2 and R_p^2 as altering the "variance inflation factor" of the power calculation – when they equal zero the formula reduces to the standard CRCT one. We set α to 0.05, β to 0.8.

We estimate the intra-cluster correlation (ICC) for each outcome data using data from the efficacy trial (ranging from 0.011 to 0.110). Note we cannot conduct the same exercise for all of our primary outcomes, as some were not included in the original trial. The minimum detectable effects for some of our primary and secondary outcomes measured at the child/household level are shown in Table 1. The power to detect effects at the school level is significantly more limited.

Table 1. Minimum Detectable Effects

Outcome	MDE
Cognition factor score	0.118
Middle Upper Arm Circumference	0.132
Knowledge Infant Development Index (KIDI)	0.125
Family Care Index (FCI)	0.227
Parent-child interaction (PRC)	0.132

Note: Each outcome controls for the its baseline value only.

The MDE for the cognition score will allow us to determine whether the scaled up, government owned and run version of the Lively Minds program is at least as effective as the NGO-run smaller scale version evaluated in the efficacy trial in its key aim of improving cognitive development of pre-school children. However, if the scale up results in a significant reduction in effectiveness, we will not be able to determine whether the program continues to have an impact. It should be noted that efficacy trial impacts were largest for the poorer children in the sample. On average the areas where the scale-up is happening are poorer than those where the efficacy trial took palace so even if there is some loss of effectiveness as the result of the scale-up we should still be able to detect impacts given our power.

Analysis Plan

Intention to Treat

For our primary and secondary outcomes, we will estimate an intent-to-treat effect of the program. Our design is the same as a cluster randomized control trial stratified by evaluation cohort. As such, we follow the standard estimation technique for two-way stratified cluster-randomised control trials, For the outcome variable Y of individual i in district j at date t, we estimate the following:

$$Y_{ijt} = \mu + \gamma_t + \theta\, Treatment_{ijt} + \boldsymbol{\beta}\, \boldsymbol{X_{ijt}} + \varepsilon_{ij}$$

Where γ_t is an evaluation cohort fixed effect, $Treatment_{ijt}$ is binary indicator variable equal to 1 if individual i is in a treatment district at date t, $\boldsymbol{X_{ijt}}$ is a set of controls variables. The treatment effect we identify is θ, and standard errors will be clustered at the school level.

For peer review only - http://bmjopen.bmj.com/site/about/guidelines.xhtml

We will compute the Romano-Wolf stepdown p-values to adjust for multiple hypothesis testing. Hypotheses will be arranged within families of outcomes - i.e. each set of research questions separately.

The set of controls $\boldsymbol{X}_{ijt}$ will be identified using a double-lasso procedure of the covariates we collect as part of our data collection. We will present estimates of the treatment effect both with and without these control variables. In addition to those controls that the double-lasso procedure generates, we will use (at a minimum) the following controls:

- Mother age (quadratically)
- Mother Education attainment
- Tester fixed effects (where relevant)
- Time between baseline and endline measurements
- Child Age
- Child Sex at birth
- Baseline value of the outcome variable of interest

Heterogeneity

We will explore heterogeneity in treatment effect in two distinct ways.

We are first interested in considering treatment heterogeneity by baseline characteristics of the child, their home environment, and features of the environment at the preschool and district-level. Given the large number of relevant dimensions, we will use recent methodological advances[29] to assess heterogeneity across all of these dimensions in a rigorous and data-driven way.

Second, we will assess how study impacts vary by implementation fidelity and compliance in different dimensions. Given intervention fidelity measures are only observed in treatment districts, we will also examine heterogeneity from the data collected as part of the district official survey, focusing on those covariates that are most predictive of high fidelity in treatment districts.

Stopping Rules

Whilst there is always a risk of unintended consequences in all types of trials, in this sort of intervention such a risk is minimal. However, if there is any clear evidence of harm then the study will halt under international ethical guidelines for medical research.

Additional Analysis

This is a large study with many collaborators, and the data gathered will be able to answer more scientific questions than those outlined in this protocol. The study team expects to conduct and publish such additional analyses.

Patient and Public Involvement

Patients or the public were not involved in the design of our research.

ETHICS AND DISSEMINATION

The trial is overseen by independent ethics review boards, which have reviewed the study protocols. Particular consideration will be given to potentially vulnerable people or groups, especially children, and informed consent will be acquired by IPA Staff from all parents of

For peer review only - http://bmjopen.bmj.com/site/about/guidelines.xhtml

participating children before the commencement of any data collection, and that they can stop participating at any time without providing a reason. Model consent forms are available upon request. Any reports of abuse or dangers to children will be reported to relevant local authorities.

Important protocol modifications will be communicated to all relevant parties (e.g. investigators, REC/IRBs, trial participants, trial registries, journals).

Data will be collected, handled, transferred and secured in a manner which aligns with international best practice. The IFS information security management system is ISO27001 compliant.

We will publish findings through (a) top academic journals in economics and developmental science; (b) presentations at conferences in Ghana, US, and Europe; (c) publication of high impact policy briefs for the wider public; (d) leaflets to communicate findings to participating communities; and (e) partner's websites and social media.

Whilst there is always a risk of unintended consequences in all types of trials, in this sort of intervention such a risk is minimal. However, if there is any clear evidence of harm then the study will halt under international ethical guidelines for medical research.

Contributorship Statement

SK, BA, SW, ENA, RD and OA conceptualized the design and methods of the study and revised the manuscript and approved the final manuscript as submitted. AP drafted the initial manuscript, BA revised the manuscript and approved the final manuscript as submitted. All authors provided input and guidance on study design. All authors approved the final manuscript as submitted and agree to be accountable for all aspects of this systematic review

Competing interests

All authors have no competing interests to declare.

Funding

This study is funded by USAID (grant 7200AA21FA00016) and the Medical Research Council (grant MR/V035312/1). This funding source had no role in the design of this study and will not have any role during its execution, analyses, interpretation of the data, or decision to submit results.

Data sharing

Data to be generated within this study will be made available in the UK Data Archive within two years after completing the endline survey. For articles published earlier than that, data and programming files will be uploaded as supplementary information.

Word count: 4,283

For peer review only - http://bmjopen.bmj.com/site/about/guidelines.xhtml

REFERENCES

1 Grantham-McGregor, S, YB Cheung, S Cueto, P Glewwe, L Richter, B Strupp, *Lancet*, 2007;369:60–70.
2 Lu, C, M Black, L Richter, *The Lancet Global Health*, 2016 Dec; 4(12).
3 MICS snapshot report Ghana 2017-18.
4 UNESCO, EFA Global Monitoring Report, 2013/14.
5 Gertler, P, J Heckman, R Pinto, A Zanolini, C Vermeerch, S Walker, SM Chang, S Grantham-McGregor, *Science*. 2014 May 30; 344(6187): 998–1001.
6 Richter, LM, B Daelmans, J Lombardi, J Heymann, FL Boo, JR Behrman, C Lu, JE Lucas, R Perez-Escamilla, T Dua, ZA Bhutta, *The Lancet*. 2017;389(10064):103-18.
7 Shonkoff, JP, DA Phillips, National Academy Press, 2016.
8 Ministry of Education Ghana, 'Education Strategic Plan 2018-2030'.
9 McCoy,DC , ED Peet, M Ezzati, G Danaei, M Black, CR Sudfeld, W Fawzi, G Fink, *PLoS Medicine*, 2016, 13(6).
10 Ministry of Education, Ghana Education Service, 'National Education Assessment' Technical Report 2016.
11 Attanasio, O, B Caeyers, L Cardona-Sosa, S Cattan, S Krutikova, P Leighton (2020). Link.
12 Horton, S, MM Black. (2017), Chapter 24 in: Bundy DAP, ND Silva, S Horton, et al., editors. Child and Adolescent Health and Development. 3rd edition. Washington (DC).
13 USAID, 2018 "Ghana: Nutrition Profile". Link.
14 Ghana Living Standard Survey, 2016/17.
15 Pisani, L, I Borisova, AJ Dowd (2018), *International Journal of Educational Research*, 91: 1–15. https://doi.org/10.1016/j.ijer.2018.06.007
16 *Snow*, C.E., *Van Hemel*, S.B. The National Academies Press, *2008.*
17 UNESCO (2013). UNESCO-UIS/Brookings Institution. Link.
18 Ainsworth, MDS (1989). *American Psychologist*, 44, 709-716. http://dx.doi.org/10.1037/0003-066X.44.4.709.
19 Gopnik, A, AN Meltzoff, and PK Kuhl. (1999). *The Scientist in the Crib: Minds, Brains, and How Children Learn.* New York, NY: William Morrow & Co.
20 Miedel, WT and AJ Reynolds. (2000). *Journal of School Psychology, 37,* 379–402.
21 Connor, CM, SH Son, AH Hindman, FJ Morrison. (2005). *Journal of School Psychology, 43*(4), 343–375.
22 Pisani, L, I Borisova, AJ Dowd. (2015). Washington, DC. Link.
23 Hoosen, N, EL Davids, PJ De Vries, M Shung-King. (2018). *Child and Adolescent Psychiatry and Mental Health* 12: 6.
24 Goodman, R., *Journal of Child Psychology and Psychiatry*, Jul 1997;38(5):581-6.
25 Amadu, S, O Attanasio, B Caeyers, S Cattan, L Cardona Sosa, S Krutikova, P Leighton, 2018, IFS Report 12996. Link.
26 Wolf, S, M Peele, *Journal of the British Academy*, 8(s2), 71–102
27 Fernald, LCH, E Prado, P Kariger, A Raikes (2017). Washington DC. The World Bank. Link.
28 McConnell, B, M Vera-Hernandez, 2015, IFS Working Paper W15/17, Link.
29 Wager, S, S Athey, *J of the American Statistical Association*, 2018: 113:523, 1228-1242.

Date and Version Number

20/07/2022, version 2

For peer review only - http://bmjopen.bmj.com/site/about/guidelines.xhtml

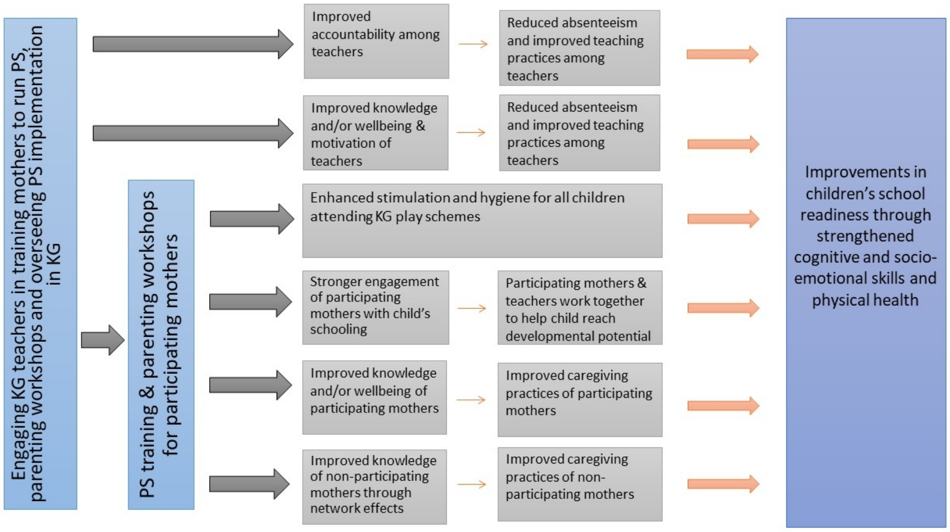

Figure 1. Conceptual framework underpinning the Lively Minds intervention

338x190mm (96 x 96 DPI)

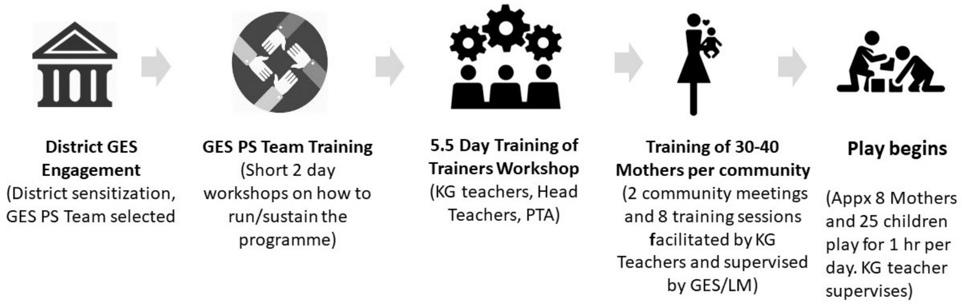

Figure 2. GES Lively Minds implementation model

329x109mm (96 x 96 DPI)

				School Term:	Sep-21	Jan-22	May-22	Sep-22	Jan-23	May-23	Sep-23	Jan-24	May-24	Sep-24	Jan-25
**Panel A**: Intervention roll-out 1=intervention implemented 0=no intervention															
6 districts	DG1				1	1	1	1	1	1	1	1	1	1	1
6 districts	DG2				0	1	1	1	1	1	1	1	1	1	1
6 districts	DG3				0	0	1	1	1	1	1	1	1	1	1
6 districts	DG4				0	0	0	1	1	1	1	1	1	1	1
6 districts	DG5				0	0	0	0	1	1	1	1	1	1	1
6 districts	DG6				0	0	0	0	0	1	1	1	1	1	1
6 districts	DG7				0	0	0	0	0	0	1	1	1	1	1
6 districts	DG8				0	0	0	0	0	0	0	1	1	1	1
6 districts	DG9				0	0	0	0	0	0	0	0	1	1	1
6 districts	DG10				0	0	0	0	0	0	0	0	0	1	1
**Panel B**: Treatment and control allocation & cohort selection															
6 districts	DG1				1	1	1	1	1	1	1	1	1	1	1
6 districts	DG2	EC1	Treatment	Cohort 2022	0	1	1	1	1	1	1	1	1	1	1
6 districts	DG6		Control	Cohort 2022	0	0	0	0	0	1	1	1	1	1	1
6 districts	DG3	EC2	Treatment	Cohort 2022	0	0	1	1	1	1	1	1	1	1	1
6 districts	DG7		Control	Cohort 2022	0	0	0	0	0	0	1	1	1	1	1
6 districts	DG4	EC3	Treatment	Cohort 2022	0	0	0	1	1	1	1	1	1	1	1
6 districts	DG8		Control	Cohort 2022	0	0	0	0	0	0	0	1	1	1	1
6 districts	DG5	EC4	Treatment	Cohort 2023	0	0	0	0	1	1	1	1	1	1	1
6 districts	DG9		Control	Cohort 2023	0	0	0	0	0	0	0	0	1	1	1
6 districts	DG6	EC5	Treatment	Cohort 2023	0	0	0	0	0	1	1	1	1	1	1
6 districts	DG10		Control	Cohort 2023	0	0	0	0	0	0	0	0	0	1	1
6 districts	DG7	EC6	Treatment	Cohort 2023	0	0	0	0	0	0	1	1	1	1	1
6 districts	DG10		Control	Cohort 2023	0	0	0	0	0	0	0	0	0	1	1
**Panel C**: Baseline and endline data collection timing															
6 districts	DG1				1	1	1	1	1	1	1	1	1	1	1
6 districts	DG2	EC1	Treatment	Cohort 2022	0	1	1	1	1	1	1	1	1	1	1
6 districts	DG6		Control	Cohort 2022	0	0	0	0	0	1	1	1	1	1	1
6 districts	DG3	EC2	Treatment	Cohort 2022	0	0	1	1	1	1	1	1	1	1	1
6 districts	DG7		Control	Cohort 2022	0	0	0	0	0	0	1	1	1	1	1
6 districts	DG4	EC3	Treatment	Cohort 2022	0	0	0	1	1	1	1	1	1	1	1
6 districts	DG8		Control	Cohort 2022	0	0	0	0	0	0	0	1	1	1	1
6 districts	DG5	EC4	Treatment	Cohort 2023	0	0	0	0	1	1	1	1	1	1	1
6 districts	DG9		Control	Cohort 2023	0	0	0	0	0	0	0	0	1	1	1
6 districts	DG6	EC5	Treatment	Cohort 2023	0	0	0	0	0	1	1	1	1	1	1
6 districts	DG10		Control	Cohort 2023	0	0	0	0	0	0	0	0	0	1	1
6 districts	DG7	EC6	Treatment	Cohort 2023	0	0	0	0	0	0	1	1	1	1	1
6 districts	DG10		Control	Cohort 2023	0	0	0	0	0	0	0	0	0	1	1

Figure 3. Project design

165x126mm (150 x 150 DPI)

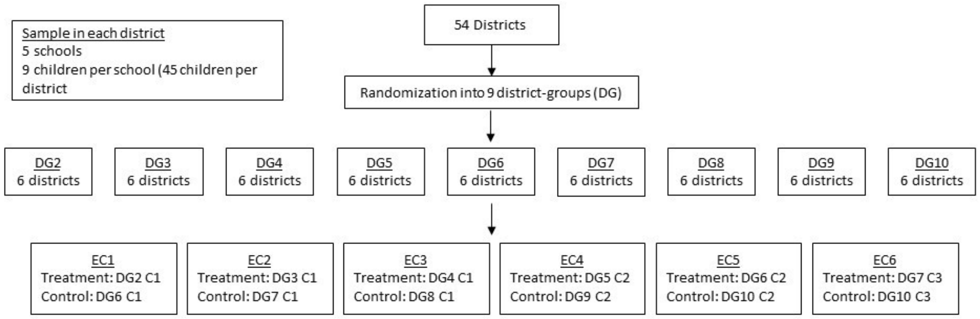

Figure 4. Randomization flow Diagram

252x85mm (96 x 96 DPI)

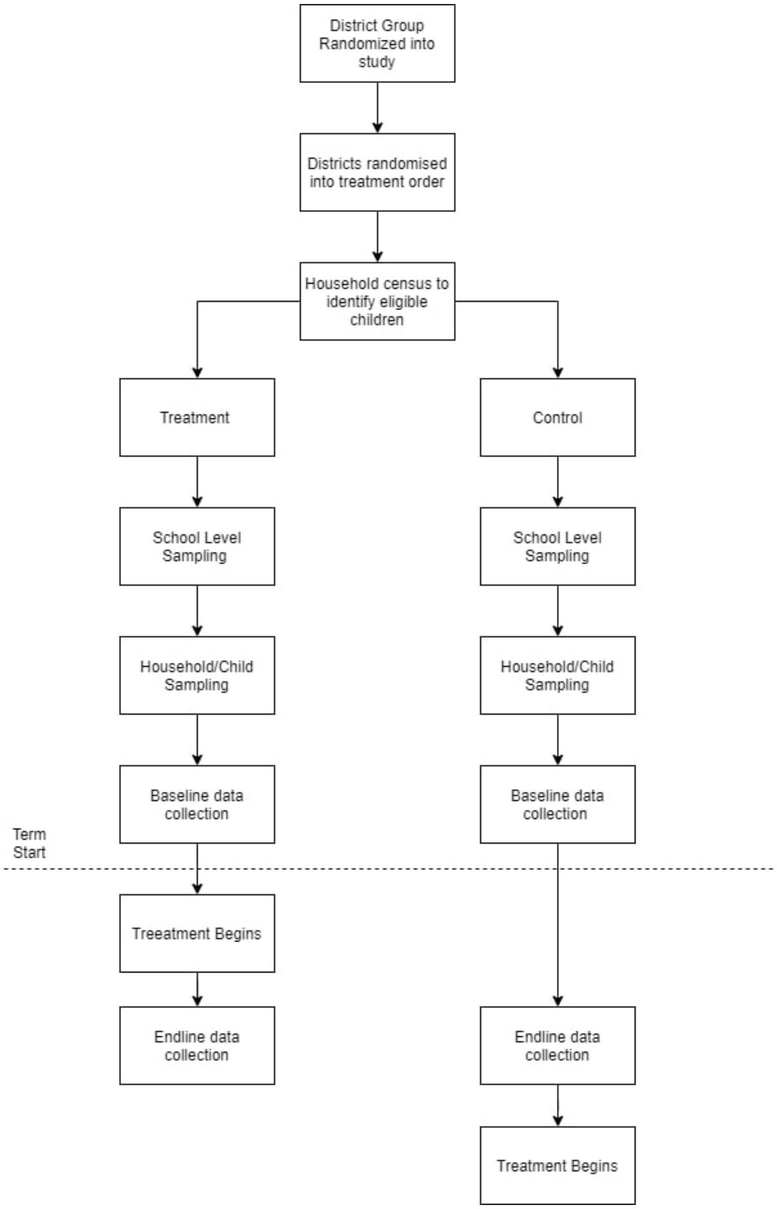

Figure 5. Participant Timeline

212x328mm (72 x 72 DPI)

Supplementary materials

This appendix has been provided by the authors to give readers additional information about the study.

Supplement to: "*Lively Minds: Improving health and development through play – a randomised controlled trial evaluation of a comprehensive ECCE programme at scale in Ghana*"

For peer review only

For peer review only - http://bmjopen.bmj.com/site/about/guidelines.xhtml

S1. GES Lively Minds implementation model:

This supplementary material provides additional information to the intervention description.

1. Regional GES team, with support from Lively Minds NGO, conducts sensitisation of District level GES officials, introducing the program to them, explaining its importance and securing their commitment to implementing it. The District GES appoints a "Play Scheme" (PS) team consisting of District GES staff tasked with leading implementation of the program in all public KGs in the district.
2. Regional GES team conduct 2-day workshop with the District level GES PS team on how to run and sustain the programme.
3. The District GES PS team conducts sensitisation with schools in the district, which identify KG teachers that meet participation criteria to receive training of trainers[i]. These KG teachers along with the Head Teachers from the schools in which they teach attend a 5.5-day Training of Trainers workshop in which they are introduced to the GES Lively Minds programme, how to mobilise mothers to participate in the program, how to train the mothers and how to run the program in their KGs.
4. KG teachers conduct 2 community meetings to introduce the programme and ask for women in the community to volunteer to implement the PS at the KG during school time[ii]. The women who express interest are trained by the KG teachers over the course of 8 sessions under the supervision of the District PS team and Lively Minds NGO representative.
5. Play Schemes begin in KGs – the trained mothers run the Play Schemes in the KGs at least 4 times per week in 2-hour sessions, supervised by the KG teacher. These mothers also attend a monthly parenting workshop led by the KG teacher.

There is ongoing supervision of the KG teachers and Play Scheme implementation within KGs as well as of the parenting courses by the District PS team. This is done through regular unannounced visits by District GES PS team members who complete monitoring forms to record their findings during the visit. District GES PS Team delivers top-up training for the KG teachers and Head Teachers of participating schools twice per term.

[i] The school must have 2 KG teachers in place who are on permanent contracts with GES and speak the local language fluently.
[ii] There is no requirement that the participants are mothers, though most of those who sign up have young children.

For peer review only - http://bmjopen.bmj.com/site/about/guidelines.xhtml

S2. Model consent form – Primary Caregiver:

Consent Form for Primary Caregivers (Primary Caregiver Survey)
INNOVATIONS FOR POVERTY ACTION
Lively Minds Evaluation Extension

Consent Form for Primary Caregivers (Primary Caregiver Survey)

Study Title: Evaluation of a Pre-school Program Implemented at Scale in Ghana (Protocol ID NO: GHC-ERC Number: 028/09/21)

Good day, my name is ___________________________I work for Innovations for Poverty Action (IPA), a research and policy non-profit organization that discovers and promotes effective solutions to global poverty problems.

Purpose of the study

IPA is working with researchers at the Institute for Fiscal Studies (UK), Yale University (USA), University of Pennsylvania (USA) and the University of Ghana to learn more about young children and about how we can improve children's learning outcomes. This study is paid for the by United States Agency for International Development, and the Medical Research Council of the United Kingdom.

We have received official permission from the Ghana Education Service to conduct this survey. We are inviting you and your children to participate in this study.

Procedures

If you choose to participate, you will be asked to complete a survey about your background and household information, caregiver's school engagement activities, household assets, household economic activities, agricultural activities and food insecurity, household savings and credit, household economic shocks and risks, caregiver and children time use, caregiver social capital, school choice and beliefs about quality education, water, sanitation, and hygiene practices, parent-child relationship, caregiver cognitive skills, mental health and wellbeing, caregiver empowerment, children behaviours, and caregiver volunteering activities. This survey will take about 1 hour and 30 minutes of your time.

If you agree that your children participate in this research, they will be asked to participate in an assessment on numeracy, literacy, and social-emotional skills at their school. If you agree that your children participate in this research, they will be asked to participate in an assessment on numeracy, literacy, and social-emotional skills at their school through play-based tasks and take basic anthropometric measurements (i.e. height, weight, arm circumference) - always in the presence of the child's teacher or adult in a similar role. Enumerators will also observe teaching and take inventory of classroom practices. As part of this, we will record a short video of a class while it is in session.

You and your child may be contacted to participate in a follow-up survey or another study at a future date, but you are free to decline participation in the follow-up survey or another study if you wish.

We hope to record a portion of this interview for quality control purposes. This is voluntary, and you are free to decline if you do not wish to be recorded. All video and audio recordings will be destroyed after five years.

We wish to record the GPS coordinates of this interview for tracking purposes. This does not pose any additional risk to you and is voluntary, so, you are free to decline if you feel uncomfortable.

Confidentiality

For peer review only - http://bmjopen.bmj.com/site/about/guidelines.xhtml

The responses you and your children provide will be kept confidential to the extent possible and will only be accessible to the research team and individuals from IPA who oversees the research. Only data or information that does not identify your teachers and pupils in any way may be (a) used for research and academic publications or (b) shared with other people or organizations. It will not be possible to know that a given answer has been provided by your or your children.

While we will take all precautions to prevent this, in the event that there is an outside breach of confidentiality of the data, you will be contacted and informed of the event and the steps being taken to recover your information.

The legal basis for collecting and processing this information in the United Kingdom is "legitimate interests" (Article 6(1)(f)) of the General Data Protection Regulation 2018 and in Ghana is Data Protection Act, 2012 (ACT 843)

Voluntary Participation

Participation is completely voluntary. You and your children are free to (a) decline to participate; (b) withdraw from the study at any time, and (c) skip any question they feel uncomfortable answering without penalty or loss of any existing benefits for doing so.

Risks and Benefits

There are no anticipated risks from taking part in this survey. However, there may be inconvenience and loss of time in participating in this study. All data collection activities will be conducted in strict adherence to the covid-19 protocols including wearing nose masks to reduce the likelihood of either party contracting the virus. There are no direct benefits to you and your children from participating in the survey. However, the information you would provide will help us design an effective education policy in Ghana.

There are no direct benefits to you from participating in the survey. However, the information you would provide will help us design an effective education policy in Ghana.

Contact Information

If you have questions about the research study, you can contact IPA at +233 050 251 4712 and ask for Murphy Edro, the Research Associate. You can also contact the lead researchers directly by emailing them at ghanasurvey@ifs.org.uk.If you have any questions about your rights as a research participant, or complaints, contact Ghana Health Service Ethics Committee at 0503539896 (ask for Nana Abena Apatu) or by emailing them at ethics.research@ghsmail.org. You can also contact UCL Ethics by emailing ethics@ucl.ac.uk.

Do you have any questions that I can answer?

PARTICIPANTS' STATEMENT

I acknowledge that I have read or have had the purpose and contents of the Participant's Consent and Information Sheet read and all questions satisfactorily explained to me in a language I understand (____). I fully understand the contents and any potential implications as well as my right to change my mind (i.e., withdraw from the research) at any point. I understand that if I withdraw before January 2025, I can ask for my data not to be used.

For participation in this research:

1. |___| I voluntarily agree to be part of this research.
2. |___| I voluntarily agree for my children only to be part of this research
3. |___| I voluntarily agree for me and my children to be part of this research
4. |___| I don't agree with any of us being part of this research.

For peer review only - http://bmjopen.bmj.com/site/about/guidelines.xhtml

For audio recording:

1. |__| I voluntarily agree to the audio recording for me only
2. |___| I voluntarily agree to the audio recording for my children only
3. |___| I voluntarily agree to the audio recording for me and my children
4. |___| I don't agree with the audio recording for any of us

For GPS records, I voluntarily agree to the taking of GPS records for my dwelling. |__| Yes |__|No

Name of Participant____

Participant's Signature_________________________________ OR Thumb
Print_____________________________

Date_______________________________

INTERPRETERS' STATEMENT

I interpreted the purpose and contents of the Participant's Consent and Information Sheet to the afore named participant to the best of my ability in the (_______________________________) language to his proper understanding. All questions, appropriate clarifications sort by the participant and answers were also duly interpreted to his/her satisfaction.

Name of Interpreter____

Interpreter's Signature_________________________________ OR Thumb
Print________________

Date_______________________________

STATEMENT OF WITNESS

I was present when the purpose and contents of the Participant Consent and Information Sheet was read and explained satisfactorily to the participant in the (_______________________________) language, which s/he understood. I confirm that s/he was given the opportunity to ask questions/seek clarifications and same were duly answered to his/her satisfaction before voluntarily agreeing to be part of the research.

Name of Witness____

Witness' Signature_________________________________ OR Thumb

Print_______________________

Date_______________________________

INTERVIEWER STATEMENT AND SIGNATURE

I certify that the participant has been given ample time to read and learn about the study. All questions and clarifications raised by the participant have been addressed.

Interviewer's name____

Signature____

Date_______________________________

For peer review only - http://bmjopen.bmj.com/site/about/guidelines.xhtml

S3. Model information sheet – Primary Caregiver:

Evaluation of a pre-school program implemented at scale in Ghana

This study has been approved by the UCL Research Ethics Committee (Project ID Number): 21361/001

PARTICIPANT INFORMATION SHEET

Introduction

This document contains information to help you decide whether you would like to complete the survey. Please take your time to think about the points raised before making your decision about whether or not to participate. If there is anything that is not clear, you can ask me (the surveyor) or my supervisor. Or you can contact the researchers directly by emailing at ghanasurvey@ifs.org.uk. If you wish to raise a complaint, please see contact details below.

What is this research project for?

We are conducting this research to better understand the effectiveness of a preschool program in aiding child development, with researchers at the Institute for Fiscal Studies (UK), Yale (USA), University of Pennylvania (USA) and University of Ghana. Your participation is important to us! By participating in this study, you can help us learn more about how pre-school programs can improve child development, and make children readier to start school.

Why have I been chosen?

Your household has been chosen for this interview as you have a child attending the nearby kindergarten.

What will I be asked to do and what information will we collect?

In this survey we are collecting information on your household, as well as information on your child who has recently been enrolled in pre-school and you as the primary care giver.

The household component of the survey gathers information on demographics, your educational history as well as that of other household members, dwelling, land, livestock, assets, engagement in economic activities.

Afterward a caregiver of a selected child in this household will be interviewed separately for some information about the caregivers' background, child care practice and beliefs, social networks and physical and mental well-being, as well as some information about the development of the children in this community. I will also spend some time with one child from this household who is currently enrolled in kindergarten, to take him/her through some games, as well as measuring their height and weight. I would like to invite you to participate in this survey. Both interviews plus the games with the child combined will last about 1 hour.

Right to Refuse or Withdraw

Participation in this survey is voluntary and you can choose not to participate. If you decide not to participate or withdraw at any point, nothing will happen to you or your family.

You can withdraw your consent from the study at any time, either during this survey or after. No data will be submitted without your explicit consent, which is gathered at the beginning and end of the survey. If you wish in future to withdraw your information from the survey, or to take away your consent, you can do so at any time by contacting us at ghanasurvey@ifs.org.uk. This address will be monitored until the end of 2024.

For peer review only - http://bmjopen.bmj.com/site/about/guidelines.xhtml

You have the right to ask questions and have those questions answered. If you have any question about the study please ask me now. You can also call [NAME AND CONTACT NUMBER OF FIELD COORDINATOR] if you have any question later.

What are the possible benefits of taking part in this research?

There are no direct benefits to you from participating in the survey. However, the information you would provide will help us design effective programs to pre-school education in Ghana.

What are the disadvantages and/or risks of taking part in the study?

There are additional risks presented by the ongoing COVID-19 pandemic. Taking part in the survey will entail interacting with an interviewer which could increase the likelihood of you contracting the virus. We will take several measures to minimize this risk as much as possible. First, the interviewer will remain at least 2 meters away from you throughout the interview, to facilitate social distancing. Interviews will be conducted outside, if possible. You will be offered disposable masks and gloves before the interview, should you wish to wear them. The tablets we use to collect your responses will be sanitized both before and after each interview. All enumerators will have their temperature checked each day and asked to report on any symptoms they are experiencing. If enumerators feel ill, we will not send them to collect any interviews. Finally, our research team will be monitoring the local COVID-19 situation every day, and will not send enumerators to conduct any interviews if the risks of transmission are deemed unacceptably high. If you do feel ill after completing the survey, you should contact [insert up-to-date information about who to contact in local health system] . If you feel unwell, you should also contact our survey team on [NAME AND CONTACT NUMBER OF FIELD COORDINATOR] so we can take additional precautions to ensure the safety of the interviewing teams and other participants.

Privacy Notice

What is the legal basis for collecting this data?

The legal basis for collecting and processing this information in the United Kingdom is "legitimate interests" (Article 6(1)(f)) of the General Data Protection Regulation 2018. As a research organisation, we have an interest in collecting and processing your data in order to understand how pre-school programs improve child development, and the effectiveness of preschool programs when they are implemented at a large scale

How will we keep your information safe? Who will have access to it?

All information we collect is strictly confidential and will only ever be used for academic research purposes. We will not share information with anyone outside the project team, not even with your parents, friends, or any other person in the village. Even when we prepare a report about the program, we will not mention the name of any person or village that participated.

We will exert our upmost effort to ensure that the data you give us remains safe and secure and that only those on the project team will ever have access to it. While we are in Ghana collecting data, your data will be accessed by me, while I enter it into the tablet during our interview, and my supervisor and the field manager, whose role it is to check the quality of the data. While we are collecting data, your data will be stored on the secure servers of the SurveyCTO. Once data has been collected from all participants, we will delete the data from the SurveyCTO server and no-one from the data collection team will retain any access to your data.

At that point, your survey responses will be transferred outside Ghana to the United Kingdom, where it will be stored on secure servers at the Institute for Fiscal Studies. At a later date it may be transferred to and stored securely at the University of Pennsylvania and Yale. Only researchers working on this project will have access to identifiable data. Only de-identified data is used for analysis. The IFS has policies and controls in place that are

For peer review only - http://bmjopen.bmj.com/site/about/guidelines.xhtml

aligned with ISO27001 (an international standard in information management) to ensure that your data is not lost, accidentally destroyed, misused or disclosed, and is not accessed except by members of the research team in the proper performance of their duties. This means that all information is handled according to its classification (for example personal data, publicly available data) and that access to the data is restricted accordingly, both through electronic and physical means. Staff are trained to ensure awareness of the importance of data security and practical knowledge of how to keep data safe. In addition, back-ups of our data are stored offsite in a secure UK location, which is ISO27001 compliant. We will store your contact information separately from any other information we hold on you. We will securely store your contact information in order for us to contact you again in the future for further surveys. At the point where there is no chance of running a further survey, we will delete all identifiable data.

How long will we keep your data?

As we would like to look at long term trends and we are holding your data for research purposes, we have not set a limit on how long we would like to keep the information you give. However, at the point where there is no chance of running a further survey, we will delete all identifiable data.

As according to Economic and Social Research Council guidelines, once the study has concluded, the data (without personal information) will be archived for other researchers to access.

What will happen with the results of this research?

The information that we gather will be analysed by the research team. It will contribute to research reports and academic papers on children's development and pre-school program effectiveness in Ghana. The reports will be available to download for free from the Institute for Fiscal Studies' website (www.ifs.org.uk). The reports will never include any information from which it would be possible to identify you.

Your rights

The UK Data Protection Act (2018) provides the following rights for individuals:

- The right to be informed
- The right of access
- The right of rectification
- The right of erasure
- The right to restrict processing
- The right to data portability
- The right to object
- Rights in relation to automated decision making and profiling

As your data will be processed for scientific research purposes as detailed above, various exceptions from these rights apply. In particular, the right to access, the right to rectification, the right to restrict processing, and the right to object do not apply, provided the appropriate safeguards are in place. In addition, the right to erasure is not absolute, and does not apply if it would seriously impair processing necessary for scientific research.

However, as explained above, if you wish to submit a request for your data to be deleted, please contact us at ghanasurvey@ifs.org.uk and your data will not be used in the study.

Contact us - Who is conducting and funding the study?

The research is conducted by researchers at the Institute for Fiscal Studies, University College London, Yale University, University of Pennsylvania and University of Ghana. We have a lot of experience conducting research on early childhood development.

If you have any questions about the research or wish to raise a complaint, you can contact the research team whose contact information is below. If you wish to raise a complaint please contact the Principal Investigator of

For peer review only - http://bmjopen.bmj.com/site/about/guidelines.xhtml

the research project, Sonya Krutikova who is based at the Institute for Fiscal Studies in London UK, at ghanasurvey@ifs.org.uk. If you feel that your complaint has not been handled to your satisfaction please contact the Chair of the University College London Research Ethics Committee, at ethics@ucl.ac.uk. If you are concerned about how your personal information is being processed, please contact IFS Data Protection Officer in the first instance (DataProtectionOfficer@ifs.org.uk). If you remain unsatisfied, you may wish to contact the UK Information Commissioner's Office (https://ico.org.uk/).

Who to contact in the event of a complaint?

If you have a complaint about any aspect of this study, please contact IPA or principle investigators of the study using the contact details above, or by emailing ghanasurvey@ifs.org.uk.

The current research team and the organisations they currently work for includes the researchers named below. If the research team members change as a result of staffing changes, the team will only ever include bona fide researchers of similar experience level. If the researchers working on the project move to a different organisation where the work on this project can be continued, your data will be shared with that organisation. An up-to-date list of researchers and their organisations can be found here [url for project site once ready] or can be obtained by emailing ghanasurvey@ifs.org.uk.

Principal researcher:

Sonya Krutikova (IFS, sonya_k@ifs.org.uk)

Orazio Attanasio (Yale, orazio.attanasio@yale.edu)

Other researchers:

Britta Augsburg (IFS, britta_a@ifs.org.uk)
Angus Phimister (IFS, angus_p@ifs.org.uk)
E.Nketiah-Amponsah (University of Ghana, enamponsah@ug.edu.gh)
Sharon Wolf (University of Pennsylvania, wolfs@upenn.edu)

The research project is funded the USAID. The project has been approved by UCL Research Ethics Committee (ID number **21361/001**) [ENTER DETAILS OF OTHER APPROVED IRBs HERE].

IFS Data Protection Officer

Zoe Oldfield (email: zoe_o@ifs.org.uk)

For peer review only - http://bmjopen.bmj.com/site/about/guidelines.xhtml

S4. Information on study primary and secondary outcomes

Figure S4.1 provides additional details on primary and secondary outcomes of the study.

Figure S4.1. Study primary and secondary outcome information

For peer review only

For peer review only - http://bmjopen.bmj.com/site/about/guidelines.xhtml

Domain	Instruments	Scoring and other information
**Primary outcomes**		
**Child cognitive development**	International Development and Early Learning Assessment (IDELA) tool	• Score following official guidelines provided by IDELA<br>• Score using Item Response Theory (IRT) for each of the developmental domains measured by IDELA<br>• Construct one overall score using IRT, pooling all IDELA items
	Tasks from the Harvard Laboratory for Development Studies	• Score using IRT for each of the domains measured<br>• Pool all items and score using IRT<br>• Pool with IDELA items and create domain specific and pooled scores using IRT
**Child socio-emotional development**	Strengths and Difficulties Questionnaire (SDQ) administered to the primary care giver.	• Score following official guidelines provided by the tool developers<br>• Create sub-domain scores using IRT<br>• Create overall score using IRT
**Child health**	Height<br>Weight	• Height for age and Weight for age<br>• Height for weight z-score
**Secondary Outcomes**		
**Maternal mental health and knowledge of ECCE**		
Caregiver mental health	Kessler-10	• Construct a total raw score following tool developer's guidance<br>• Construct a score using IRT
	Warwick-Edinburgh Mental Wellbeing Scale (WEMWBS)	• Construct a total raw score following tool developer's guidance<br>• Construct a score using IRT
Caregiver knowledge of ECCE practices	Sub-set of items from the Knowledge of Infant Development Inventory (KIDI)	• Raw score for number correct items<br>• Factor score constructed using IRT
**Parent-child investment**		
Home Environment	Family Care Indicators Questionnaire (FCI) and the Home Observation for Measurement of the Environment (HOME)	• Construct a total raw score following tool developer's guidance<br>• Construct a score using IRT
Caregiver-child relationship	Child Parent Relationship Scale (CPRS)	• Construct a total raw score following tool developer's guidance<br>• Construct a score using IRT
Caregiver instructional practices	Observation checklist; caregiver report	• Factor scores constructed using IRT: separate factor scores for observed and caregiver report items and then overall factor combining observed and caregiver report items.
**Teacher knowledge, motivation and teaching quality**		
Classroom quality	Classroom observation checklist	• Raw score of nr of conditions fulfilled out of a list of 11 (e.g. there are books in the classroom, adequate lighting, backboard etc.)<br>• Factor score constructed using IRT
Teacher practices	Classroom observation checklist	• Separate raw scores and a combined raw score for number of practices observed out of a list of 15 relating to behavior management and instructional practice.<br>• Factor scores for each of the above constructed using IRT
Teacher burn-out	Maslach burn-out battery	• Construct a total raw score following tool developer's guidance<br>• Construct a score using IRT
Teacher mental health	Kessler-10	• Construct a total raw score following tool developer's guidance<br>• Construct a score using IRT
Teacher well-being	Warwick-Edinburgh Mental Wellbeing Scale (WEMWBS)	• Construct a total raw score following tool developer's guidance<br>• Construct a score using IRT
**WASH practices in the home**	Caregiver report (including list randomization for sensitive behaviour) and interviewer observation	• Availability of WASH infrastructure: toilet, handwashing facility (with soap)<br>• WASH practices:<br>○ Handwashing of the respondents and their family members, particularly the target child.<br>○ Sanitation behavior (such as open defecation) of family members
**Child health**	Mid-Upper Arm Circumferences (MUAC)	• Acute Malnutrition measured by Mid-Upper Arm Circumferences (MUAC), using cut-offs as recommended by WHO.

For peer review only

S5. Data Statement

WHO Data set

Primary Registry and Trial Identifying Number	ISRCTN15360698
Date of Registration in Primary Registry	17/12/2021
Secondary Identifying Numbers	AEARCTR-0008500
Source(s) of Monetary or Material Support	USAID and MRC
Primary Sponsor	The Institute for Fiscal Studies
Secondary Sponsor	N/A
Contact for Public Queries	Britta Augsbury: britta_a@ifs.org.uk<br>The Institute for Fiscal Studies<br>Tel: 020 7291 4800<br>7 Ridgmount Street<br>London<br>WC1E 7AE
Contact for Scientific Queries	Dr Sonya Krutikova (sonya_k@ifs.org.uk)<br>The Institute for Fiscal Studies<br>Tel: 020 7291 4800<br>7 Ridgmount Street<br>London<br>WC1E 7AE
Public Title	Evaluating an early childhood care and education program brought to scale in Ghana
Scientific Title	Lively Minds: Improving health and development through play - Evaluation of a comprehensive ECCE programme at scale in Ghana
Countries of Recruitment	Ghana
Health Condition(s) or Problem(s) Studied	Child development
Interventions	Play schemes for pre-school children.
Key Inclusion and Exclusion Criteria	1. Be aged between 3 and 5 years<br>2. Be enrolled or planning to enroll in the sample preschool in the coming term
Study Type	Interventional, open label cluster randomized control trial.

For peer review only - http://bmjopen.bmj.com/site/about/guidelines.xhtml

Date of First Enrolment	7/02/2022
Sample Size	3240 children
Recruitment Status	Incomplete
Primary Outcome	Measured 2 to 3 academic terms (6 - 9 months approximately) after treatment commences:<br>1. Child cognition as measured using the emergent numeracy, emergent literacy and executive functioning tasks in the IDELA as well as items relating to the same domains from the Harvard Laboratory for Development Studies<br>2. Child health, as measured using height/weight for age and height for weight Z score<br>3. Child socio-emotional development, as measured by the externalizing, internalizing and pro-social domains of the Strengths and Difficulty questionnaire and the socio-emotional items in the IDELA
Key Secondary Outcomes	Measured 2 to 3 academic terms (6 - 9 months approximately) after treatment commences:<br>1. Maternal mental health (Kessler-10 and WEMWBS) and knowledge of ECCE<br>2. Teacher knowledge, motivation and teaching quality (measured through classroom observations)<br>3. Parent-child investment, as measured by quality of the home environment (FCI and HOME) and the parent-child relationship (CPRS)<br>4. WASH practices in the home<br>5. Acute Malnutrition measured by MUAC
Ethics Review	Study protocols have been approved by ethics boards at University College London (IRB 21361/001), Yale University (IRB 2000031549) and Ghana Health Service Ethical Review Committee (IRB 028/09/21).
Completion date	September 2025
Summary Results	n/a as trial incomplete
IPD sharing statement	The data collected in the study will be publicly distributed along with critical documents (ie, protocols and questionnaires) following the publication of the primary results from the trials, which is expected to be within 24 months of the final data collection date.

For peer review only

For peer review only - http://bmjopen.bmj.com/site/about/guidelines.xhtml

Reporting checklist for protocol of a clinical trial.

Based on the SPIRIT guidelines.

Instructions to authors

Complete this checklist by entering the page numbers from your manuscript where readers will find each of the items listed below.

Your article may not currently address all the items on the checklist. Please modify your text to include the missing information. If you are certain that an item does not apply, please write "n/a" and provide a short explanation.

Upload your completed checklist as an extra file when you submit to a journal.

In your methods section, say that you used the SPIRITreporting guidelines, and cite them as:

Chan A-W, Tetzlaff JM, Gøtzsche PC, Altman DG, Mann H, Berlin J, Dickersin K, Hróbjartsson A, Schulz KF, Parulekar WR, Krleža-Jerić K, Laupacis A, Moher D. SPIRIT 2013 Explanation and Elaboration: Guidance for protocols of clinical trials. BMJ. 2013;346:e7586

For peer review only

		Reporting Item	Page Number
**Administrative information**			
Title	#1	Descriptive title identifying the study design, population, interventions, and, if applicable, trial acronym	1

Trial registration	#2a	Trial identifier and registry name. If not yet registered, name of intended registry	2
Trial registration: data set	#2b	All items from the World Health Organization Trial Registration Data Set	Supplementary Materials S5.
Protocol version	#3	Date and version identifier	12
Funding	#4	Sources and types of financial, material, and other support	11
Roles and responsibilities: contributorship	#5a	Names, affiliations, and roles of protocol contributors	1, 11
Roles and responsibilities: sponsor contact information	#5b	Name and contact information for the trial sponsor	11
Roles and responsibilities: sponsor and funder	#5c	Role of study sponsor and funders, if any, in study design; collection, management, analysis, and interpretation of data; writing of the report; and the decision to submit the report for publication, including whether they will have ultimate authority over any of these activities	11
Roles and responsibilities: committees	#5d	Composition, roles, and responsibilities of the coordinating centre, steering committee, endpoint adjudication committee, data management team,	n/a, no committees relevant

For peer review only

		and other individuals or groups overseeing the trial, if applicable (see Item 21a for data monitoring committee)	
**Introduction**			
Background and rationale	#6a	Description of research question and justification for undertaking the trial, including summary of relevant studies (published and unpublished) examining benefits and harms for each intervention	2-3
Background and rationale: choice of comparators	#6b	Explanation for choice of comparators	2-3
Objectives	#7	Specific objectives or hypotheses	3-4
Trial design	#8	Description of trial design including type of trial (eg, parallel group, crossover, factorial, single group), allocation ratio, and framework (eg, superiority, equivalence, non-inferiority, exploratory)	6
**Methods: Participants, interventions, and outcomes**			

For peer review only

Study setting	#9	Description of study settings (eg, community clinic, academic hospital) and list of countries where data will be collected. Reference to where list of study sites can be obtained	4
Eligibility criteria	#10	Inclusion and exclusion criteria for participants. If applicable, eligibility criteria for study centres and individuals who will perform the interventions (eg, surgeons, psychotherapists)	7
Interventions: description	#11a	Interventions for each group with sufficient detail to allow replication, including how and when they will be administered	4-6
Interventions: modifications	#11b	Criteria for discontinuing or modifying allocated interventions for a given trial participant (eg, drug dose change in response to harms, participant request, or improving / worsening disease)	n/a
Interventions: adherance	#11c	Strategies to improve adherence to intervention protocols, and any procedures for monitoring adherence (eg, drug tablet return; laboratory tests)	n/a, trial is evaluating effect of policy at scale
Interventions: concomitant care	#11d	Relevant concomitant care and interventions that are permitted or prohibited during the trial	n/a
Outcomes	#12	Primary, secondary, and other outcomes, including the specific measurement variable (eg,	7-8

For peer review only

		systolic blood pressure), analysis metric (eg, change from baseline, final value, time to event), method of aggregation (eg, median, proportion), and time point for each outcome. Explanation of the clinical relevance of chosen efficacy and harm outcomes is strongly recommended	
Participant timeline	#13	Time schedule of enrolment, interventions (including any run-ins and washouts), assessments, and visits for participants. A schematic diagram is highly recommended (see Figure)	6
Sample size	#14	Estimated number of participants needed to achieve study objectives and how it was determined, including clinical and statistical assumptions supporting any sample size calculations	6-7
Recruitment	#15	Strategies for achieving adequate participant enrolment to reach target sample size	6-7

Methods: Assignment of interventions (for controlled trials)

For peer review only

Allocation: sequence generation	#16a	Method of generating the allocation sequence (eg, computer-generated random numbers), and list of any factors for stratification. To reduce predictability of a random sequence, details of any planned restriction (eg, blocking) should be provided in a separate document that is unavailable to those who enrol participants or assign interventions	6
Allocation concealment mechanism	#16b	Mechanism of implementing the allocation sequence (eg, central telephone; sequentially numbered, opaque, sealed envelopes), describing any steps to conceal the sequence until interventions are assigned	n/a, open label trial
Allocation: implementation	#16c	Who will generate the allocation sequence, who will enrol participants, and who will assign participants to interventions	6
Blinding (masking)	#17a	Who will be blinded after assignment to interventions (eg, trial participants, care providers, outcome assessors, data analysts), and how	n/a
Blinding (masking): emergency unblinding	#17b	If blinded, circumstances under which unblinding is permissible, and procedure for revealing a participant's allocated intervention during the trial	n/a

For peer review only

Methods: Data collection, management, and analysis

Data collection plan	#18a	Plans for assessment and collection of outcome, baseline, and other trial data, including any related processes to promote data quality (eg, duplicate measurements, training of assessors) and a description of study instruments (eg, questionnaires, laboratory tests) along with their reliability and validity, if known. Reference to where data collection forms can be found, if not in the protocol	7-8 (& supplementary materials S4)
Data collection plan: retention	#18b	Plans to promote participant retention and complete follow-up, including list of any outcome data to be collected for participants who discontinue or deviate from intervention protocols	7-8
Data management	#19	Plans for data entry, coding, security, and storage, including any related processes to promote data quality (eg, double data entry; range checks for data values). Reference to where details of data management procedures can be found, if not in the protocol	7-8

For peer review only

Statistics: outcomes	#20a	Statistical methods for analysing primary and secondary outcomes. Reference to where other details of the statistical analysis plan can be found, if not in the protocol	9-10
Statistics: additional analyses	#20b	Methods for any additional analyses (eg, subgroup and adjusted analyses)	9-10
Statistics: analysis population and missing data	#20c	Definition of analysis population relating to protocol non-adherence (eg, as randomised analysis), and any statistical methods to handle missing data (eg, multiple imputation)	9-10
**Methods: Monitoring**			
Data monitoring: formal committee	#21a	Composition of data monitoring committee (DMC); summary of its role and reporting structure; statement of whether it is independent from the sponsor and competing interests; and reference to where further details about its charter can be found, if not in the protocol. Alternatively, an explanation of why a DMC is not needed	n/a
Data monitoring: interim analysis	#21b	Description of any interim analyses and stopping guidelines, including who will have access to these interim results and make the final decision to terminate the trial	n/a

For peer review only

Harms	#22	Plans for collecting, assessing, reporting, and managing solicited and spontaneously reported adverse events and other unintended effects of trial interventions or trial conduct	n/a
Auditing	#23	Frequency and procedures for auditing trial conduct, if any, and whether the process will be independent from investigators and the sponsor	n/a
**Ethics and dissemination**			
Research ethics approval	#24	Plans for seeking research ethics committee / institutional review board (REC / IRB) approval	2
Protocol amendments	#25	Plans for communicating important protocol modifications (eg, changes to eligibility criteria, outcomes, analyses) to relevant parties (eg, investigators, REC / IRBs, trial participants, trial registries, journals, regulators)	10-11
Consent or assent	#26a	Who will obtain informed consent or assent from potential trial participants or authorised surrogates, and how (see Item 32)	7, 10-11
Consent or assent: ancillary studies	#26b	Additional consent provisions for collection and use of participant data and biological specimens in ancillary studies, if applicable	n/a

For peer review only

Confidentiality	#27	How personal information about potential and enrolled participants will be collected, shared, and maintained in order to protect confidentiality before, during, and after the trial	8
Declaration of interests	#28	Financial and other competing interests for principal investigators for the overall trial and each study site	11
Data access	#29	Statement of who will have access to the final trial dataset, and disclosure of contractual agreements that limit such access for investigators	11
Ancillary and post trial care	#30	Provisions, if any, for ancillary and post-trial care, and for compensation to those who suffer harm from trial participation	n/a
Dissemination policy: trial results	#31a	Plans for investigators and sponsor to communicate trial results to participants, healthcare professionals, the public, and other relevant groups (eg, via publication, reporting in results databases, or other data sharing arrangements), including any publication restrictions	10-11
Dissemination policy: authorship	#31b	Authorship eligibility guidelines and any intended use of professional writers	11

For peer review only

Dissemination policy: reproducible research	#31c	Plans, if any, for granting public access to the full protocol, participant-level dataset, and statistical code	n/a

Appendices

Informed consent materials	#32	Model consent form and other related documentation given to participants and authorised surrogates	Supplementary Materials S2. and S3.
Biological specimens	#33	Plans for collection, laboratory evaluation, and storage of biological specimens for genetic or molecular analysis in the current trial and for future use in ancillary studies, if applicable	n/a

None The SPIRIT Explanation and Elaboration paper is distributed under the terms of the Creative Commons Attribution License CC-BY-NC. This checklist can be completed online using https://www.goodreports.org/, a tool made by the EQUATOR Network in collaboration with Penelope.ai

For peer review only

BMJ Open

Lively Minds: Improving health and development through play – a randomised controlled trial evaluation of a comprehensive ECCE programme at scale in Ghana

Journal:	*BMJ Open*
Manuscript ID	bmjopen-2022-061571.R2
Article Type:	Protocol
Date Submitted by the Author:	09-Sep-2022
Complete List of Authors:	Augsburg, Britta; Institute for Fiscal Studies,<br>Attanasio, Orazio; Yale University<br>Dreibelbis , Robert; London School of Hygiene and Tropical Medicine, Disease Control Department; London School of Hygiene and T<br>Nketiah-Amponsah, Edward; University of Ghana,<br>Phimister, Angus; Institute for Fiscal Studies,<br>Wolf, Sharon; University of Pennsylvania, Graduate School of Education<br>Krutikova, Sonya; Institute for Fiscal Studies
**Primary Subject Heading**:	Evidence based practice
Secondary Subject Heading:	Health services research, Public health, Health policy
Keywords:	PRIMARY CARE, PUBLIC HEALTH, EDUCATION & TRAINING (see Medical Education & Training), Health policy < HEALTH SERVICES ADMINISTRATION & MANAGEMENT

SCHOLARONE™
Manuscripts

BMJ Open Protocol

Lively Minds: Improving health and development through play – a randomised controlled trial evaluation of a comprehensive ECCE programme at scale in Ghana

Authors[a]

For peer review only

1- Institute for Fiscal Studies
2- Yale University
3- London School of Hygiene and Tropical Medicine
4- University of Ghana
5- University of Pennsylvania

Contact Details:
Britta Augsburg (corresponding author): britta_a@ifs.org.uk
Orazio Attanasio: orazio.attansio@yale.edu
Robert Dreibelbis: Robert.Dreibelbis@lshtm.ac.uk
Edward Nketiah-Amponsah: enamponsah@ug.edu.gh
Angus Phimister: angus_p@ifs.org.uk
Sharon Wolf: wolfs@upenn.edu
Sonya Krutikova: sonya_k@ifs.org.uk

For peer review only - http://bmjopen.bmj.com/site/about/guidelines.xhtml

Britta Augsburg[1], Orazio Attanasio[1,2], Robert Dreibelbis[3], Edward Nketiah-Amponsah[4], Angus Phimister[1], Sharon Wolf[5], Sonya Krutikova[1].

ABSTRACT

Introduction

Many children in developing countries grow up in environments that lack stimulation, leading to deficiencies in early years' development. Several efficacy trials of early childhood care and education programmes (ECCE) have demonstrated potential to improve child development; evidence on whether these effects can be sustained once programmes are scaled is much more mixed. This study evaluates whether an ECCE programme shown to be effective in an efficacy-trial maintains effectiveness when taken to scale by the Government of Ghana (GoG). The findings will provide critical evidence to the GoG on effectiveness of a programme it is investing in, as well as a blueprint for design and scale up of ECCE programmes in other developing countries, which are expanding their investment in ECCE programmes.

Methods and Analysis

This study is a cluster randomized control trial, in which the order that districts receive the programme is randomized. A minimum sample of 3240 children and 360 schools will be recruited across 72 district-school cohort pairs. The primary outcomes are (i) child cognitive and socio-emotional development measured using the International Development and Early Learning Assessment tool, the Strengths and Difficulties Questionnaire, and tasks from the Harvard Laboratory for Development Studies, (ii) child health (measured using height/weight for age, height-for-weight Z scores). Secondary outcomes include (i) maternal mental health, (using Kessler-10 and Warwick Edinburgh Mental Wellbeing Scale), knowledge of ECCE practices, (ii) teacher knowledge, motivation, teaching quality (measured with classroom observation), (iii) parental investment (using the Family Care Index and Home Observation Measurement of the Environment and the Child Parent Relationship Scale), (iv) WASH practices (v) acute malnutrition (using MUAC). We will estimate unadjusted and adjusted intent-to-treat effects.

Ethics and dissemination

Study protocols have been approved by ethics boards at University College London (21361/001), Yale University (2000031549) and Ghanaian Health Service Ethics Review Committee (028/09/21). Results will be made available to participating communities, funders, the wider public and other researchers through peer-reviewed journals, conference presentations, social and print media and various community/stakeholder engagement activities.

Trial registration number

AEARCTR-0008500, ISRCTN15360698

Keywords

Child health, child development

ARTICLE SUMMARY

For peer review only - http://bmjopen.bmj.com/site/about/guidelines.xhtml

Strengths and Limitations of this Study

- This trial assesses the effect of a programme previously demonstrated to be effective in a small efficacy trial now adopted by government and being brought to scale.
- Scaling up effective interventions has proven challenging in the past; this study will provide much needed evidence on whether an intervention designed to be scalable can be effective.
- A challenge in this study is measuring and adjusting for differences in programme delivery quality across different districts.

For peer review only

For peer review only - http://bmjopen.bmj.com/site/about/guidelines.xhtml

INTRODUCTION

Over 250 million children worldwide under the age of 5 years are at risk of not achieving their developmental potential due to poverty, poor health and nutrition, and deficient care and stimulation[1].The majority of these children live in low- and middle-income countries (LMICs), 1.6 million in Ghana[2]. In Northern Ghana, the setting of this study, and where most families live on less than US$2 per day, 20% of under 5s are stunted and 39% of 3-4 years olds are off-track cognitively[3].

School enrolment alone does not offset these disadvantages: in spite of great global progress in primary school enrolment, around 125 million children lack age-appropriate skills around the world[4]. Years of exposure to poor environments have adverse long-run effects on children's cognitive and socio-emotional development, health and psychological well-being, and earning potential, increasing the risk of poverty, early marriage and parenthood[5]. A recent Lancet review highlights that 'children at elevated risk for compromised development due to stunting and poverty are likely to forgo about a quarter of average adult income per year' [6].

As shown in a substantial body of evidence and increasingly recognized by policymakers, Early Childhood Care and Education (ECCE) interventions offer an opportunity to ameliorate these detrimental effects at a time of heightened developmental sensitivity and malleability[7]. The Government of Ghana (GoG) has implemented some of the most advanced ECCE policies in the Sub-Saharan Africa (National Early Childhood Care and Development Policy in 2004 and 2 years of public pre-primary education in 2007), resulting in universal net enrollment[8]. The GoG, however, recognizes that significant challenges remain: A third of Ghanaian pre-school (KG) children lack the necessary skills to thrive in school, including early academic and behavioural skills, social-emotional development, and aspects of physical health including motor development[9] and deficits persist and grow through primary school, where a fourth of pupils do not meet all proficiency cut-offs[10].

In 2017/18, OA and SK led an efficacy trial of *Lively Minds* - a holistic ECCE programme which engages parents and pre-schools to achieve healthy child development of 3-5 years olds in rural Ghana and promotes inclusion of children with disabilities. The trial revealed a positive impact on child health, as well as cognitive and socio-emotional development, partly mediated by improvements in parenting practices[11].

As a result of this evidence, the Ghana Education Service (GES) plans to integrate the *Lively Minds* programme with core instruction across all pre-schools in Northern Ghana over the next four years aiming to cover over 4,000 pre-schools, reaching 1.3 million children, reducing costs to $7 per child per year (less than a third of the original cost, and significantly below typical group parenting programme costs in LMICs, estimated to be US$30–35 per child[12]). Ownership of the programme has been transferred from the NGO that developed it to GES. We will therefore refer to the programme as the *GES Lively Minds* (GES-LM) programme.

This study is a cluster randomized control trial (CRCT) evaluating this scale-up, with randomization achieved through randomization of the order in which districts begin to receive the program. We will assess whether the impacts found at efficacy trial stage are sustained and investigate key mechanisms underlying these in order to determine how implementation and cost-effectiveness could be further improved.

Objectives

The project's key research questions are as follows:

1. What is the impact of the GES-LM at scale on child development?

For peer review only - http://bmjopen.bmj.com/site/about/guidelines.xhtml

2. How does the impact of the intervention vary by child, teacher, classroom/school and district characteristics?
3. What role do intervention fidelity and compliance play (intensity of training, dosage, hygiene promotion, quality of play sessions, supervision by teachers and district education offices) and how can the design be adapted to maximise cost-effectiveness?
4. Are impacts achieved through changes within the classroom/school and/or the children's home environments, including teacher and parent behaviour?

METHODS AND ANALYSIS

Study Setting

The programme will be rolled out at scale in Northern Ghana, where prevalence of chronic malnutrition is nearly twice as high as the national average (at 33% and 19% respectively) and 60-70% of the population were classified as poor in 2017, compared to 23% nationally[13,14]. Baseline conditions among the sample in the efficacy trial reveal that one third of children (3-5 years) had acute malnutrition and had experienced diarrhoea in the last 30 days; less than a third could identify a shape such as a triangle and even fewer were able to sort figures based on colour and shape. Around half of the mothers were at risk of depression and only 13% reported that *anyone* in the household had played with the target child in the last 3 days. The children live in households of on average ten members in which only 20% ever attended school and even fewer are literate (8%). Household sanitary infrastructure is largely lacking - nearly 80% report that they defecate openly. Access to public infrastructure is dismal: one in three communities do not have access to electricity and more than half cannot be accessed for 1-2 months of the year due to lack of paved roads[15].

Intervention Description

The program developed by the NGO Lively Minds aims to provide pre-school age children with the foundations they need to succeed in school. As young children transition to school, they draw on multiple skills to succeed in the classroom, including early academic and behavioural skills, social-emotional development, and aspects of physical health including motor development[16,17]. Exposure to quality ECCE has the potential to improve school readiness across these various domains. Current conceptualizations of ECCE quality rest however not only on children's experiences in their classroom, but also on their home environments and point to the critical role that warm, nurturing, responsive and stimulating relationships with caregivers both at home and school play. Attachment theory focuses on the importance of consistent and sensitive interactions with teachers and parents[18]; constructivist learning theories focus on the development of cognitive skills through engaging in age-appropriate activities[19]. Further, regular communication between parents and schools allows them to work together toward children's learning and development and has been shown to improve longer-term academic outcomes for preschool and kindergarten children[20,21].

Building on this theoretical framework, the Lively Minds intervention has four core aims:

- To increase children's opportunities for scaffolded, individualized or small group learning experiences at home and at pre-school.
- To reduce harsh and corporal punishment while improving positive behaviour management ensuring that children receive responsive, warm, nurturing caregiving from their caregivers at pre-school and at home.
- To improve hygiene practices and nutritional content in order to improve children's physical health, while considering constraints parents face.

For peer review only - http://bmjopen.bmj.com/site/about/guidelines.xhtml

- To strengthen engagement of mothers with children's schools in order to facilitate a more holistic approach to children's care and learning, as well as empower mothers to have a say in the quality of education their children receive at school.

To this end the intervention consists of three key components:

(1) Play Schemes (PS) for children attending KG (age 3-5): Children learn through play rotating between five play stations set up in KG classrooms, each of which is run by a trained woman from the community (often the mother of one of the children participating). At each play station mothers teach a small group of children (no more than 5) using discovery-based teaching methods through simple games and activities such as puzzles, sorting by shape and size, recognising letters and numbers, reading books and building with blocks. Children who are not in one of the five groups play outdoor games led by one of the trained women while they wait for their turn. Children have to wash their hands with soap before participating in the PS so that they become sensitized to the importance of this practice.

(2) Parenting courses for the women who run the PS: Monthly parenting workshops which aim to increase awareness and understanding of the sensitive caregiving and stimulation that children need to thrive, the adverse effects of harsh punishment and reinforce behaviours encouraged by the program including practices that stimulate children's development and health.

(3) Training and supervision of KG teachers: KG teachers are the *trainers* within this program. They enrol mothers in the community to volunteer to implement the PS, train the mothers on how to do this, oversee implementation of the PS in their classrooms and lead the parenting workshops. In order to do this, KG teachers receive ongoing training on all aspects of the program and supervision visits from the central program team, situated within the Ghana Ministry of Education.

Figure 1 summarises the conceptual framework underpinning this program, highlighting key channels through which the program is expected to impact children's developmental outcomes and school readiness. A critical and distinguishing feature of the program is that through engaging KG teachers and mothers it has the potential to *simultaneously* improve the home and pre-school environments; typically, ECCE programs are designed to target one or the other.

Figure 1. Conceptual framework underpinning the Lively Minds intervention

Figure 1 here

The program is owned and implemented by the Ghana Government Education Service (GES). This is a key difference between the version of the program evaluated in the efficacy trial and the current version. At that time the program was owned by the Lively Minds NGO who engaged the GES in implementation but remained in the driving seat throughout, retaining control over program content and implementation strategy and leading on all aspects of program implementation and monitoring on the ground. Control and responsibility for all of these elements has now been transferred to GES. Lively Minds provides technical assistance; for example, it helps districts set up the necessary monitoring and accounting data-bases, attends key training sessions of district teams and KG teachers, responds to queries from the GES Working Group in charge of the program and accompanied the District officials to some of the monitoring visits.

Implementation is conducted at the district level i.e. once a district is enrolled in the program all of the KGs in the district are invited to participate. Before enrolment of districts begins,

For peer review only - http://bmjopen.bmj.com/site/about/guidelines.xhtml

Lively Minds NGO familiarises the regional GES office[ii] with the Lively Minds Program and trains a team tasked with mobilisation of the districts. Following this there are five main implementation stages starting from enrolment of the district into the program. These are summarised in Figure 2. Supplementary Material S1 provides a more detailed explanation of what each step entails.

Figure 2. GES Lively Minds implementation model

Figure 2 here

Trial Design

This study is an open label cluster randomized control trial, accommodating the phased and complete roll-out of the GES-LM program. The program will be scaled to all of Northern Ghana's 60 districts over 10 phases; each phase (equivalent to one school term) covering a district group (DG) of 6 districts, from September 2021 to September 2024 (Panel A, Figure 3). Researchers at the IFS randomized districts to DGs using R version 4.1.1. DGs will be enrolled into the evaluation study from January 2022 to September 2024 (hence leaving out DG1) in 6 evaluation cohorts (EC) (Panel B, Figure 3). In each EC, one DG is allocated at random into the treatment group and one to the control DG. DG6 and DG7 are used first as control and later as treatment DGs, relying on different cohorts of children (those starting school in 2022 and those in 2023) (in different schools). DG10 will provide the control group for EC5 and EC6 using different schools. Control DGs will begin to receive the programme 3 terms after being enrolled into the study, after endline data collection has been completed (with the exception of EC6, where control will receive the programme after 2 terms). Baseline data collection will start at the end of January 2022 (after participant enrolment) (Panel C, Figure 3). Data collection will proceed continuously through to the end of the project in early 2025. Figure 4 shows this design in a flow diagram, Figure 5 shows the participant timeline.

Figure 3. Project design

Figure 3 here

Figure 4. Randomization flow Diagram

Figure 4 here

Figure 5. Participant Timeline

Figure 5 here

Sample size and Sampling

Within each district, we plan to randomly sample 5 programme-eligible preschools (excluding efficacy trial schools) and a minimum of 9 children who are enrolled or about to enrol in the sampled preschool. In DG6, DG7 and DG10 we will sample children and schools twice (once for each cohort of children). When we sample for a second time, we will exclude schools

[ii] There are currently 16 regions in Ghana made up of 261 districts.

For peer review only - http://bmjopen.bmj.com/site/about/guidelines.xhtml

previously enrolled into the study. This gives us a minimum total sample of 360 preschools and 3460 children and households.

Schools will be selected at random from a complete school list in each district provided by the GES. We will exclude focus on government schools, and exclude atypical schools, specifically only boys' and only girls' schools (more than 99% of government schools are mixed gender), as well as schools with less than 20 enrolled students. Doing so we exclude 20 schools from the sampling frame (20 small ones, one of which has no boys enrolled in KG).

Our study participants will be children, and their households, that attend, or are due to attend KG. These will be identified from school enrolment lists, except for EC1 where due to the COVID-19 pandemic, school openings were different and lists were not readily available. We therefore sampled in this EC only from a census surrounding the school. To be eligible for the study children must fulfil both of the following criteria (the same as in the efficacy trial):

1. Be aged between 3 and 5 years
2. Be enrolled or planning to enrol in the sample preschool in the coming term

Where more than one child is eligible within a single household we will randomly select one child to be a part of the study. Primary care givers will be asked for their consent (and to give consent on behalf of their child) before being enrolled in the study. Model consent form and information sheet for primary caregivers are provided in Supplementary Materials S2. And S3.

Outcomes

In each sampled unit (school) we intend to conduct 4 separate surveys/assessments types, each to be implemented both at baseline and at endline, 2-3 school terms later. Surveys/assessments include: (1) child assessment, (2) Primary caregiver (PCG) Interview, (3) Kindergarten teacher interview and classroom observation, (4) community survey. These surveys will be collected by local well-trained and experienced interviewers, who will not be informed about treatment allocation.

In order to gather evidence on district-level preparedness for the programme (for later heterogeneity analyses), we additionally plan to conduct a survey of the district official in charge of preschool programmes in each district once. We will also collect secondary data available on districts, such as languages spoken, or voter turnout.

We have three sets of primary outcomes:

1. Child cognitive development measured using the International Development and Early Learning Assessment (IDELA) tool[22] and tasks from the Harvard Laboratory for Development Studies, all previously used in Ghana.
2. Child socio-emotional development measured using Strengths and Difficulties Questionnaire (SDQ)[23,24] administered to the primary care giver.
3. Child health measured using height/weight for age and height for weight Z scores, following WHO guidelines.

Our secondary outcomes are as follows:

1. Maternal mental health, measured used the Kessler-10 and WEMWBS and knowledge of ECCE practices;
2. Teacher knowledge, motivation and teaching quality, measured using classroom observation;
3. Parental investment measured through quality in the home environment (Family Care Index and Home Observation Measurement of the Environment) and the quality of the parent-child relationship (CPRS);

For peer review only - http://bmjopen.bmj.com/site/about/guidelines.xhtml

4. WASH practices in the home (e.g. hand washing practices, toilet facilities, open defecation practices);
5. Acute malnutrition measured using middle upper arm circumference. Beyond being of interest in its own right, this is the health outcome used in the efficacy trial[25], which will allow us to compare outcomes across both trials.

In selecting measures for our key outcomes, we prioritised those which have already been validated and used in the study context (for example IDELA[22,26]) or in a context that is similar. Since very little related work has been conducted in our study context, this was not possible for all outcomes that we want to capture. For these outcomes we next identified measures which have been widely used and validated across multiple contexts, for example the Kessler-10 and FCI. We will adapt these measures to the study context following best practice[27]: tools will be reviewed for socio-cultural relevance and linguistic accuracy through consultation with local professionals and modified accordingly; tools will be translated and back-translated into the all of the main languages spoken in the study communities. Full validation of the tools before administration of the baseline is beyond the scope of this study. However, all outcome measurement tools will be piloted on small samples in the study area, feedback on performance will be collected from the surveyors and further adaptations will be made. As part of the training the survey team will be required to achieve inter-rater reliability of at least 0.8 (kappa statistic) on the child assessment and classroom observation tools. Once data are collected we will assess reliability and validity of the outcome measures by examining whether any items have a lot of missing responses, items show sufficient variability, correlations with other variables go in the expected direction, measures exhibit sufficient degree of internal consistency (Cronbach's alpha of at least 0.7). We will also assess dimensionality of the measures using exploratory and confirmatory factor analysis and functioning of individual items using Item Response Theory to generate item characteristics curves. More detailed information on these outcomes is provided in Supplementary Material S4.

Data management protocols and Data Statement

For details on data management please refer to the project's management registration: https://ifs.org.uk/uploads/Research%20registraion%20form.pdf. Data availability is outlined in Supplementary Material S5.

Sample Size and Power

As our design does not deviate in a substantive way from a conventional two-arm cluster randomized control trial design (CRCT), we use standard CRCT methods to assess statistical power. For the power analysis, we consider the district as a cluster, providing us with 54 clusters, of size 45 each – 5 schools per cluster, 9 children per school. We leverage data available from the efficacy trial to better inform how covariates increase our power to detect effects, including expected loss to follow-up, which we set at 6%.

We calculate minimum detectable effects (MDEs) using the following formula[28]:

$$MDE = \left(\frac{\left(t_{\frac{\alpha}{2}} + t_{\beta}\right)^2 \sigma^2 \left(m\rho\left(1 - R_c^2\right) + (1-\rho)\left(1 - R_p^2\right)\right)}{n} \right)^{\frac{1}{2}} \quad (1)$$

For peer review only - http://bmjopen.bmj.com/site/about/guidelines.xhtml

α denotes the significance level, β power, ρ unconditional ICC, t is are the critical values of the t-distribution, σ is the standard deviation of the outcome, n is children per arm, m is children per cluster, R_c^2 is proportion of cluster level variance component explained by covariates and R_p^2 is the individual equivalent. One can interpret the effect of R_c^2 and R_p^2 as altering the "variance inflation factor" of the power calculation – when they equal zero the formula reduces to the standard CRCT one. We set α to 0.05, β to 0.8.

We estimate the intra-cluster correlation (ICC) for each outcome data using data from the efficacy trial (ranging from 0.011 to 0.110). Note we cannot conduct the same exercise for all of our primary outcomes, as some were not included in the original trial. The minimum detectable effects for some of our primary and secondary outcomes measured at the child/household level are shown in Table 1. The power to detect effects at the school level is significantly more limited.

Table 1. Minimum Detectable Effects

Outcome	MDE
Cognition factor score	0.118
Middle Upper Arm Circumference	0.132
Knowledge Infant Development Index (KIDI)	0.125
Family Care Index (FCI)	0.227
Parent-child interaction (PRC)	0.132

Note: Each outcome controls for the its baseline value only.

The MDE for the cognition score will allow us to determine whether the scaled up, government owned and run version of the Lively Minds program is at least as effective as the NGO-run smaller scale version evaluated in the efficacy trial in its key aim of improving cognitive development of pre-school children. However, if the scale up results in a significant reduction in effectiveness, we will not be able to determine whether the program continues to have an impact. It should be noted that efficacy trial impacts were largest for the poorer children in the sample. On average the areas where the scale-up is happening are poorer than those where the efficacy trial took palace so even if there is some loss of effectiveness as the result of the scale-up we should still be able to detect impacts given our power.

Analysis Plan

Intention to Treat

For our primary and secondary outcomes, we will estimate an intent-to-treat effect of the program. Our design is the same as a cluster randomized control trial stratified by evaluation cohort. As such, we follow the standard estimation technique for two-way stratified cluster-randomised control trials, For the outcome variable Y of individual i in district j at date t, we estimate the following:

$$Y_{ijt} = \mu + \gamma_t + \theta\, Treatment_{ijt} + \boldsymbol{\beta}\, \boldsymbol{X_{ijt}} + \varepsilon_{ij}$$

Where γ_t is an evaluation cohort fixed effect, $Treatment_{ijt}$ is binary indicator variable equal to 1 if individual i is in a treatment district at date t, $\boldsymbol{X_{ijt}}$ is a set of controls variables. The treatment effect we identify is θ, and standard errors will be clustered at the school level.

For peer review only - http://bmjopen.bmj.com/site/about/guidelines.xhtml

We will compute the Romano-Wolf stepdown p-values to adjust for multiple hypothesis testing. Hypotheses will be arranged within families of outcomes - i.e. each set of research questions separately.

The set of controls $\boldsymbol{X_{ijt}}$ will be identified using a double-lasso procedure of the covariates we collect as part of our data collection. We will present estimates of the treatment effect both with and without these control variables. In addition to those controls that the double-lasso procedure generates, we will use (at a minimum) the following controls:

- Mother age (quadratically)
- Mother Education attainment
- Tester fixed effects (where relevant)
- Time between baseline and endline measurements
- Child Age
- Child Sex at birth
- Baseline value of the outcome variable of interest

Heterogeneity

We will explore heterogeneity in treatment effect in two distinct ways.

We are first interested in considering treatment heterogeneity by baseline characteristics of the child, their home environment, and features of the environment at the preschool and district-level. Given the large number of relevant dimensions, we will use recent methodological advances[29] to assess heterogeneity across all of these dimensions in a rigorous and data-driven way.

Second, we will assess how study impacts vary by implementation fidelity and compliance in different dimensions. Given intervention fidelity measures are only observed in treatment districts, we will also examine heterogeneity from the data collected as part of the district official survey, focusing on those covariates that are most predictive of high fidelity in treatment districts.

Stopping Rules

Whilst there is always a risk of unintended consequences in all types of trials, in this sort of intervention such a risk is minimal. However, if there is any clear evidence of harm then the study will halt under international ethical guidelines for medical research.

Additional Analysis

This is a large study with many collaborators, and the data gathered will be able to answer more scientific questions than those outlined in this protocol. The study team expects to conduct and publish such additional analyses.

Patient and Public Involvement

Patients or the public were not involved in the design of our research.

ETHICS AND DISSEMINATION

The trial is overseen by the University College London Research Ethics Committee, the Human Research Protection Program Institutional Review Boards at Yale University as well as by the Ghanaian Health Service Ethics Review Committee, which have reviewed the study protocols.

For peer review only - http://bmjopen.bmj.com/site/about/guidelines.xhtml

Particular consideration will be given to potentially vulnerable people or groups, especially children, and informed consent will be acquired by IPA Staff from all parents of participating children before the commencement of any data collection, and that they can stop participating at any time without providing a reason. Model consent forms are available upon request. Any reports of abuse or dangers to children will be reported to relevant local authorities.

Important protocol modifications will be communicated to all relevant parties (e.g. investigators, REC/IRBs, trial participants, trial registries, journals).

Data will be collected, handled, transferred and secured in a manner which aligns with international best practice. The IFS information security management system is ISO27001 compliant.

We will publish findings through (a) top academic journals in economics and developmental science; (b) presentations at conferences in Ghana, US, and Europe; (c) publication of high impact policy briefs for the wider public; (d) leaflets to communicate findings to participating communities; and (e) partner's websites and social media.

Whilst there is always a risk of unintended consequences in all types of trials, in this sort of intervention such a risk is minimal. However, if there is any clear evidence of harm then the study will halt under international ethical guidelines for medical research.

Contributorship Statement

SK, BA, SW, ENA, RD and OA conceptualized the design and methods of the study and revised the manuscript and approved the final manuscript as submitted. AP drafted the initial manuscript, BA revised the manuscript and approved the final manuscript as submitted. All authors provided input and guidance on study design. All authors approved the final manuscript as submitted and agree to be accountable for all aspects of this systematic review

Competing interests

All authors have no competing interests to declare.

Funding

This study is funded by USAID (grant 7200AA21FA00016) and the Medical Research Council (grant MR/V035312/1). This funding source had no role in the design of this study and will not have any role during its execution, analyses, interpretation of the data, or decision to submit results.

Data sharing

No data are available. The data collected in the study will be made publicly available following the publication of the primary results from the trials.

Word count: 4,293

For peer review only - http://bmjopen.bmj.com/site/about/guidelines.xhtml

REFERENCES

1 Grantham-McGregor, S, YB Cheung, S Cueto, P Glewwe, L Richter, B Strupp, *Lancet*, 2007;369:60–70.

2 Lu, C, M Black, L Richter, *The Lancet Global Health*, 2016 Dec; 4(12).

3 MICS snapshot report Ghana 2017-18.

4 UNESCO, EFA Global Monitoring Report, 2013/14.

5 Gertler, P, J Heckman, R Pinto, A Zanolini, C Vermeerch, S Walker, SM Chang, S Grantham-McGregor, *Science*. 2014 May 30; 344(6187): 998–1001.

6 Richter, LM, B Daelmans, J Lombardi, J Heymann, FL Boo, JR Behrman, C Lu, JE Lucas, R Perez-Escamilla, T Dua, ZA Bhutta, *The Lancet*. 2017;389(10064):103-18.

7 Shonkoff, JP, DA Phillips, National Academy Press, 2016.

8 Ministry of Education Ghana, 'Education Strategic Plan 2018-2030'.

9 McCoy,DC , ED Peet, M Ezzati, G Danaei, M Black, CR Sudfeld, W Fawzi, G Fink, *PLoS Medicine*, 2016, 13(6).

10 Ministry of Education, Ghana Education Service, 'National Education Assessment' Technical Report 2016.

11 Attanasio, O, B Caeyers, L Cardona-Sosa, S Cattan, S Krutikova, P Leighton (2020). Link.

12 Horton, S, MM Black. (2017), Chapter 24 in: Bundy DAP, ND Silva, S Horton, et al., editors. Child and Adolescent Health and Development. 3rd edition. Washington (DC).

13 USAID, 2018 "Ghana: Nutrition Profile". Link.

14 Ghana Living Standard Survey, 2016/17.

15 Pisani, L, I Borisova, AJ Dowd (2018), *International Journal of Educational Research*, 91: 1–15. https://doi.org/10.1016/j.ijer.2018.06.007

16 *Snow*, C.E., *Van Hemel*, S.B. The National Academies Press, *2008.*

17 UNESCO (2013). UNESCO-UIS/Brookings Institution. Link.

18 Ainsworth, MDS (1989). *American Psychologist*, 44, 709-716. http://dx.doi.org/10.1037/0003-066X.44.4.709.

19 Gopnik, A, AN Meltzoff, and PK Kuhl. (1999). *The Scientist in the Crib: Minds, Brains, and How Children Learn.* New York, NY: William Morrow & Co.

20 Miedel, WT and AJ Reynolds. (2000). *Journal of School Psychology, 37,* 379–402.

21 Connor, CM, SH Son, AH Hindman, FJ Morrison. (2005). *Journal of School Psychology, 43*(4), 343–375.

22 Pisani, L, I Borisova, AJ Dowd. (2015). Washington, DC. Link.

23 Hoosen, N, EL Davids, PJ De Vries, M Shung-King. (2018). *Child and Adolescent Psychiatry and Mental Health* 12: 6.

24 Goodman, R., *Journal of Child Psychology and Psychiatry*, Jul 1997;38(5):581-6.

25 Amadu, S, O Attanasio, B Caeyers, S Cattan, L Cardona Sosa, S Krutikova, P Leighton, 2018, IFS Report 12996. Link.

26 Wolf, S, M Peele, *Journal of the British Academy*, 8(s2), 71–102

27 Fernald, LCH, E Prado, P Kariger, A Raikes (2017). Washington DC. The World Bank. Link.

28 McConnell, B, M Vera-Hernandez, 2015, IFS Working Paper W15/17, Link.

29 Wager, S, S Athey, *J of the American Statistical Association*, 2018: 113:523, 1228-1242.

Date and Version Number

02/09/2022, version 3

For peer review only - http://bmjopen.bmj.com/site/about/guidelines.xhtml

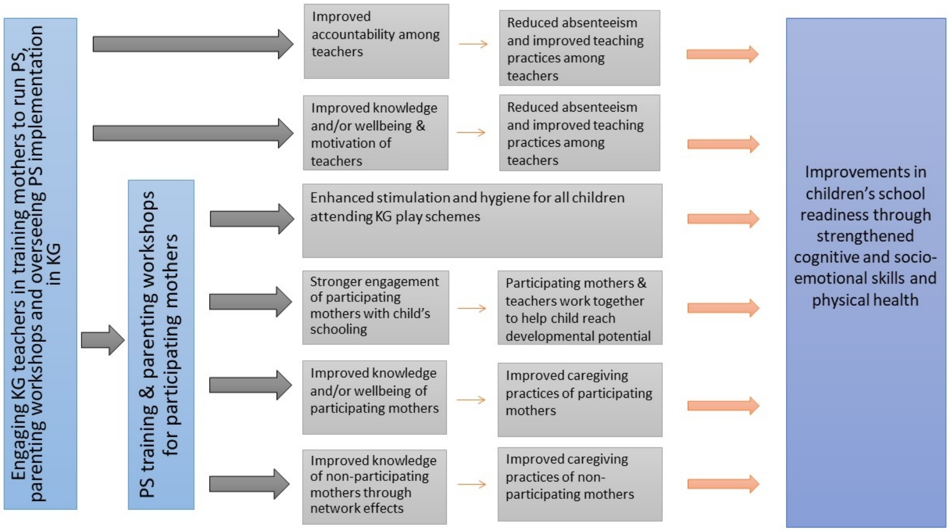

Figure 1. Conceptual framework underpinning the Lively Minds intervention

338x190mm (96 x 96 DPI)

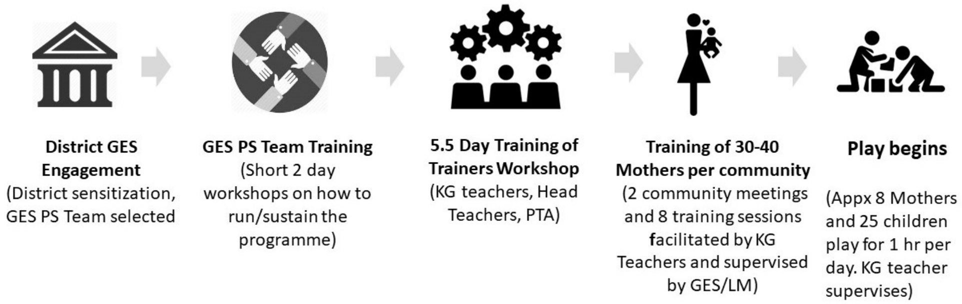

Figure 2. GES Lively Minds implementation model

329x109mm (96 x 96 DPI)

				School Term:	Sep-21	Jan-22	May-22	Sep-22	Jan-23	May-23	Sep-23	Jan-24	May-24	Sep-24	Jan-25
**Panel A**: Intervention roll-out<br>1=intervention implemented<br>0=no intervention															
6 districts	DG1				1	1	1	1	1	1	1	1	1	1	1
6 districts	DG2				0	1	1	1	1	1	1	1	1	1	1
6 districts	DG3				0	0	1	1	1	1	1	1	1	1	1
6 districts	DG4				0	0	0	1	1	1	1	1	1	1	1
6 districts	DG5				0	0	0	0	1	1	1	1	1	1	1
6 districts	DG6				0	0	0	0	0	1	1	1	1	1	1
6 districts	DG7				0	0	0	0	0	0	1	1	1	1	1
6 districts	DG8				0	0	0	0	0	0	0	1	1	1	1
6 districts	DG9				0	0	0	0	0	0	0	0	1	1	1
6 districts	DG10				0	0	0	0	0	0	0	0	0	1	1
**Panel B**: Treatment and control allocation & cohort selection															
6 districts	DG1				1	1	1	1	1	1	1	1	1	1	1
6 districts	DG2	EC1	Treatment	Cohort 2022	0	1	1	1	1	1	1	1	1	1	1
6 districts	DG6		Control	Cohort 2022	0	0	0	0	0	1	1	1	1	1	1
6 districts	DG3	EC2	Treatment	Cohort 2022	0	0	1	1	1	1	1	1	1	1	1
6 districts	DG7		Control	Cohort 2022	0	0	0	0	0	0	1	1	1	1	1
6 districts	DG4	EC3	Treatment	Cohort 2022	0	0	0	1	1	1	1	1	1	1	1
6 districts	DG8		Control	Cohort 2022	0	0	0	0	0	0	0	1	1	1	1
6 districts	DG5	EC4	Treatment	Cohort 2023	0	0	0	0	1	1	1	1	1	1	1
6 districts	DG9		Control	Cohort 2023	0	0	0	0	0	0	0	0	1	1	1
6 districts	DG6	EC5	Treatment	Cohort 2023	0	0	0	0	0	1	1	1	1	1	1
6 districts	DG10		Control	Cohort 2023	0	0	0	0	0	0	0	0	0	1	1
6 districts	DG7	EC6	Treatment	Cohort 2023	0	0	0	0	0	0	1	1	1	1	1
6 districts	DG10		Control	Cohort 2023	0	0	0	0	0	0	0	0	0	1	1
**Panel C**: Baseline and endline data collection timing															
6 districts	DG1				1	1	1	1	1	1	1	1	1	1	1
6 districts	DG2	EC1	Treatment	Cohort 2022	0	1	1	1	1	1	1	1	1	1	1
6 districts	DG6		Control	Cohort 2022	0	0	0	0	0	1	1	1	1	1	1
6 districts	DG3	EC2	Treatment	Cohort 2022	0	0	1	1	1	1	1	1	1	1	1
6 districts	DG7		Control	Cohort 2022	0	0	0	0	0	0	1	1	1	1	1
6 districts	DG4	EC3	Treatment	Cohort 2022	0	0	0	1	1	1	1	1	1	1	1
6 districts	DG8		Control	Cohort 2022	0	0	0	0	0	0	0	1	1	1	1
6 districts	DG5	EC4	Treatment	Cohort 2023	0	0	0	0	1	1	1	1	1	1	1
6 districts	DG9		Control	Cohort 2023	0	0	0	0	0	0	0	0	1	1	1
6 districts	DG6	EC5	Treatment	Cohort 2023	0	0	0	0	0	1	1	1	1	1	1
6 districts	DG10		Control	Cohort 2023	0	0	0	0	0	0	0	0	0	1	1
6 districts	DG7	EC6	Treatment	Cohort 2023	0	0	0	0	0	0	1	1	1	1	1
6 districts	DG10		Control	Cohort 2023	0	0	0	0	0	0	0	0	0	1	1

Figure 3. Project design

165x126mm (150 x 150 DPI)

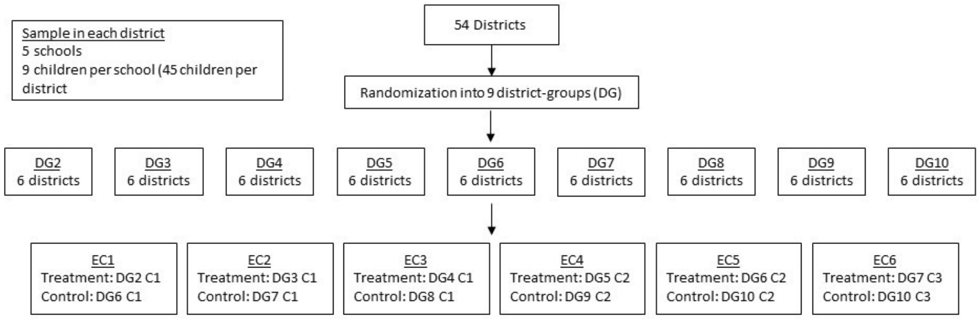

Figure 4. Randomization flow Diagram

252x85mm (96 x 96 DPI)

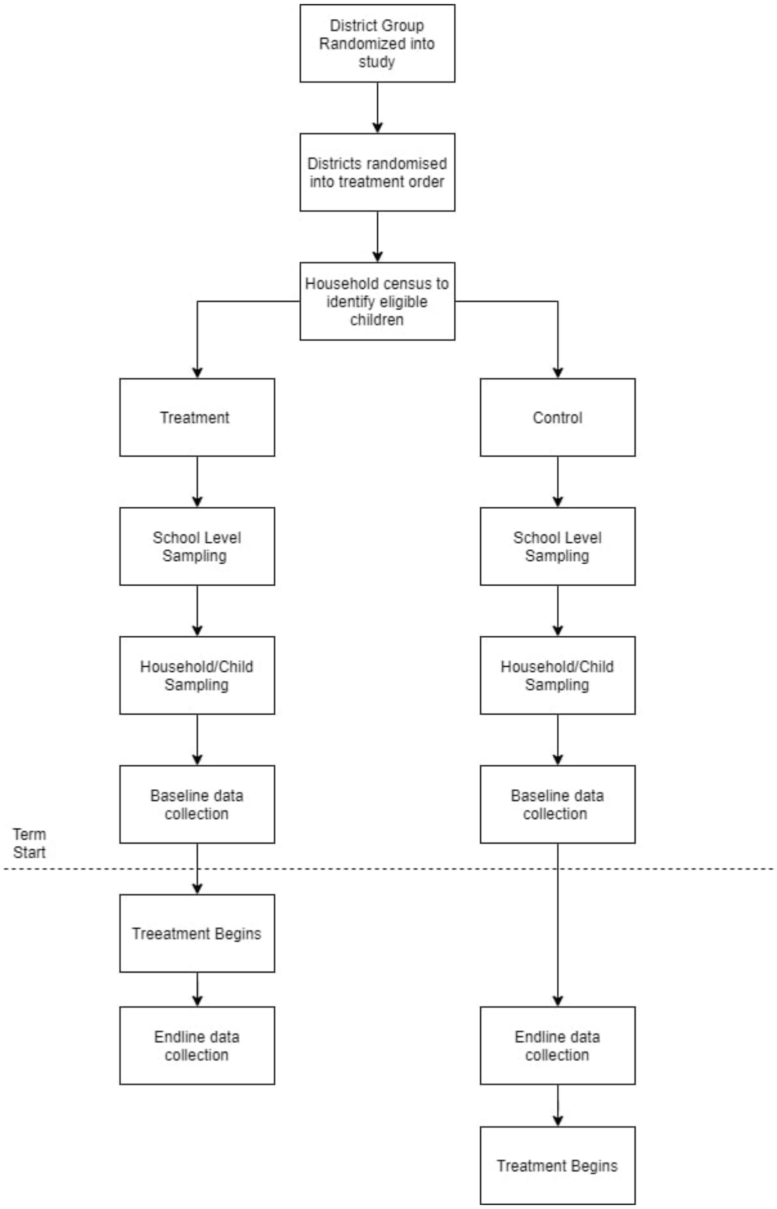

Figure 5. Participant Timeline

212x328mm (72 x 72 DPI)

Supplementary materials

This appendix has been provided by the authors to give readers additional information about the study.

Supplement to: "*Lively Minds: Improving health and development through play – a randomised controlled trial evaluation of a comprehensive ECCE programme at scale in Ghana*"

For peer review only

For peer review only - http://bmjopen.bmj.com/site/about/guidelines.xhtml

S1. GES Lively Minds implementation model:

This supplementary material provides additional information to the intervention description.

1. Regional GES team, with support from Lively Minds NGO, conducts sensitisation of District level GES officials, introducing the program to them, explaining its importance and securing their commitment to implementing it. The District GES appoints a "Play Scheme" (PS) team consisting of District GES staff tasked with leading implementation of the program in all public KGs in the district.
2. Regional GES team conduct 2-day workshop with the District level GES PS team on how to run and sustain the programme.
3. The District GES PS team conducts sensitisation with schools in the district, which identify KG teachers that meet participation criteria to receive training of trainers[i]. These KG teachers along with the Head Teachers from the schools in which they teach attend a 5.5-day Training of Trainers workshop in which they are introduced to the GES Lively Minds programme, how to mobilise mothers to participate in the program, how to train the mothers and how to run the program in their KGs.
4. KG teachers conduct 2 community meetings to introduce the programme and ask for women in the community to volunteer to implement the PS at the KG during school time[ii]. The women who express interest are trained by the KG teachers over the course of 8 sessions under the supervision of the District PS team and Lively Minds NGO representative.
5. Play Schemes begin in KGs – the trained mothers run the Play Schemes in the KGs at least 4 times per week in 2-hour sessions, supervised by the KG teacher. These mothers also attend a monthly parenting workshop led by the KG teacher.

There is ongoing supervision of the KG teachers and Play Scheme implementation within KGs as well as of the parenting courses by the District PS team. This is done through regular unannounced visits by District GES PS team members who complete monitoring forms to record their findings during the visit. District GES PS Team delivers top-up training for the KG teachers and Head Teachers of participating schools twice per term.

---

[i] The school must have 2 KG teachers in place who are on permanent contracts with GES and speak the local language fluently.
[ii] There is no requirement that the participants are mothers, though most of those who sign up have young children.

For peer review only - http://bmjopen.bmj.com/site/about/guidelines.xhtml

S2. Model consent form – Primary Caregiver:

Consent Form for Primary Caregivers (Primary Caregiver Survey)
INNOVATIONS FOR POVERTY ACTION
Lively Minds Evaluation Extension

Consent Form for Primary Caregivers (Primary Caregiver Survey)

Study Title: Evaluation of a Pre-school Program Implemented at Scale in Ghana (Protocol ID NO: GHC-ERC Number: 028/09/21)

Good day, my name is ___________________________I work for Innovations for Poverty Action (IPA), a research and policy non-profit organization that discovers and promotes effective solutions to global poverty problems.

Purpose of the study

IPA is working with researchers at the Institute for Fiscal Studies (UK), Yale University (USA), University of Pennsylvania (USA) and the University of Ghana to learn more about young children and about how we can improve children's learning outcomes. This study is paid for the by United States Agency for International Development, and the Medical Research Council of the United Kingdom.

We have received official permission from the Ghana Education Service to conduct this survey. We are inviting you and your children to participate in this study.

Procedures

If you choose to participate, you will be asked to complete a survey about your background and household information, caregiver's school engagement activities, household assets, household economic activities, agricultural activities and food insecurity, household savings and credit, household economic shocks and risks, caregiver and children time use, caregiver social capital, school choice and beliefs about quality education, water, sanitation, and hygiene practices, parent-child relationship, caregiver cognitive skills, mental health and wellbeing, caregiver empowerment, children behaviours, and caregiver volunteering activities. This survey will take about 1 hour and 30 minutes of your time.

If you agree that your children participate in this research, they will be asked to participate in an assessment on numeracy, literacy, and social-emotional skills at their school. If you agree that your children participate in this research, they will be asked to participate in an assessment on numeracy, literacy, and social-emotional skills at their school through play-based tasks and take basic anthropometric measurements (i.e. height, weight, arm circumference) - always in the presence of the child's teacher or adult in a similar role. Enumerators will also observe teaching and take inventory of classroom practices. As part of this, we will record a short video of a class while it is in session.

You and your child may be contacted to participate in a follow-up survey or another study at a future date, but you are free to decline participation in the follow-up survey or another study if you wish.

We hope to record a portion of this interview for quality control purposes. This is voluntary, and you are free to decline if you do not wish to be recorded. All video and audio recordings will be destroyed after five years.

We wish to record the GPS coordinates of this interview for tracking purposes. This does not pose any additional risk to you and is voluntary, so, you are free to decline if you feel uncomfortable.

Confidentiality

For peer review only

The responses you and your children provide will be kept confidential to the extent possible and will only be accessible to the research team and individuals from IPA who oversees the research. Only data or information that does not identify your teachers and pupils in any way may be (a) used for research and academic publications or (b) shared with other people or organizations. It will not be possible to know that a given answer has been provided by your or your children.

While we will take all precautions to prevent this, in the event that there is an outside breach of confidentiality of the data, you will be contacted and informed of the event and the steps being taken to recover your information.

The legal basis for collecting and processing this information in the United Kingdom is "legitimate interests" (Article 6(1)(f)) of the General Data Protection Regulation 2018 and in Ghana is Data Protection Act, 2012 (ACT 843)

Voluntary Participation

Participation is completely voluntary. You and your children are free to (a) decline to participate; (b) withdraw from the study at any time, and (c) skip any question they feel uncomfortable answering without penalty or loss of any existing benefits for doing so.

Risks and Benefits

There are no anticipated risks from taking part in this survey. However, there may be inconvenience and loss of time in participating in this study. All data collection activities will be conducted in strict adherence to the covid-19 protocols including wearing nose masks to reduce the likelihood of either party contracting the virus. There are no direct benefits to you and your children from participating in the survey. However, the information you would provide will help us design an effective education policy in Ghana.

There are no direct benefits to you from participating in the survey. However, the information you would provide will help us design an effective education policy in Ghana.

Contact Information

If you have questions about the research study, you can contact IPA at +233 050 251 4712 and ask for Murphy Edro, the Research Associate. You can also contact the lead researchers directly by emailing them at ghanasurvey@ifs.org.uk.If you have any questions about your rights as a research participant, or complaints, contact Ghana Health Service Ethics Committee at 0503539896 (ask for Nana Abena Apatu) or by emailing them at ethics.research@ghsmail.org. You can also contact UCL Ethics by emailing ethics@ucl.ac.uk.

Do you have any questions that I can answer?

PARTICIPANTS' STATEMENT

I acknowledge that I have read or have had the purpose and contents of the Participant's Consent and Information Sheet read and all questions satisfactorily explained to me in a language I understand (____). I fully understand the contents and any potential implications as well as my right to change my mind (i.e., withdraw from the research) at any point. I understand that if I withdraw before January 2025, I can ask for my data not to be used.

For participation in this research:

1. |___| I voluntarily agree to be part of this research.
2. |___| I voluntarily agree for my children only to be part of this research
3. |___| I voluntarily agree for me and my children to be part of this research
4. |___| I don't agree with any of us being part of this research.

For peer review only - http://bmjopen.bmj.com/site/about/guidelines.xhtml

For audio recording:

1. |__| I voluntarily agree to the audio recording for me only
2. |___| I voluntarily agree to the audio recording for my children only
3. |___| I voluntarily agree to the audio recording for me and my children
4. |___| I don't agree with the audio recording for any of us

For GPS records, I voluntarily agree to the taking of GPS records for my dwelling. |__| Yes |__|No

Name of Participant____

Participant's Signature_____________________________________ OR Thumb Print_________________________________

Date___________________________________

INTERPRETERS' STATEMENT

I interpreted the purpose and contents of the Participant's Consent and Information Sheet to the afore named participant to the best of my ability in the (___________________________________) language to his proper understanding. All questions, appropriate clarifications sort by the participant and answers were also duly interpreted to his/her satisfaction.

Name of Interpreter____

Interpreter's Signature_____________________________________ OR Thumb Print__________________

Date___________________________________

STATEMENT OF WITNESS

I was present when the purpose and contents of the Participant Consent and Information Sheet was read and explained satisfactorily to the participant in the (___________________________________) language, which s/he understood. I confirm that s/he was given the opportunity to ask questions/seek clarifications and same were duly answered to his/her satisfaction before voluntarily agreeing to be part of the research.

Name of Witness____

Witness' Signature_____________________________________ OR Thumb

Print__________________________

Date___________________________________

INTERVIEWER STATEMENT AND SIGNATURE

I certify that the participant has been given ample time to read and learn about the study. All questions and clarifications raised by the participant have been addressed.

Interviewer's name____

Signature____

Date___________________________________

For peer review only - http://bmjopen.bmj.com/site/about/guidelines.xhtml

S3. Model information sheet – Primary Caregiver:

Evaluation of a pre-school program implemented at scale in Ghana

This study has been approved by the UCL Research Ethics Committee (Project ID Number): 21361/001

PARTICIPANT INFORMATION SHEET

Introduction

This document contains information to help you decide whether you would like to complete the survey. Please take your time to think about the points raised before making your decision about whether or not to participate. If there is anything that is not clear, you can ask me (the surveyor) or my supervisor. Or you can contact the researchers directly by emailing at ghanasurvey@ifs.org.uk. If you wish to raise a complaint, please see contact details below.

What is this research project for?

We are conducting this research to better understand the effectiveness of a preschool program in aiding child development, with researchers at the Institute for Fiscal Studies (UK), Yale (USA), University of Pennylvania (USA) and University of Ghana. Your participation is important to us! By participating in this study, you can help us learn more about how pre-school programs can improve child development, and make children readier to start school.

Why have I been chosen?

Your household has been chosen for this interview as you have a child attending the nearby kindergarten.

What will I be asked to do and what information will we collect?

In this survey we are collecting information on your household, as well as information on your child who has recently been enrolled in pre-school and you as the primary care giver.

The household component of the survey gathers information on demographics, your educational history as well as that of other household members, dwelling, land, livestock, assets, engagement in economic activities.

Afterward a caregiver of a selected child in this household will be interviewed separately for some information about the caregivers' background, child care practice and beliefs, social networks and physical and mental well-being, as well as some information about the development of the children in this community. I will also spend some time with one child from this household who is currently enrolled in kindergarten, to take him/her through some games, as well as measuring their height and weight. I would like to invite you to participate in this survey. Both interviews plus the games with the child combined will last about 1 hour.

Right to Refuse or Withdraw

Participation in this survey is voluntary and you can choose not to participate. If you decide not to participate or withdraw at any point, nothing will happen to you or your family.

You can withdraw your consent from the study at any time, either during this survey or after. No data will be submitted without your explicit consent, which is gathered at the beginning and end of the survey. If you wish in future to withdraw your information from the survey, or to take away your consent, you can do so at any time by contacting us at ghanasurvey@ifs.org.uk. This address will be monitored until the end of 2024.

For peer review only - http://bmjopen.bmj.com/site/about/guidelines.xhtml

You have the right to ask questions and have those questions answered. If you have any question about the study please ask me now. You can also call [NAME AND CONTACT NUMBER OF FIELD COORDINATOR] if you have any question later.

What are the possible benefits of taking part in this research?

There are no direct benefits to you from participating in the survey. However, the information you would provide will help us design effective programs to pre-school education in Ghana.

What are the disadvantages and/or risks of taking part in the study?

There are additional risks presented by the ongoing COVID-19 pandemic. Taking part in the survey will entail interacting with an interviewer which could increase the likelihood of you contracting the virus. We will take several measures to minimize this risk as much as possible. First, the interviewer will remain at least 2 meters away from you throughout the interview, to facilitate social distancing. Interviews will be conducted outside, if possible. You will be offered disposable masks and gloves before the interview, should you wish to wear them. The tablets we use to collect your responses will be sanitized both before and after each interview. All enumerators will have their temperature checked each day and asked to report on any symptoms they are experiencing. If enumerators feel ill, we will not send them to collect any interviews. Finally, our research team will be monitoring the local COVID-19 situation every day, and will not send enumerators to conduct any interviews if the risks of transmission are deemed unacceptably high. If you do feel ill after completing the survey, you should contact [insert up-to-date information about who to contact in local health system] . If you feel unwell, you should also contact our survey team on [NAME AND CONTACT NUMBER OF FIELD COORDINATOR] so we can take additional precautions to ensure the safety of the interviewing teams and other participants.

Privacy Notice

What is the legal basis for collecting this data?

The legal basis for collecting and processing this information in the United Kingdom is “legitimate interests” (Article 6(1)(f)) of the General Data Protection Regulation 2018. As a research organisation, we have an interest in collecting and processing your data in order to understand how pre-school programs improve child development, and the effectiveness of preschool programs when they are implemented at a large scale

How will we keep your information safe? Who will have access to it?

All information we collect is strictly confidential and will only ever be used for academic research purposes. We will not share information with anyone outside the project team, not even with your parents, friends, or any other person in the village. Even when we prepare a report about the program, we will not mention the name of any person or village that participated.

We will exert our upmost effort to ensure that the data you give us remains safe and secure and that only those on the project team will ever have access to it. While we are in Ghana collecting data, your data will be accessed by me, while I enter it into the tablet during our interview, and my supervisor and the field manager, whose role it is to check the quality of the data. While we are collecting data, your data will be stored on the secure servers of the SurveyCTO. Once data has been collected from all participants, we will delete the data from the SurveyCTO server and no-one from the data collection team will retain any access to your data.

At that point, your survey responses will be transferred outside Ghana to the United Kingdom, where it will be stored on secure servers at the Institute for Fiscal Studies. At a later date it may be transferred to and stored securely at the University of Pennsylvania and Yale. Only researchers working on this project will have access to identifiable data. Only de-identified data is used for analysis. The IFS has policies and controls in place that are

For peer review only - http://bmjopen.bmj.com/site/about/guidelines.xhtml

aligned with ISO27001 (an international standard in information management) to ensure that your data is not lost, accidentally destroyed, misused or disclosed, and is not accessed except by members of the research team in the proper performance of their duties. This means that all information is handled according to its classification (for example personal data, publicly available data) and that access to the data is restricted accordingly, both through electronic and physical means. Staff are trained to ensure awareness of the importance of data security and practical knowledge of how to keep data safe. In addition, back-ups of our data are stored offsite in a secure UK location, which is ISO27001 compliant. We will store your contact information separately from any other information we hold on you. We will securely store your contact information in order for us to contact you again in the future for further surveys. At the point where there is no chance of running a further survey, we will delete all identifiable data.

How long will we keep your data?

As we would like to look at long term trends and we are holding your data for research purposes, we have not set a limit on how long we would like to keep the information you give. However, at the point where there is no chance of running a further survey, we will delete all identifiable data.

As according to Economic and Social Research Council guidelines, once the study has concluded, the data (without personal information) will be archived for other researchers to access.

What will happen with the results of this research?

The information that we gather will be analysed by the research team. It will contribute to research reports and academic papers on children's development and pre-school program effectiveness in Ghana. The reports will be available to download for free from the Institute for Fiscal Studies' website (www.ifs.org.uk). The reports will never include any information from which it would be possible to identify you.

Your rights

The UK Data Protection Act (2018) provides the following rights for individuals:

- The right to be informed
- The right of access
- The right of rectification
- The right of erasure
- The right to restrict processing
- The right to data portability
- The right to object
- Rights in relation to automated decision making and profiling

As your data will be processed for scientific research purposes as detailed above, various exceptions from these rights apply. In particular, the right to access, the right to rectification, the right to restrict processing, and the right to object do not apply, provided the appropriate safeguards are in place. In addition, the right to erasure is not absolute, and does not apply if it would seriously impair processing necessary for scientific research.

However, as explained above, if you wish to submit a request for your data to be deleted, please contact us at ghanasurvey@ifs.org.uk and your data will not be used in the study.

Contact us - Who is conducting and funding the study?

The research is conducted by researchers at the Institute for Fiscal Studies, University College London, Yale University, University of Pennsylvania and University of Ghana. We have a lot of experience conducting research on early childhood development.

If you have any questions about the research or wish to raise a complaint, you can contact the research team whose contact information is below. If you wish to raise a complaint please contact the Principal Investigator of

For peer review only - http://bmjopen.bmj.com/site/about/guidelines.xhtml

the research project, Sonya Krutikova who is based at the Institute for Fiscal Studies in London UK, at ghanasurvey@ifs.org.uk. If you feel that your complaint has not been handled to your satisfaction please contact the Chair of the University College London Research Ethics Committee, at ethics@ucl.ac.uk. If you are concerned about how your personal information is being processed, please contact IFS Data Protection Officer in the first instance (DataProtectionOfficer@ifs.org.uk). If you remain unsatisfied, you may wish to contact the UK Information Commissioner's Office (https://ico.org.uk/).

Who to contact in the event of a complaint?

If you have a complaint about any aspect of this study, please contact IPA or principle investigators of the study using the contact details above, or by emailing ghanasurvey@ifs.org.uk.

The current research team and the organisations they currently work for includes the researchers named below. If the research team members change as a result of staffing changes, the team will only ever include bona fide researchers of similar experience level. If the researchers working on the project move to a different organisation where the work on this project can be continued, your data will be shared with that organisation. An up-to-date list of researchers and their organisations can be found here [url for project site once ready] or can be obtained by emailing ghanasurvey@ifs.org.uk.

Principal researcher:

Sonya Krutikova (IFS, sonya_k@ifs.org.uk)

Orazio Attanasio (Yale, orazio.attanasio@yale.edu)

Other researchers:

Britta Augsburg (IFS, britta_a@ifs.org.uk)
Angus Phimister (IFS, angus_p@ifs.org.uk)
E.Nketiah-Amponsah (University of Ghana, enamponsah@ug.edu.gh)
Sharon Wolf (University of Pennsylvania, wolfs@upenn.edu)

The research project is funded the USAID. The project has been approved by UCL Research Ethics Committee (ID number **21361/001**) [ENTER DETAILS OF OTHER APPROVED IRBs HERE].

IFS Data Protection Officer

Zoe Oldfield (email: zoe_o@ifs.org.uk)

For peer review only

S4. Information on study primary and secondary outcomes

Figure S4.1 provides additional details on primary and secondary outcomes of the study.

Figure S4.1. Study primary and secondary outcome information

For peer review only

For peer review only - http://bmjopen.bmj.com/site/about/guidelines.xhtml

Domain	Instruments	Scoring and other information
**Primary outcomes**		
**Child cognitive development**	International Development and Early Learning Assessment (IDELA) tool	• Score following official guidelines provided by IDELA<br>• Score using Item Response Theory (IRT) for each of the developmental domains measured by IDELA<br>• Construct one overall score using IRT, pooling all IDELA items
	Tasks from the Harvard Laboratory for Development Studies	• Score using IRT for each of the domains measured<br>• Pool all items and score using IRT<br>• Pool with IDELA items and create domain specific and pooled scores using IRT
**Child socio-emotional development**	Strengths and Difficulties Questionnaire (SDQ) administered to the primary care giver.	• Score following official guidelines provided by the tool developers<br>• Create sub-domain scores using IRT<br>• Create overall score using IRT
**Child health**	Height<br>Weight	• Height for age and Weight for age<br>• Height for weight z-score
**Secondary Outcomes**		
**Maternal mental health and knowledge of ECCE**		
Caregiver mental health	Kessler-10	• Construct a total raw score following tool developer's guidance<br>• Construct a score using IRT
	Warwick-Edinburgh Mental Wellbeing Scale (WEMWBS)	• Construct a total raw score following tool developer's guidance<br>• Construct a score using IRT
Caregiver knowledge of ECCE practices	Sub-set of items from the Knowledge of Infant Development Inventory (KIDI)	• Raw score for number correct items<br>• Factor score constructed using IRT
**Parent-child investment**		
Home Environment	Family Care Indicators Questionnaire (FCI) and the Home Observation for Measurement of the Environment (HOME)	• Construct a total raw score following tool developer's guidance<br>• Construct a score using IRT
Caregiver-child relationship	Child Parent Relationship Scale (CPRS)	• Construct a total raw score following tool developer's guidance<br>• Construct a score using IRT
Caregiver instructional practices	Observation checklist; caregiver report	• Factor scores constructed using IRT: separate factor scores for observed and caregiver report items and then overall factor combining observed and caregiver report items.
**Teacher knowledge, motivation and teaching quality**		
Classroom quality	Classroom observation checklist	• Raw score of nr of conditions fulfilled out of a list of 11 (e.g. there are books in the classroom, adequate lighting, backboard etc.)<br>• Factor score constructed using IRT
Teacher practices	Classroom observation checklist	• Separate raw scores and a combined raw score for number of practices observed out of a list of 15 relating to behavior management and instructional practice.<br>• Factor scores for each of the above constructed using IRT
Teacher burn-out	Maslach burn-out battery	• Construct a total raw score following tool developer's guidance<br>• Construct a score using IRT
Teacher mental health	Kessler-10	• Construct a total raw score following tool developer's guidance<br>• Construct a score using IRT
Teacher well-being	Warwick-Edinburgh Mental Wellbeing Scale (WEMWBS)	• Construct a total raw score following tool developer's guidance<br>• Construct a score using IRT
**WASH practices in the home**	Caregiver report (including list randomization for sensitive behaviour) and interviewer observation	• Availability of WASH infrastructure: toilet, handwashing facility (with soap)<br>• WASH practices:<br>○ Handwashing of the respondents and their family members, particularly the target child.<br>○ Sanitation behavior (such as open defecation) of family members
**Child health**	Mid-Upper Arm Circumferences (MUAC)	• Acute Malnutrition measured by Mid-Upper Arm Circumferences (MUAC), using cut-offs as recommended by WHO.

For peer review only

S5. Data Statement

WHO Data set

Primary Registry and Trial Identifying Number	ISRCTN15360698
Date of Registration in Primary Registry	17/12/2021
Secondary Identifying Numbers	AEARCTR-0008500
Source(s) of Monetary or Material Support	USAID and MRC
Primary Sponsor	The Institute for Fiscal Studies
Secondary Sponsor	N/A
Contact for Public Queries	Britta Augsbury: britta_a@ifs.org.uk<br>The Institute for Fiscal Studies<br>Tel: 020 7291 4800<br>7 Ridgmount Street<br>London<br>WC1E 7AE
Contact for Scientific Queries	Dr Sonya Krutikova (sonya_k@ifs.org.uk)<br>The Institute for Fiscal Studies<br>Tel: 020 7291 4800<br>7 Ridgmount Street<br>London<br>WC1E 7AE
Public Title	Evaluating an early childhood care and education program brought to scale in Ghana
Scientific Title	Lively Minds: Improving health and development through play - Evaluation of a comprehensive ECCE programme at scale in Ghana
Countries of Recruitment	Ghana
Health Condition(s) or Problem(s) Studied	Child development
Interventions	Play schemes for pre-school children.
Key Inclusion and Exclusion Criteria	1. Be aged between 3 and 5 years<br>2. Be enrolled or planning to enroll in the sample preschool in the coming term
Study Type	Interventional, open label cluster randomized control trial.

For peer review only - http://bmjopen.bmj.com/site/about/guidelines.xhtml

Date of First Enrolment	7/02/2022
Sample Size	3240 children
Recruitment Status	Incomplete
Primary Outcome	Measured 2 to 3 academic terms (6 - 9 months approximately) after treatment commences:<br>1. Child cognition as measured using the emergent numeracy, emergent literacy and executive functioning tasks in the IDELA as well as items relating to the same domains from the Harvard Laboratory for Development Studies<br>2. Child health, as measured using height/weight for age and height for weight Z score<br>3. Child socio-emotional development, as measured by the externalizing, internalizing and pro-social domains of the Strengths and Difficulty questionnaire and the socio-emotional items in the IDELA
Key Secondary Outcomes	Measured 2 to 3 academic terms (6 - 9 months approximately) after treatment commences:<br>1. Maternal mental health (Kessler-10 and WEMWBS) and knowledge of ECCE<br>2. Teacher knowledge, motivation and teaching quality (measured through classroom observations)<br>3. Parent-child investment, as measured by quality of the home environment (FCI and HOME) and the parent-child relationship (CPRS)<br>4. WASH practices in the home<br>5. Acute Malnutrition measured by MUAC
Ethics Review	Study protocols have been approved by ethics boards at University College London (IRB 21361/001), Yale University (IRB 2000031549) and Ghana Health Service Ethical Review Committee (IRB 028/09/21).
Completion date	September 2025
Summary Results	n/a as trial incomplete
IPD sharing statement	The data collected in the study will be publicly distributed along with critical documents (ie, protocols and questionnaires) following the publication of the primary results from the trials, which is expected to be within 24 months of the final data collection date.

For peer review only

Reporting checklist for protocol of a clinical trial.

Based on the SPIRIT guidelines.

Instructions to authors

Complete this checklist by entering the page numbers from your manuscript where readers will find each of the items listed below.

Your article may not currently address all the items on the checklist. Please modify your text to include the missing information. If you are certain that an item does not apply, please write "n/a" and provide a short explanation.

Upload your completed checklist as an extra file when you submit to a journal.

In your methods section, say that you used the SPIRITreporting guidelines, and cite them as:

Chan A-W, Tetzlaff JM, Gøtzsche PC, Altman DG, Mann H, Berlin J, Dickersin K, Hróbjartsson A, Schulz KF, Parulekar WR, Krleža-Jerić K, Laupacis A, Moher D. SPIRIT 2013 Explanation and Elaboration: Guidance for protocols of clinical trials. BMJ. 2013;346:e7586

For peer review only

		Reporting Item	Page Number
**Administrative information**			
Title	#1	Descriptive title identifying the study design, population, interventions, and, if applicable, trial acronym	1

Trial registration	#2a	Trial identifier and registry name. If not yet registered, name of intended registry	2
Trial registration: data set	#2b	All items from the World Health Organization Trial Registration Data Set	Supplementary Materials S5.
Protocol version	#3	Date and version identifier	12
Funding	#4	Sources and types of financial, material, and other support	11
Roles and responsibilities: contributorship	#5a	Names, affiliations, and roles of protocol contributors	1, 11
Roles and responsibilities: sponsor contact information	#5b	Name and contact information for the trial sponsor	11
Roles and responsibilities: sponsor and funder	#5c	Role of study sponsor and funders, if any, in study design; collection, management, analysis, and interpretation of data; writing of the report; and the decision to submit the report for publication, including whether they will have ultimate authority over any of these activities	11
Roles and responsibilities: committees	#5d	Composition, roles, and responsibilities of the coordinating centre, steering committee, endpoint adjudication committee, data management team,	n/a, no committees relevant

For peer review only

		and other individuals or groups overseeing the trial, if applicable (see Item 21a for data monitoring committee)	
**Introduction**			
Background and rationale	#6a	Description of research question and justification for undertaking the trial, including summary of relevant studies (published and unpublished) examining benefits and harms for each intervention	2-3
Background and rationale: choice of comparators	#6b	Explanation for choice of comparators	2-3
Objectives	#7	Specific objectives or hypotheses	3-4
Trial design	#8	Description of trial design including type of trial (eg, parallel group, crossover, factorial, single group), allocation ratio, and framework (eg, superiority, equivalence, non-inferiority, exploratory)	6
**Methods: Participants, interventions, and outcomes**			

For peer review only

Study setting	#9	Description of study settings (eg, community clinic, academic hospital) and list of countries where data will be collected. Reference to where list of study sites can be obtained	4
Eligibility criteria	#10	Inclusion and exclusion criteria for participants. If applicable, eligibility criteria for study centres and individuals who will perform the interventions (eg, surgeons, psychotherapists)	7
Interventions: description	#11a	Interventions for each group with sufficient detail to allow replication, including how and when they will be administered	4-6
Interventions: modifications	#11b	Criteria for discontinuing or modifying allocated interventions for a given trial participant (eg, drug dose change in response to harms, participant request, or improving / worsening disease)	n/a
Interventions: adherance	#11c	Strategies to improve adherence to intervention protocols, and any procedures for monitoring adherence (eg, drug tablet return; laboratory tests)	n/a, trial is evaluating effect of policy at scale
Interventions: concomitant care	#11d	Relevant concomitant care and interventions that are permitted or prohibited during the trial	n/a
Outcomes	#12	Primary, secondary, and other outcomes, including the specific measurement variable (eg,	7-8

For peer review only

		systolic blood pressure), analysis metric (eg, change from baseline, final value, time to event), method of aggregation (eg, median, proportion), and time point for each outcome. Explanation of the clinical relevance of chosen efficacy and harm outcomes is strongly recommended	
Participant timeline	#13	Time schedule of enrolment, interventions (including any run-ins and washouts), assessments, and visits for participants. A schematic diagram is highly recommended (see Figure)	6
Sample size	#14	Estimated number of participants needed to achieve study objectives and how it was determined, including clinical and statistical assumptions supporting any sample size calculations	6-7
Recruitment	#15	Strategies for achieving adequate participant enrolment to reach target sample size	6-7

Methods: Assignment of interventions (for controlled trials)

For peer review only

Allocation: sequence generation	#16a	Method of generating the allocation sequence (eg, computer-generated random numbers), and list of any factors for stratification. To reduce predictability of a random sequence, details of any planned restriction (eg, blocking) should be provided in a separate document that is unavailable to those who enrol participants or assign interventions	6
Allocation concealment mechanism	#16b	Mechanism of implementing the allocation sequence (eg, central telephone; sequentially numbered, opaque, sealed envelopes), describing any steps to conceal the sequence until interventions are assigned	n/a, open label trial
Allocation: implementation	#16c	Who will generate the allocation sequence, who will enrol participants, and who will assign participants to interventions	6
Blinding (masking)	#17a	Who will be blinded after assignment to interventions (eg, trial participants, care providers, outcome assessors, data analysts), and how	n/a
Blinding (masking): emergency unblinding	#17b	If blinded, circumstances under which unblinding is permissible, and procedure for revealing a participant's allocated intervention during the trial	n/a

For peer review only

Methods: Data collection, management, and analysis

Data collection plan	#18a	Plans for assessment and collection of outcome, baseline, and other trial data, including any related processes to promote data quality (eg, duplicate measurements, training of assessors) and a description of study instruments (eg, questionnaires, laboratory tests) along with their reliability and validity, if known. Reference to where data collection forms can be found, if not in the protocol	7-8 (& supplementary materials S4)
Data collection plan: retention	#18b	Plans to promote participant retention and complete follow-up, including list of any outcome data to be collected for participants who discontinue or deviate from intervention protocols	7-8
Data management	#19	Plans for data entry, coding, security, and storage, including any related processes to promote data quality (eg, double data entry; range checks for data values). Reference to where details of data management procedures can be found, if not in the protocol	7-8

For peer review only

Statistics: outcomes	#20a	Statistical methods for analysing primary and secondary outcomes. Reference to where other details of the statistical analysis plan can be found, if not in the protocol	9-10
Statistics: additional analyses	#20b	Methods for any additional analyses (eg, subgroup and adjusted analyses)	9-10
Statistics: analysis population and missing data	#20c	Definition of analysis population relating to protocol non-adherence (eg, as randomised analysis), and any statistical methods to handle missing data (eg, multiple imputation)	9-10
**Methods: Monitoring**			
Data monitoring: formal committee	#21a	Composition of data monitoring committee (DMC); summary of its role and reporting structure; statement of whether it is independent from the sponsor and competing interests; and reference to where further details about its charter can be found, if not in the protocol. Alternatively, an explanation of why a DMC is not needed	n/a
Data monitoring: interim analysis	#21b	Description of any interim analyses and stopping guidelines, including who will have access to these interim results and make the final decision to terminate the trial	n/a

For peer review only

Harms	#22	Plans for collecting, assessing, reporting, and managing solicited and spontaneously reported adverse events and other unintended effects of trial interventions or trial conduct	n/a
Auditing	#23	Frequency and procedures for auditing trial conduct, if any, and whether the process will be independent from investigators and the sponsor	n/a
**Ethics and dissemination**			
Research ethics approval	#24	Plans for seeking research ethics committee / institutional review board (REC / IRB) approval	2
Protocol amendments	#25	Plans for communicating important protocol modifications (eg, changes to eligibility criteria, outcomes, analyses) to relevant parties (eg, investigators, REC / IRBs, trial participants, trial registries, journals, regulators)	10-11
Consent or assent	#26a	Who will obtain informed consent or assent from potential trial participants or authorised surrogates, and how (see Item 32)	7, 10-11
Consent or assent: ancillary studies	#26b	Additional consent provisions for collection and use of participant data and biological specimens in ancillary studies, if applicable	n/a

For peer review only

Confidentiality	#27	How personal information about potential and enrolled participants will be collected, shared, and maintained in order to protect confidentiality before, during, and after the trial	8
Declaration of interests	#28	Financial and other competing interests for principal investigators for the overall trial and each study site	11
Data access	#29	Statement of who will have access to the final trial dataset, and disclosure of contractual agreements that limit such access for investigators	11
Ancillary and post trial care	#30	Provisions, if any, for ancillary and post-trial care, and for compensation to those who suffer harm from trial participation	n/a
Dissemination policy: trial results	#31a	Plans for investigators and sponsor to communicate trial results to participants, healthcare professionals, the public, and other relevant groups (eg, via publication, reporting in results databases, or other data sharing arrangements), including any publication restrictions	10-11
Dissemination policy: authorship	#31b	Authorship eligibility guidelines and any intended use of professional writers	11

For peer review only

Dissemination policy: reproducible research	#31c	Plans, if any, for granting public access to the full protocol, participant-level dataset, and statistical code	n/a

Appendices

Informed consent materials	#32	Model consent form and other related documentation given to participants and authorised surrogates	Supplementary Materials S2. and S3.
Biological specimens	#33	Plans for collection, laboratory evaluation, and storage of biological specimens for genetic or molecular analysis in the current trial and for future use in ancillary studies, if applicable	n/a

None The SPIRIT Explanation and Elaboration paper is distributed under the terms of the Creative Commons Attribution License CC-BY-NC. This checklist can be completed online using https://www.goodreports.org/, a tool made by the EQUATOR Network in collaboration with Penelope.ai

For peer review only
